# New antiproliferative germacranolides from *Carpesium divaricatum*†

**DOI:** 10.1039/c9ra00478e

**Published:** 2019-04-11

**Authors:** Tao Zhang, Qiu-Bo Zhang, Lu Fu, Ling-Yu Li, Li-Yan Ma, Jin-Guang Si, Hong-Wu Zhang, Jian-He Wei, Shi-Shan Yu, Zhong-Mei Zou

**Affiliations:** Institute of Medicinal Plant Development, Chinese Academy of Medical Sciences, Peking Union Medical College Beijing 100193 P. R. China zmzou@implad.ac.cn +86-10-57833290 +86-10-57833290; School of Pharmacy, Henan University of Traditional Chinese Medicine Zhengzhou 450008 P. R. China; Institute of Materia Medica, Chinese Academy of Medical Sciences, Peking Union Medical College Beijing 100150 P. R. China

## Abstract

Six new highly oxygenated (2–7) and one known (1) germacranolides were isolated from the whole plant of *Carpesium divaricatum*. The planar structures and relative configurations of the new compounds were determined by detailed spectroscopic analysis. The absolute configurations of 1 and 3 were established by circular dichroism (CD) and X-ray crystallographic analyses, and the stereochemistry of the new compounds 2 and 4–6 were determined by similar CD data to 1 and 3, respectively. All isolates were evaluated for their antiproliferative activities against three human tumor cell lines, and compounds 3 and 6 show antiproliferative activities against HeLa and Hep G2 cells with IC_50_ values of 4.13–8.37 μM. Intensive mechanism study showed that 3 caused cell-cycle arrest at the S/G2 phase and induced apoptosis in Hep G2 cells through a mitochondria-related pathway.

## Abstract 2

A new compound from *Carpesium divaricatum* has the potential to treat liver cancer through apoptosis induction and cell cycle arrest.

## Graphical abstract

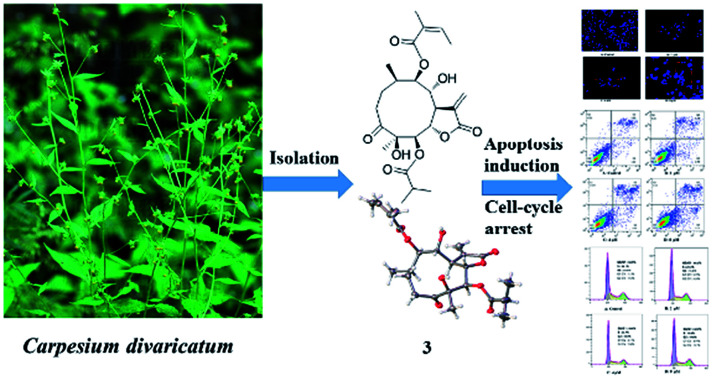

## Introduction

Lately, liver cancer has emerged as the most common malignant tumor in the digestive system, and is recognized as the second leading cancer responsible for mortality in men.^1^ China is a high incidence area of liver cancer, and its incidence rate is 1.5 times the world average level.^2^ Chemotherapy is an effective therapy for liver cancer in surgical treatment. However, the therapeutic effect is usually poor due to the low selectivity and toxicity of chemotherapy drugs.^3,4^

The genus *Carpesium* belongs to the family *Asteraceae* with 25 species distributed across Asia and Europe, particularly in southwest China.^5,6^ Sesquiterpenoids were considered as the characteristic constituents of this genus with diverse bioactivities such as cytotoxic, anti-inflammatory, and anti-parasitic activities.^6^ As an important member of this genus, *Carpesium divaricatum* Sieb.et Zucc is widely distributed in China, traditionally used for the treatment of fevers, colds, bruises, insect bites and inflammatory diseases.^7–12^ Previous investigations of this plant reported the isolation of germacranolides.^11–15^ The potential values of germacranolides to treat cancer and inflammatory diseases from the genera *Carpesium*, *Inula*, and *Allagopappus* have drawn increasing attention.^8,12–26^

In our continuing effort to search for bioactive constituents from *C. divaricatum*, highly oxygenated germacranolides attracted our attention due to their structural diversity. Seven highly oxygenated germacranolides including six new ones (2–7) were identified in the current investigation. These highly oxygenated germacranolides contains as many as eight stereogenic centers. NOESY spectrum, circular dichroism (CD) method and X-ray data analysis were used to confirm their relative and absolute configurations.

**Fig. 1:**
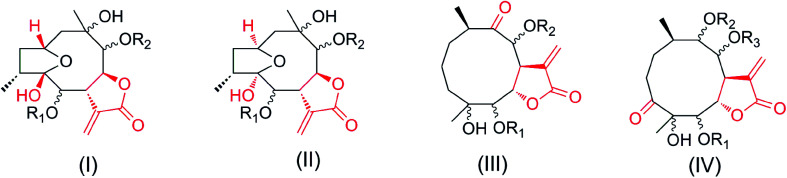
Four subtypes of germacranolides.

Notably, compounds 1–2 have opposite configurations at C-8 compared to subtype III we previously reported, indicating an incomplete structural formula of subtype III.^27^ In order to show the universality of structural formula, it is suggested that the configurations at C-4, C-5 and C-8 of subtype III should be depicted as shown (Fig. 1). Similarly, the main differences between compounds 3–6 and subtype IV, and compound 7 and subtype I are the interchange linkage groups at C-8/C-9 and the opposite configurations at C-10, respectively.^27^ The configurations at C-6/C-8/C-9 in subtypes I and II, the configurations at C-4/C-5/C-8/C-9, and the substituted groups at C-8/C-9 in subtype IV were revised as shown in this paper.

**Fig. 2:**
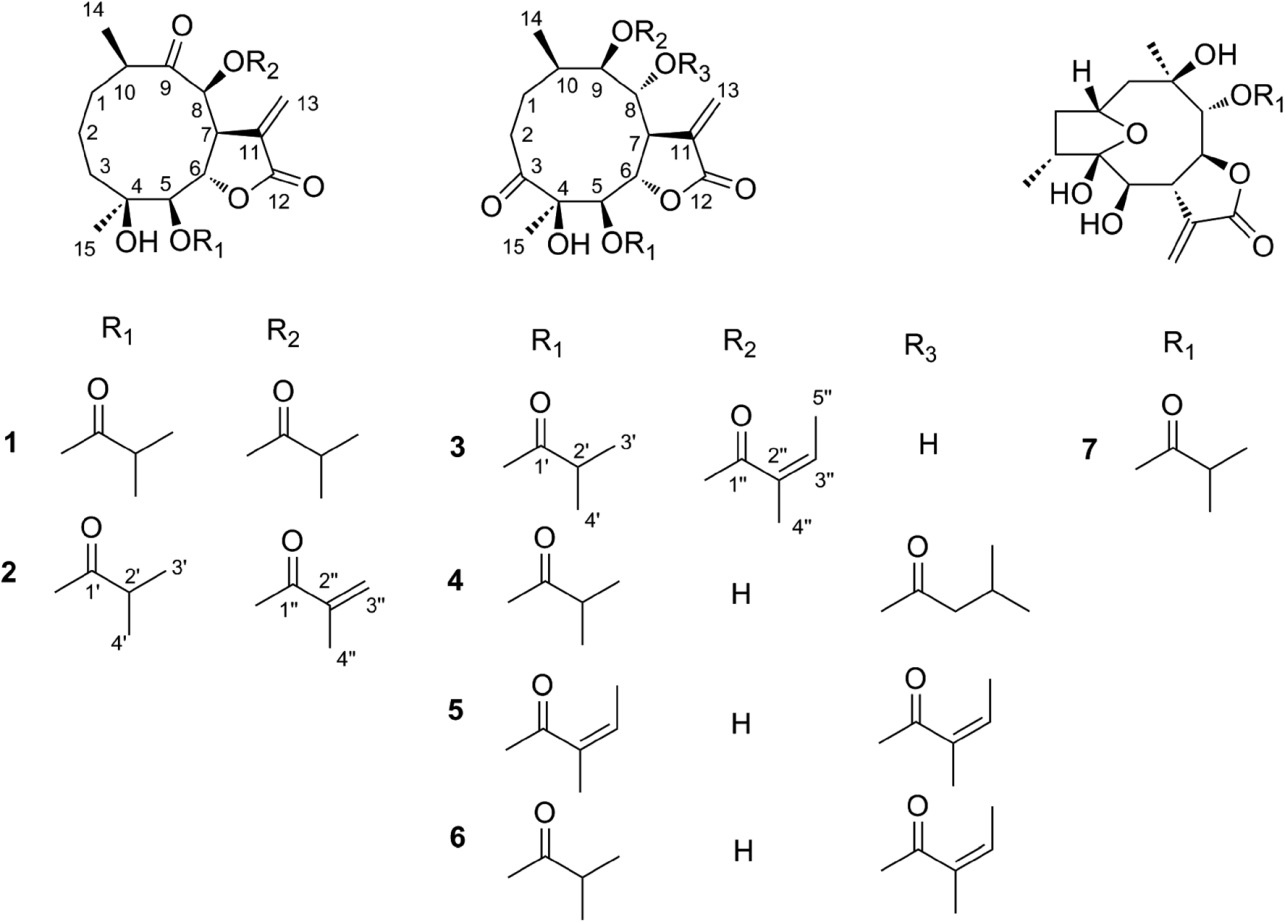
Chemical structures of compounds 1–7.

Furthermore, we screened the isolated compounds for the growth inhibition effect in three tumor cell lines, and then characterized the possible mechanism. It was found that 3 and 6 exhibited potent cytotoxicity against human cervical cancer (HeLa) and hepatocellular cancer (Hep G2) cell lines, respectively. Besides, compound 3 induced apoptosis and cell cycle arrest in Hep G2 cells Fig. 2.

## Results and discussion

### Structural elucidation

An 95% ethanol extract of the whole plant of *C. divaricatum* was subjected to repeated silica gel, MCI gel, Sephadex LH-20 and semi-prepare HPLC to afford six new highly oxygenated germacranolides (2–7) and one known analogue (1).

**Fig. 3:**
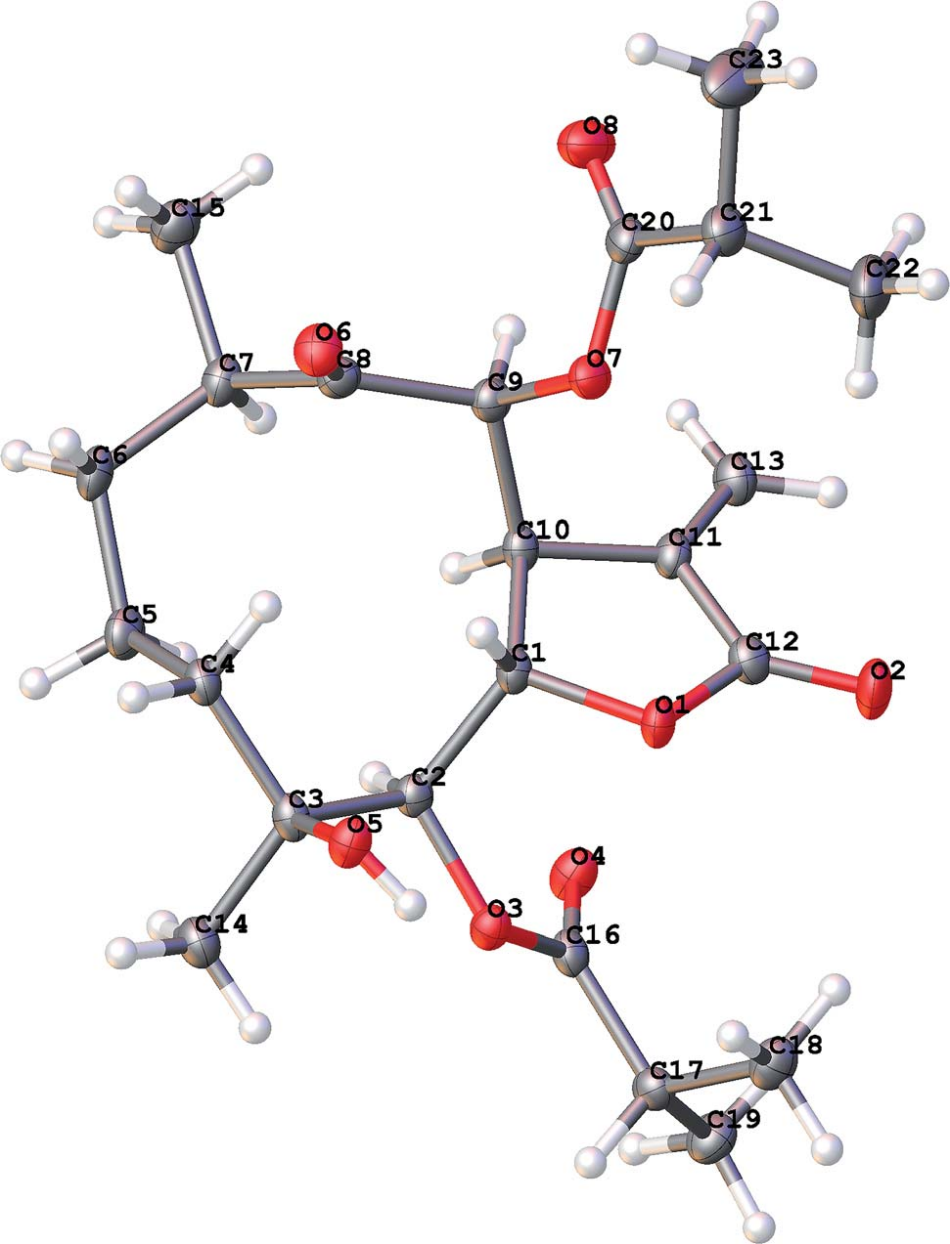
X-ray ORTEP drawing of 1.

Cernuumolide I (1) was identified by comparison of MS, NMR data, as well as optical rotation data with those reported.^28^ Its absolute configuration was further confirmed by CD spectrum (see ESI Fig. C1†) and X-ray diffraction (Fig. 3).

**Fig. 4:**
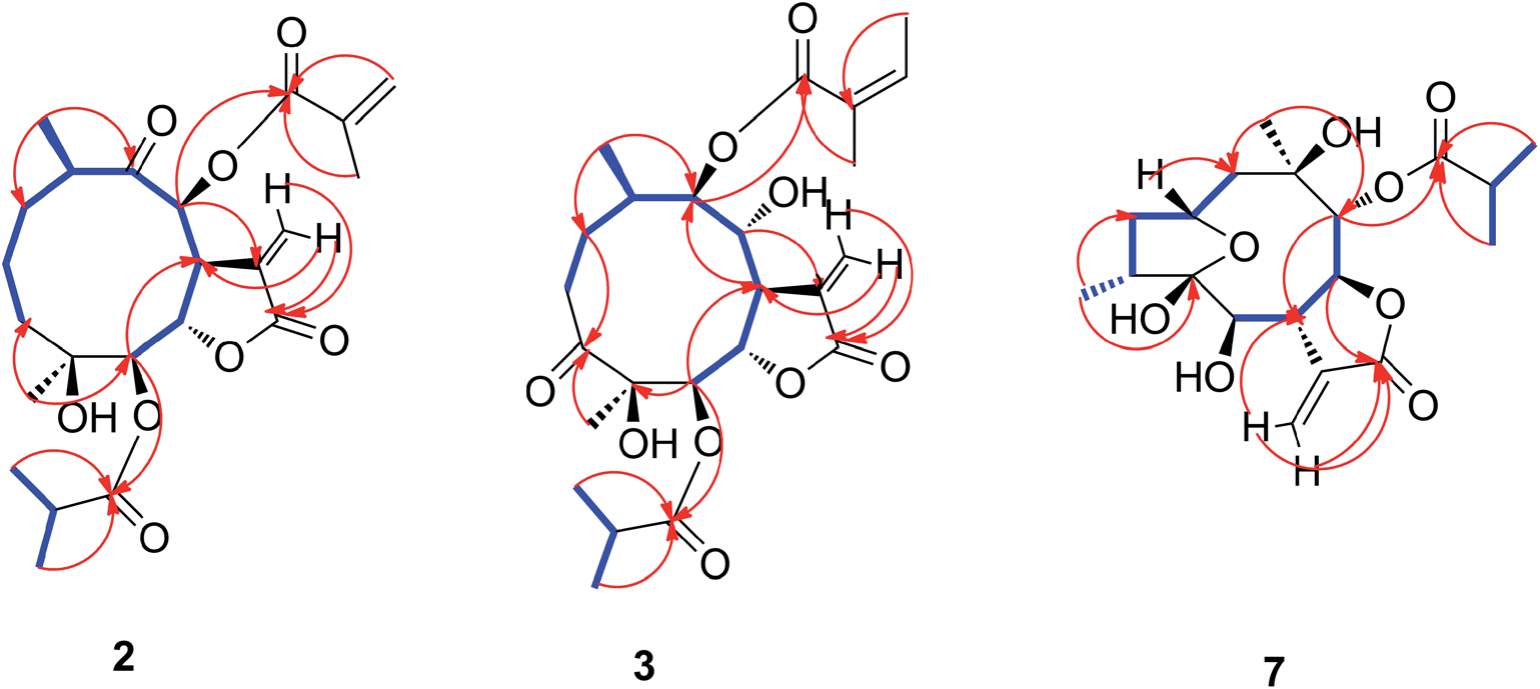
Key ^1^H–^1^H COSY and HMBC correlations of compounds 2, 3 and 7.

Compound 2 was obtained as white needles. The molecular formula was assigned as C_23_H_32_O_8_ on the basis of the positive-ion HRESIMS peak at *m*/*z* 459.1992 [M + Na]^+^, together with its ^1^H and ^13^C NMR data (Tables 1 and 2). Its IR spectrum showed hydroxyl (3514 cm^−1^) and carbonyl (1748 cm^−1^) absorptions. The ^1^H and ^13^C NMR spectra of 2 showed an α-methylene-γ-lactone at *δ*_H_ 6.34 (1H, d, *J* = 2.4 Hz, Ha-13) and 6.14 (1H, d, *J* = 2.4 Hz, Hb-13), *δ*_C_ 137.3 (C-11), 125.1 (C-13) and 171.3 (C-12); three carbonyl carbons at *δ*_C_ 211.3 (C-9), 179.0 (C-1′) and 167.0 (C-1′′); one oxygenated tertiary carbon at 73.7 (C-4); five methines including three oxygenated ones at *δ*_H_ 4.66 (1H, d, *J* = 6.6 Hz, H-5), 4.72 (1H, dd, *J* = 6.6, 1.8 Hz, H-6), 4.19 (1H, m, H-7), 5.46 (1H, d, *J* = 1.8 Hz, H-8) and 3.15 (1H, m, H-10), *δ*_C_ 79.1 (C-5), 73.5 (C-6), 45.4 (C-7), 83.4 (C-8), and 44.0 (C-10); and two methyl groups at *δ*_H_ 1.14 (3H, d, *J* = 7.2 Hz, CH_3_-14), 1.16 (3H, s, CH_3_-15). These signals (^1^H and ^13^C NMR data) implied that the structure of 2 was similar to that of 1 except that the isobutyryloxy group of 1 was replaced by a 2-methacryloyloxy group at C-8 in 2, which was further confirmed by the ^1^H–^1^H COSY, HSQC, and HMBC spectra (Fig. 4). On the basis of these data, the planar structure of 2 was established.

**Table 1:** ^1^H NMR spectroscopic data for compounds 2–7 (*δ* in ppm, *J* in Hz)

No.	2^a^	3^a^	4^b^	5^b^	6^b^	7^b^
1a	1.71 o^c^	1.88 m	1.89 m	1.90 m	1.94 m	2.59 m
1b	1.71 o	1.73 m	1.71 m	1.72 m	1.76 m	1.55 d (5.0)
2a	1.72 m	3.84 br d (11.4)	3.76 m	3.77 m	3.83 m	4.55 td (8.0,2.0)
2b	1.24 m	2.24 o	2.30 m	2.31 m	2.35 m	
3a	1.51 m					2.51 m
3b	1.33 m					1.29 m
4						2.26 m
5	4.66 d (6.6)	5.41 dd (9.6,1.8)	5.20 dd (8.0,2.0)	5.18 d (10.0)	5.30 d (10.0)	
6	4.72 dd (6.6, 1.8)	4.70 dd (9.6,6.6)	4.88 dd (8.0,4.0)	4.90 dd (10.0,5.0)	4.95 dd (10.0,5.0)	3.75 d (10.0)
7	4.19 m	3.07 m	3.11 m	3.14 m	3.17 m	3.26 m
8	5.46 d (1.8)	4.47 d (10.2)	5.56 d (8.5)	5.65 d (10.0)	5.68 d (10.0)	4.38 dd (10.0,10.0)
9		5.28 d (10.2)	3.70 d (8.5)	3.76 d (10.0)	3.77 d (10.0)	5.54 d (10.5)
10	3.15 m	2.24 o	2.06 m	2.09 m	2.11 m	
13a	6.34 d (2.4)	6.35 d (3.0)	6.33 d (3.0)	6.32 d (3.0)	6.34 d (3.0)	6.11 d (3.5)
13b	6.14 d (2.4)	5.72 d (3.0)	5.66 d (3.0)	5.70 d (3.0)	5.70 d (3.0)	6.05 d (3.5)
14	1.14 d (7.2)	1.00 d (6.6)	1.11 d (6.0)	1.11 d (6.5)	1.12 d (6.5)	1.20 s
15	1.16 s	1.25 s	1.22 s	1.22 s	1.25 s	1.04 d (6.5)
2′	2.68 m	2.72 m	2.69 m	2.68 m		
3′	1.20 d (7.2)	1.26 d (6.6)	1.22 d (6.0)	1.22 d (6.0)	6.08 qq (7.0,1.5)	
4′	1.18 d (7.2)	1.25 d (6.6)	1.23 d (6.0)	1.23 d (6.0)	1.97 q (1.5)	
5′					1.96 dq (7.0,1.5)	
2′′			2.16 m			2.61 m
3′′	6.08 dq (2.4,1.2),					
	5.67 dq (2.4,1.2)	6.17 qq (7.2,1.8)	1.98 m	6.09 qq (7.0,1.5)	6.17 qq (7.0,1.5)	1.20 d (5.5)
4′′	1.90 br s	1.97 dq (1.8,1.2)	0.89 d (5.0)	1.79 q (1.5)	1.80 q (1.5)	1.19 d (6.0)
5′′		2.01 dq (7.2,1.8)	0.90 d (5.0)	1.90 dq (7.0,1.5)	1.97 dq (7.0,1.5)	

a Measured at 600 MHz in methanol-*d*_4_.

b Measured at 500 MHz in methanol-*d*_4_.

c Overlapped with other signals.

**Table 2:** ^13^C NMR spectroscopic data for compounds 2–7 (*δ* in ppm)

No.	2^a^	3^a^	4^b^	5^b^	6^b^	7^b^
1	34.1	25.5	24.1	24.5	24.1	46.3
2	24.2	32.8	34.8	35.2	34.9	70.9
3	37.5	217.6	217.5	217.5	217.8	38.7
4	73.7	80.4	80.2	80.2	80.3	35.5
5	79.1	78.1	78.1	78.1	78.0	106.5
6	73.5	80.0	79.8	79.9	79.9	68.1
7	45.4	41.6	40.1	40.2	40.2	49.4
8	83.4	70.5	76.0	76.2	76.2	77.3
9	211.3	78.5	75.1	75.3	75.3	79.6
10	44.0	30.0	30.7	30.9	30.9	71.9
11	137.3	132.7	132.6	133.0	133.1	139.0
12	171.3	169.6	169.2	169.2	169.3	169.9
13	125.1	123.9	124.6	124.6	124.6	119.4
14	20.4	20.0	19.8	19.8	20.2	23.2
15	24.2	23.3	23.1	23.1	23.5	12.0
1′	179.0	176.4	176.4	176.4	167.1	
2′	35.0	34.0	33.9	34.0	127.4	
3′	19.2	18.0	18.0	18.0	138.2	
4′	19.2	17.9	17.9	17.9	19.2	
5′					14.6	
1′′	167.0	167.7	172.9	167.4	167.5	176.1
2′′	136.7	127.8	42.9	127.3	127.4	34.0
3′′	127.4	137.7	25.8	138.2	138.4	17.9
4′′	18.1	19.4	21.6	19.2	19.3	17.9
5′′		14.6	21.7	14.6	14.6	

a Measured at 150 MHz in methanol-*d*_4_.

b Measured at 125 MHz in methanol-*d*_4_.

**Fig. 5:**
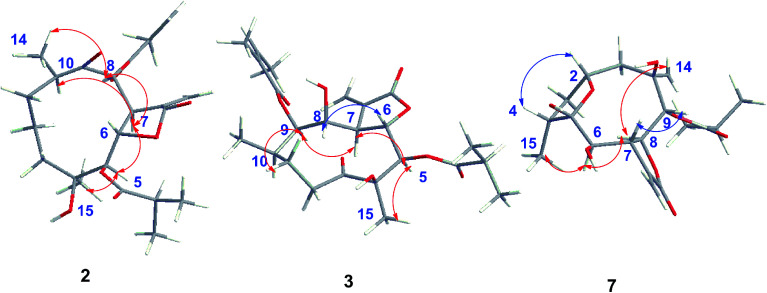
Key NOESY correlations of compounds 2, 3 and 7.

The relative configuration of 2 was determined by analysis of NOESY data. The key NOE correlations of H-8/H-7, H-7/H-5, H-7/H-10, H-8/H_3_-14, and H-5/H_3_-15 indicated that 2 had the same relative configuration as 1 (Fig. 5). The CD spectrum (see ESI Fig. C1†) of 2 exhibited two positive Cotton effects at near 254 nm (α-methylene-γ-lactone region) and 294 nm (ketone n, π* region), which closely resembled those of 1, supporting 6*S*,7*S* configuration.^25^ Similar NOESY and CD data of 2 and 1 assigned the absolute configuration of 2 as 4*S*,5*R*,6*S*,7*S*,8*S*, and 10*R*. Thus, the structure of compound 2 was determined as shown, named 8-isodivarolide C.

**Fig. 6:**
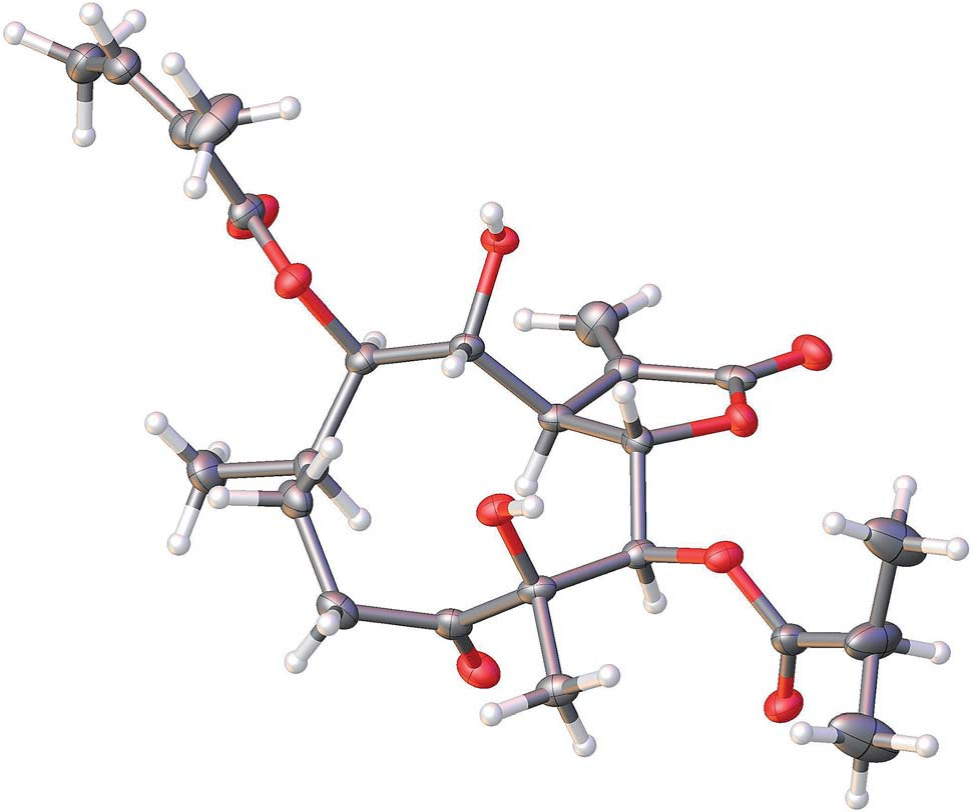
X-ray ORTEP drawing of 3.

The molecular formula of compound 3 was assigned as C_24_H_34_O_9_ by positive-ion HRESIMS ion at *m*/*z* 489.2101 [M + Na]. The ^1^H and ^13^C NMR data implied that the structure of 3 was similar to those of the known compound incaspitolide D,^27^ except that the angeloyloxy group at C-9 in 3 was observed in place of an isobutyryloxy group in incaspitolide D, which was further confirmed by the ^1^H–^1^H COSY, HSQC, and HMBC spectra (Fig. 4). The relative configuration of 3 was determined by analysis of ROESY data. The key NOE correlations of H-8/H-6, H-7/H-5, H-5/H_3_-15, H-7/H-9, and H-9/H-10 indicated that 3 had the same relative configuration as incaspitolide D (Fig. 5). The CD spectrum (see ESI Fig. C2†) of 3 exhibited two negative Cotton effects at near 220 nm (α-methylene-γ-lactone region) and 310 nm (ketone n, π* region), which closely resembled those of incaspitolide D, supporting 7*R* configuration.^19,29,30^ Fortunately, a suitable crystal was obtained for X-ray diffraction to confirm the absolute configuration. The X-ray crystallographic analysis [flack parameter: −0.07 (9)] established unambiguously the absolute configuration of 1 to be 4*R*,5*R*,6*S*,7*R*,8*R*,9*R*, and 10*R* (Fig. 6). Thus, the structure of compound 3 was established as shown, named cardivarolide H.

Compound 4 possessed molecular formula of C_24_H_36_O_9_ based on the HRESIMS ion at *m*/*z* 491.2249 [M + Na]^+^. A comparison of the ^1^H and ^13^C NMR data of 4 with those of 3 revealed strong similarity, except that the angeloyloxy group and the hydroxyl group in 3 were substituted by a hydroxyl group at C-9 and a 3-methylbutyryloxy group at C-8 in 4, respectively. The ^1^H–^1^H COSY, HSQC and HMBC spectra of 4 confirmed this observation, leading to the assignment of its planar structure. Compound 4 had the same relative configuration as 3, according to the analysis of their ROESY data. Similar CD data of 4 and 3 (see ESI Fig. C2†) revealed the same absolute configuration of 4 as that of 3. Thus, the structure of compound 4 was defined as shown, named cardivarolide I.

Compounds 5–6 had molecular formulas of C_24_H_34_O_9_, and C_25_H_34_O_9_ from their HRESIMS ions at *m*/*z* 489.2107 [M + Na]^+^, and *m*/*z* 501.2103 [M + Na]^+^, respectively. The ^1^H and ^13^C NMR data of 5 were similar to those of 4, except that the 3-methylbutyryloxy group at C-8 in 4 was replaced by an angeloyloxy group in 5. The NMR data of 6 were comparable with those of 5, except for the presence of an angeloyloxy group in 6 instead of the isobutyryloxy group at C-5 in 5. These observations were confirmed by analyses of relevant ^1^H–^1^H COSY, HSQC and HMBC data. The relative configurations of 5–6 were determined to be the same as that of 4 by comparison of ROESY data for relevant protons. Similar CD data of 5–6 and 4 (see ESI Fig. C2†) revealed the same absolute configurations of 5–6 as that of 4. Thus, the structures of compounds 5–6 were established as shown, named cardivarolide J and cardivarolide K, respectively.

The molecular formula of compound 7 was determined to be C_19_H_26_O_7_ by HRESIMS (389.1575 [M + Na]^+^). The ^1^H and ^13^C NMR data of 7 implied that the planar structure of 7 was closely related to the known compound (2*R*,5*S*)-cardivarolide C,^14,27^ but that the residue at C-6 was a hydroxy group in 7. The assignments were also supported by ^1^H–^1^H COSY, HSQC and HMBC spectra (Fig. 4). The relative configuration of 7 was nearly identical with those of (2*R*,5*S*)-cardivarolide C, except for C-10. In the NOESY spectrum, the key NOE correlations of H-2/H-4, H_3_-15/H-6, H-6/H-8, H-8/H_3_-14, and H-7/H-9 indicated 5-OH, H-7 and H-9 were attached to the β-side and H_3_-15, H-6, H-8 and H_3_-14 to the α-side of the ring (Fig. 5). The CD spectrum of 7 exhibited one positive Cotton effects at near 254 nm (α-methylene-γ-lactone region, see ESI Fig. S7†), which closely resembled those of ineupatolide,^19^ suggesting the 2*R*,4*R*,5*S*,6*R*,7*S*,8*S*,9*R*, and 10*R* absolute configuration for 7. Thus, the structure of 7 was elucidated as shown, named cardivarolide L.

### Cytotoxicity

All isolated compounds were evaluated for their *in vitro* cytotoxic activities against human cervical cancer (HeLa), hepatocellular cancer (Hep G2), and lung cancer (A549), using the MTT method with vorinostat as the positive control. The data in Table 3 suggested that new compounds 3 and 5 exhibited cytotoxicity against HeLa (IC_50_ values of 4.13 and 5.08 μM) and Hep G2 (IC_50_ values of 5.93 and 8.37 μM) cell lines compared to the positive control vorinostat (IC_50_ values of 10.90 and 8.82 μM), respectively. Considering the amounts of isolated compounds, we chose 3 for further research.

**Table 3:** Cytotoxicity of compounds 1–7^a^

Compound	IC_50_ (μM)		
	HeLa	Hep G2	A549
1	9.05 ± 0.80	14.03 ± 0.75	>50
2	>50	>50	>50
3	4.13 ± 0.75	5.93 ± 0.49	28.96 ± 1.01
4	6.18 ± 0.04	8.99 ± 1.45	42.73 ± 1.34
5	5.88 ± 0.05	10.66 ± 1.02	39.87 ± 1.17
6	5.08 ± 0.02	8.37 ± 0.22	23.67 ± 0.81
7	>50	>50	>50
Vorinostat	10.90 ± 3.00	8.82 ± 0.71	10.65 ± 0.46

a Values were mean ± SD. Vorinostat, positive control. Cell lines: HeLa: human cervical cancer, Hep G2: human hepatocellular cancer, A549: human lung cancer.

### Morphological evaluation of apoptosis

**Fig. 7:**
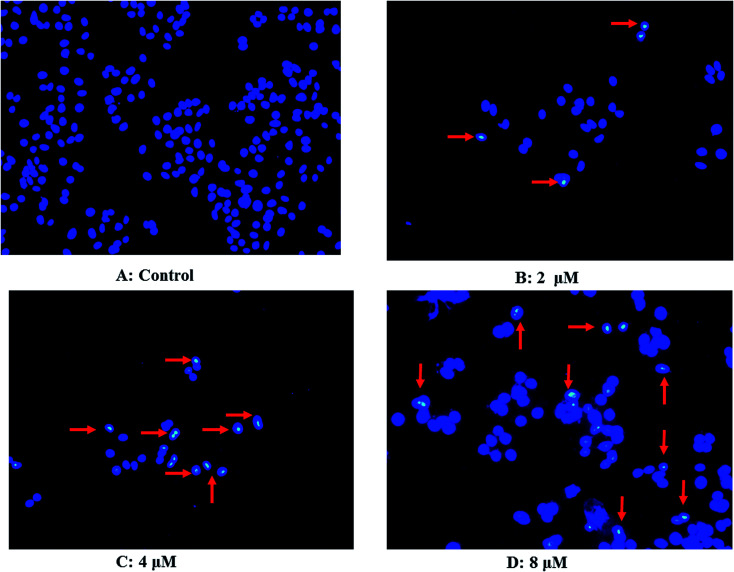
Chromatin condensation and nuclear fragmentation typical for apoptosis induction were visualized by fluorescence microscopy following Hoechst 33342 staining; (A) control Hep G2 cells; (B) Hep G2 cells treated with 2 μM 3; (C) Hep G2 cells treated with 4 μM 3; (D) Hep G2 cells treated with 8 μM 3. Magnification 400×.

As shown in Fig. 7, untreated Hep G2 cells exhibited normal growth characteristics (Fig. 7A). Interestingly, we found alterations in the structure, size, and shape of the nuclei in Hep G2 cells treated with 2.0 μM 3 for 48 h (Fig. 7B), with large cytoplasmic vacuoles, abnormal mitotic figures, multinucleation and formation of large cells being typical (Fig. 7C and D). These hallmarks of cell death, which increased concomitantly with increasing drug concentration, are indicated by red arrows.

### Cardivarolide H (3) induces apoptosis in Hep G2 cells

**Fig. 8:**
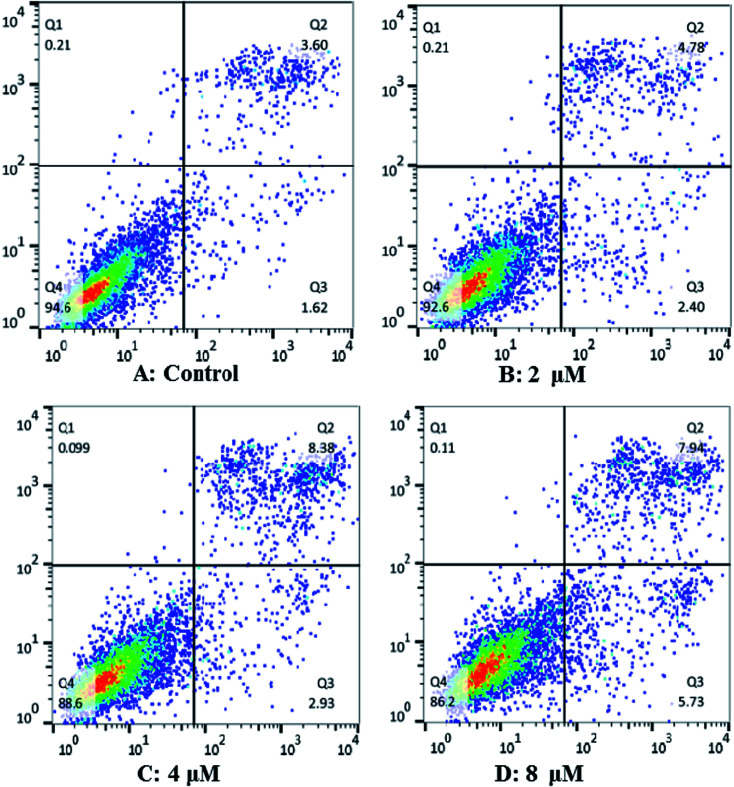
Cardivarolide H (3) induces apoptosis in Hep G2 cells. (A) Control Hep G2 cells; (B) Hep G2 cells treated with 2 μM 3; (C) Hep G2 cells treated with 4 μM 3; (D) Hep G2 cells treated with 8 μM 3.

In order to characterize the apoptosis process of Hep G2 cells induced by cardivarolide H (3), we examined the numbers of apoptotic cells by Annexin V-FITC/PI apoptosis staining. After 24 h exposure to different concentrations of 3, the numbers of apoptotic cells (AV+/PIand AV+/PI+) increased in a dose-dependent manner (Fig. 8). These results demonstrated that 3 was effective in the induction of apoptosis in Hep G2 cells.

### Cardivarolide H (3) increases G0/G1 arrest of cell cycle in Hep G2 cells

**Fig. 9:**
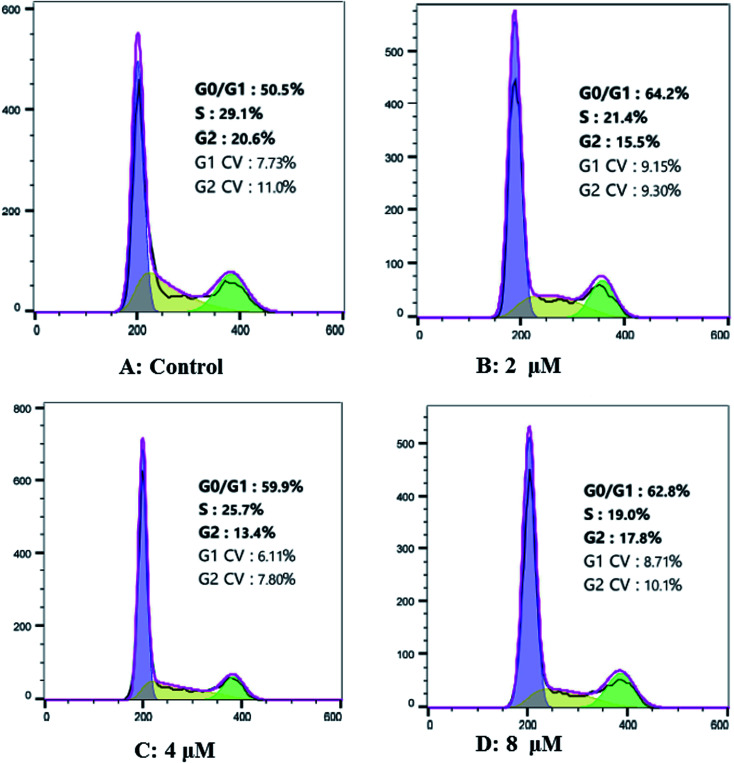
Effects of cardivarolide H (3) on cell cycle distribution in Hep G2 cells. (A) Control Hep G2 cells; (B) Hep G2 cells treated with 2 μM 3; (C) Hep G2 cells treated with 4 μM 3; (D) Hep G2 cells treated with 8 μM 3.

Hep G2 cells were treated with different concentrations of cardivarolide H (3) for 24 h, followed by flow cytometry analyses and the cardivarolide H-treated group revealed G0/G1 phase arrest compared with control group (Fig. 9). Fig. 9 revealed the numbers of cells arrested in G0/G1 phase. These results indicated that growth inhibition of 3 in Hep G2 cells were partly associated with the induction of G0/G1 arrest in cell cycle.

## Experimental section

### General experimental procedures

Optical rotations were measured on a Perkin-Elmer 241 polarimeter (Perkin-Elmer, Waltham, MA, USA) and UV spectra were recorded on Shimadzu UV-2501 PC (Shimadzu, Kyoto, Japan). IR data were recorded using a Shimadzu FTIR-8400S spectrophotometer (Shimadzu, Kyoto, Japan). ^1^H and ^13^C-NMR data were acquired with Bruker 600 and Bruker 500 instruments (Bruker, Rheinstetten, Germany) using the solvent signals as references. HRESIMS data were acquired using Q-TOF analyzer in SYNAPT HDMS system (Waters, Milford, MA, USA). CD spectra were recorded on a JASCO J-815 Spectropolarimeter (Jasco, Tokyo, Japan). X-ray diffraction data were collected on the Agilent GEMINI™E instrument (CrysAlisPro software, Version 1.171.35.11; Agilent, Santa Clara, CA, USA). HPLC was performed using Waters 2535 system (Waters, Milford, MA, USA) with the following components: preparative column, a Daisogel-C_18_-100A (10 μm, 30 × 250 mm, Chuang Xin Tong Heng Sci. &Tech., Beijing, China) and a YMC-Pack ODS-A column (5 μm, 10 × 250 mm, YMC, Kyoto, Japan); and detector, Waters 2489 UV. Sephadex LH-20 (40–70 μm, Pharmacia Biotech AB, Uppsala, Sweden), silica gel (60–100, 100–200, and 200–300 mesh) and silica gel GF254 sheets (0.20–0.25 mm) (Qingdao Marine Chemical Plant, Qingdao, China) were used for column chromatography and TLC, respectively. TLC spots were visualized under UV light and by dipping into 5% H_2_SO_4_ in EtOH followed by heating.

### Plant material

The whole plant of *C. divaricatum* was collected from Enshi, Hubei province of China, in August of 2013. They were identified by Prof. Ben-Gang Zhang of Institute of Medicinal Plant Development. A voucher specimen (no. 20130828) was deposited in the National Compound Library of Traditional Chinese Medicines, Institute of Medicinal Plant Development, Chinese Academy of Medical Sciences and Peking Union Medical College (CAMS & PUMC), China.

### Extraction and isolation

The air-dried plants (9 kg) were extracted three times (7 days each time) with EtOH–H_2_O (95 : 5) at room temperature. The combined extract was concentrated under reduced pressure to furnish a dark brown residue (570 g), which was suspended in H_2_O and partitioned in turn with petroleum ether (bp 60–90 °C), EtOAc, and *n*-BuOH.

The EtOAc extract (207 g) was separated chromatographically on silica gel column (60–100 mesh, 16 × 20 cm) with a gradient mixture of CH_2_Cl_2_–MeOH (100 : 1, 60 : 1, 30 : 1, 15 : 1, and 6 : 1) as eluent. Five fractions were collected according to TLC analysis. Fraction A (CH_2_Cl_2_–MeOH, 100 : 1, 140 g) was separated by silica gel column chromatography (CC) (100–200 mesh, 16 × 20 cm) with petroleum ether–acetone (50 : 1, 25 : 1, 20 : 1, 15 : 1, 12 : 1, 10 : 1, 7 : 1, 5 : 1, 3 : 1, and 1 : 1) as eluent to give fractions A_1_–A_11_. Fraction A_9_ (petroleum ether–acetone, 5 : 1, 30 g) was separated by Sephadex LH-20 CC (5 × 200 cm, MeOH) to give Fr.A_9_S_1_–Fr.A_9_S_3_. Fraction A_9_S_2_ (20 g) was then subjected to MCI gel CC (6 × 50 cm) with a gradient mixture of MeOH–H_2_O (60 : 40, 80 : 20, and 100 : 0, 4000 mL each) to give three fractions (Fr.A_9_S_2_M_1_–Fr.A_9_S_2_M_3_). Fraction A_9_S_2_M_2_ (10 g) was further separated chromatographically on silica gel column (100–200 mesh, 5 × 50 cm) with a gradient mixture of petroleum ether–acetone (10 : 1, 7 : 1, 5 : 1, 3.5 : 1, 2 : 1, and 1 : 1) as eluent, and a total of 200 fractions (Fr.A_9_S_2_M_2_-1–200, 50 mL each) were collected. Fraction A_9_S_2_M_2_-113–123 (1 g) were separated by preparative HPLC (20 mL min^−1^, 65% MeOH in H_2_O) and semipreparative HPLC (2 mL min^−1^, 60–90% MeOH in H_2_O for 50 min; 2 mL min^−1^, 40–80% MeCN in H_2_O for 40 min) to yield 3 (25 mg). Fraction A_10_ (petroleum ether–acetone, 3 : 1, 40 g) was separated by Sephadex LH-20 CC (5 × 200 cm, MeOH) to give Fr.A_10_S_1_–Fr.A_10_S_3_. Fraction A_10_S_2_ (20 g) was then subjected to MCI gel CC (6 × 50 cm) with a gradient mixture of MeOH–H_2_O (60 : 40, 80 : 20, and 100 : 0, 4000 mL each) to give three fractions (Fr.A_10_S_2_M_1_–Fr.A_10_S_2_M_3_). Fraction A_10_S_2_M_2_ (13 g) was further separated chromatographically on silica gel column (200–300 mesh, 5 × 50 cm) with a gradient mixture of CH_2_Cl_2_–MeOH (150 : 1, 100 : 1, 50 : 1, and 20 : 1) as eluent, and a total of 86 fractions (Fr.A_10_S_2_M_2_-1–86, 200 mL each) were collected. Fraction A_10_S_2_M_2_-34–50 (1.5 g) were separated by preparative HPLC (20 mL min^−1^, 70% MeOH in H_2_O) and semipreparative HPLC (2 mL min^−1^, 60–90% MeOH in H_2_O for 40 min, and followed 40–80% MeCN in H_2_O for 40 min) to yield 7 (5 mg).

The petroleum ether extract (140 g) was separated chromatographically on silica gel column (60–100 mesh, 16 × 20 cm) with a gradient mixture of petroleum ether–ethyl acetate (100 : 1, 60 : 1, 30 : 1, 15 : 1, 6 : 1, and 3 : 1) as eluent. Nine fractions were collected according to TLC analysis. Fraction PE-F (petroleum ether–ethyl acetate, 6 : 1, 16 g) was then subjected to MCI gel CC (6 × 50 cm) with a gradient mixture of MeOH–H_2_O (60 : 40, 80 : 20, and 100 : 0, 4000 mL each) to give three fractions (Fr.PE-F_1_–Fr. PE-F_3_). Fraction PE-F_2_ (10 g) was further separated chromatographically on silica gel column (100–200 mesh, 5 × 50 cm) with a gradient mixture of petroleum ether–acetone (100 : 1, 25 : 1, 10 : 1, and 2 : 1) as eluent, and a total of 4 fractions (Fr.PE-F_2_A_1_–Fr.PE-F_2_A_4_, 3000 mL each) were collected. Fraction Fr.PE-F_2_A_2_ (3 g) were separated by preparative HPLC (20 mL min^−1^, 80% MeOH in H_2_O) and semipreparative HPLC (2 mL min^−1^, 70–90% MeOH in H_2_O for 30 min, and followed 40–80% MeCN in H_2_O for 40 min) to yield 1 (35 mg), 2 (5 mg), 4 (5 mg), 5 (5 mg), and 6 (5 mg).

### Characterization of new compounds

#### 8-Isodivarolide C (2)

White needles (CH_3_OH), [*α*]^20^_D_ −48.0 (*c* 0.025, MeOH); UV (MeOH) *λ*_max_(log *ε*): 206 (3.72) nm, IR (neat) *ν*_max_: 3414, 2971, 1748, 1203 cm^−1^; CD (MeOH) 214 (Δ*ε* −0.529), 295 (Δ*ε* +0.251) nm; HRESIMS (pos.): *m*/*z* 459.1992 [M + Na]^+^ (calcd for C_23_H_32_O_8_Na, 459.1995); ^1^H NMR data, see Table 1, ^13^C NMR data, see Table 2.

#### Cardivarolide H (3)

White needles (CH_3_OH), [*α*]^20^_D_ −120.0 (*c* 0.075, MeOH); UV (MeOH) *λ*_max_(log *ε*): 215 (3.97) nm, IR (neat) *ν*_max_: 3430, 2968, 2921, 1653, 1636 cm^−1^; CD (MeOH) 215 (Δ*ε* −0.083), 308 (Δ*ε* −0.013) nm; HRESIMS (pos.): *m*/*z* 489.2101 [M + Na]^+^ (calcd for C_24_H_34_O_9_Na, 489.2101); ^1^H NMR data, see Table 1, ^13^C NMR data, see Table 2.

#### Cardivarolide I (4)

White needles (CH_3_OH), [*α*]^20^_D_ −102.0 (*c* 0.1, MeOH); UV (MeOH) *λ*_max_(log *ε*): 207 (4.18) nm, IR (neat) *ν*_max_: 3454, 2965, 1741, 1154 cm^−1^; CD (MeOH) 215 (Δ*ε* −0.193), 307 (Δ*ε* −0.035) nm; HRESIMS (pos.): *m*/*z* 491.2249 [M + Na]^+^ (calcd for C_24_H_36_O_9_Na, 491.2257); ^1^H NMR data, see Table 1, ^13^C NMR data, see Table 2.

#### Cardivarolide J (5)

White needles (CH_3_OH), [*α*]^20^_D_ −123.0 (*c* 0.1, MeOH); UV (MeOH) *λ*_max_(log *ε*): 214 (5.12) nm, IR (neat) *ν*_max_: 3464, 1749, 1157 cm^−1^; CD (MeOH) 215 (Δ*ε* −0.187), 308 (Δ*ε* −0.028) nm; HRESIMS (pos.): *m*/*z* 489.2101 [M + Na]^+^ (calcd for C_24_H_34_O_9_Na, 489.2107); ^1^H NMR data, see Table 1, ^13^C NMR data, see Table 2.

#### Cardivarolide K (6)

White needles (CH_3_OH), [*α*]^20^_D_ −89.0 (*c* 0.1, MeOH); UV (MeOH) *λ*_max_(log *ε*): 216 (5.53) nm, IR (neat) *ν*_max_: 3475, 2971, 1717, 1142 cm^−1^; CD (MeOH) 219 (Δ*ε* −0.183), 308 (Δ*ε* −0.024) nm; HRESIMS (pos.): *m*/*z* 501.2101 [M + Na]^+^ (calcd for C_25_H_34_O_9_Na, 501.2103); ^1^H NMR data, see Table 1, ^13^C NMR data, see Table 2.

#### Cardivarolide L (7)

White needles (CH_3_OH), [*α*]^20^_D_ +84.7 (*c* 0.150, MeOH); UV (MeOH) *λ*_max_(log *ε*): 200 (3.53) nm, IR (neat) *ν*_max_: 3438, 3390, 1636 cm^−1^; CD (MeOH) 208 (Δ*ε* +0.083), 232 (Δ*ε* −0.012) nm; HRESIMS (pos.): *m*/*z* 389.1575 [M + Na]^+^ (calcd for C_19_H_26_O_7_Na, 389.1576); ^1^H NMR data, see Table 1, ^13^C NMR data, see Table 2.

### X-ray crystal structure analysis

X-ray diffraction data were collected on the Agilent GEMINI™E instrument (CrysAlisPro software, Version 1.171.35.11), with enhanced Cu Kα radiation (*λ* = 1.54184 Å). The structure was solved by direct methods and refined by full-matrix least-squares techniques (SHELXL-97). All non-hydrogen atoms were refined with anisotropic thermal parameters. Hydrogen atoms were located by geometrical calculations and from positions in the electron density maps. Crystallographic data (excluding structure factors) for 1 and 3 in this paper have been deposited with the Cambridge Crystallographic Data Centre (deposition numbers CCDC 1585969 and 1841157).

A colorless orthorhombic crystal (0.25 × 0.14 × 0.13 mm) of 1 was grown from MeOH–CH_2_Cl_2_ (7 : 3). Crystal data: C_23_H_34_O_8_, *M* = 438.50, *T* = 104.4 K, orthorhombic, space group *P*2_1_2_1_2_1_, *a* = 5.61656 (10) Å, *b* = 18.3134 (3) Å, *c* = 22.9609 (6) Å, *α* = 90.00°, *β* = 90.00°, *γ* = 90.00°, *V* = 2361.72 (2) Å^3^, *Z* = 4, *ρ* = 1.233 mg mm^−3^, *μ*(Cu Kα) = 0.766 mm^−1^, measured reflections = 7993, unique reflections = 4456 (*R*_int_ = 0.0275), largest difference peak/hole = 0.222/−0.226 e Å^−3^, and flack parameter = 0.06 (11). The final *R* indexes [*I* > 2*σ*(*I*)] were *R*_1_ = 0.0384, and w*R*_2_ = 0.0973. The final *R* indexes (all data) were *R*_1_ = 0.0407, and w*R*_2_ = 0.1000. The goodness of fit on *F*^2^ was 1.025.

A colorless orthorhombic crystal (0.4 × 0.36 × 0.11 mm) of 3 was grown from CD_3_OD. Crystal data: C_24_H_34_O_9_, *M* = 466.51, *T* = 109.45 K, orthorhombic, space group *P*2_1_2_1_2_1_, *a* = 9.8307 (12) Å, *b* = 17.7908 (13) Å, *c* = 27.106 (2) Å, *α* = 90.00°, *β* = 90.00°, *γ* = 90.00°, *V* = 4740.8 (8) Å^3^, *Z* = 8, *ρ* = 1.307 mg mm^−3^, *μ*(Cu Kα) = 0.830 mm^−1^, measured reflections = 18 022, unique reflections = 8922 (*R*_int_ = 0.0306), largest difference peak/hole = 0.488/−0.371 e Å^−3^, and flack parameter = −0.07 (9). The final *R* indexes [*I* > 2*σ*(*I*)] were *R*_1_ = 0.0459, and w*R*_2_ = 0.1181. The final *R* indexes (all data) were *R*_1_ = 0.0494, and w*R*_2_ = 0.1213. The goodness of fit on *F*^2^ was 1.037.

### Reagents and antibodies

3-(4,5-Dimethylthiazol-2-yl)-2,5-diphenyltetrazolium bromide (MTT), RNase A, propidium iodide (PI) were purchased from Sigma-Aldrich (MO, USA). Dulbecco's Modified Eagle's Medium (DMEM), RPMI 1640 medium, trypsin, penicillin, streptomycin, fetal bovine serum (FBS) were purchased from Gibco (CA, USA).

The Annexin V-FITC apoptosis detection kit was obtained from Solarbio (Beijing, China).

### Cytotoxicity viability assays

The assay was run in triplicate. The HeLa, Hep G2 and A549 cell lines were obtained from the National Infrastructure of Cell Line Resource (Beijing, China). Cells were cultured in RPMI-1640 containing 10% FBS, 2 mmol per L glutamine, 100 U per mL penicillin, and 100 μg per mL streptomycin at 37 °C in a humidified atmosphere with 5% CO_2_. The cells were seeded at a density of 5 × 10^3^ cells per well in 96-well plates and allowed to attach for 24 h. The thiazolyl blue tetrazolium blue (MTT) assay was performed to quantify cell viability following treatment with the isolated compounds or reference compound vorinostat.^31^ After 48 h, 20 mL MTT (5 mg mL^−1^) solution was added for 4 h at 37 °C. Then, the supernatant was discarded and dimethylsulfoxide (DMSO) was added to dissolve the formazan product. The intensity was measured at a wavelength of 540 nm.

### Morphological analysis with fluorescence microscopy

Apoptosis is one of the major pathways leading to cell death, and is associated with classical morphologies, including chromatin condensation and nuclear fragmentation. To evaluate the apoptotic activity of 3, we performed nuclear staining with the DNA-binding dye, Hoechst-33342. Briefly, K562/A02 cells were plated into 6-well plates (1 × 10^5^ cells in 3 mL) and treated with 0, 2.0, 4.0 or 6.0 μM of 3 for 48 h. Cells were collected by centrifugation at 1000 × *g* for 5 min, washed with ice-cold PBS and then incubated with Hoechst-33342 (10 μg mL^−1^) for 15 min in the dark, then placed on slides, and observed under a fluorescence microscope (excitation 346 nm, emission 460 nm; NIKON TE2000-E). Apoptotic cells were identified by condensation of chromatin and fragmentation of nuclei. Pictures were obtained using a video camera Q-imaging (Burnaby, BC, Canada).

### Apoptosis analysis

Cardivarolide H (3) induced apoptosis in Hep G2 cells were detected using Annexin V-FITC/PI apoptosis staining by flow cytometry. Cells were plated and treated with 3 (0, 2, 4, 8 μM) for 24 h. After harvested and washed twice with cold PBS, the cells were incubated with Annexin V in binding buffer for 10 min at room temperature in the dark, followed by PI for 5 min. Stained cells were detected and analyzed using FACS Calibur flow cytometry (Becton Dickinson, USA). Apoptotic rates were reported as the percentage of apoptotic cells among total cells.

### Cell cycle analysis

Cell cycle distribution was measured by staining DNA with PI. Cells (1 × 10^6^) were seeded in 6-well plates and treated with 3 (0, 2, 4, 8 μM) for 24 h. Then cells were harvested and fixed with 70% ethanol at −20 °C overnight. After washing twice with PBS, the cells were treated with RNase A for 20 min and then stained with PI (50 mg L^−1^) for 10 min in the dark at room temperature.^32^ The distribution of each phase in the cell cycle measured by DNA content was detected using FACS Calibur flow cytometry and analyzed by ModFit LT 4.0 software.

## Conclusion

In this work, six new highly oxygenated germacranolides (2–7), as well as one known analog (1) were isolated from the whole plant of *C. divaricatum*. Notably, a pair of isomers (3/5) was obtained from the same plant. The absolute configurations of 1 and 3 were unambiguously established by X-ray diffraction. The other compounds with the same skeleton were determined by comparison of NOESY and CD data with those of 1 and 3, respectively. To the best of our knowledge, it was the first report of the substituted groups linkage to C-8 of subtype IV, and 7 is the first 14α-methylgermacranolide of subtypes I and II. These findings are an important addition to the present knowledge on the germacranolide family. After screening, we found 3 and 6 showed significant cytotoxicity against two tumor cell lines, respectively. Further studies indicate that the possible mechanism of 3, was associated with reduced proliferation, induction of apoptosis and G0/G1 phase arrest, as revealed by colorimetric tests, morphological analysis, and flow cytometry. Therefore, novel compound 3 could be treated as a potential candidate for anticancer agents for further study.

## Conflicts of interest

The authors declare no conflict of interest.

## Supplementary Material

RA-009-C9RA00478E-s001

RA-009-C9RA00478E-s002
