# Supplementary material for: New antiproliferative germacranolides from Carpesium divaricatum
Source: RSC Adv. 2019 Apr 11;9(20):11493–502. doi: 10.1039/c9ra00478e (PMC9063262; doi:10.1039/c9ra00478e)
Supplement: RA-009-C9RA00478E-s001 [file RA-009-C9RA00478E-s001.pdf]

Electronic Supplementary Material (ESI) for RSC Advances.
This journal is © The Royal Society of Chemistry 2019

Supporting Information

New antiproliferative germacranolides from *Carpesium divaricatum*

Tao Zhang,[1#] Qiu-Bo Zhang,[1#] Lu Fu,[1, 2] Ling-Yu Li,[1] Li-Yan Ma,[1] Jin-Guang Si,[1] Hong-Wu Zhang,[1] Jian-He Wei,[1] Shi-Shan Yu,[3] and Zhong-Mei Zou[1*]

[1] Institute of Medicinal Plant Development, Chinese Academy of Medical Sciences and Peking Union Medical College, Beijing 100193, P. R. China.

[2] School of Pharmacy, Henan University of Traditional Chinese Medicine, Zhengzhou 450008, P. R. China.

[3] Institute of Materia Medica, Chinese Academy of Medical Sciences and Peking Union Medical College, Beijing 100150, P. R. China.

# These authors contributed equally to this study.

* Corresponding Author

Zhong-Mei Zou, Tel/Fax: 86-10-57833290,

E-mail address: zmzou@implad.ac.cn.

Contents of Supplementary Information

	contents
Fig.C1	CD spectra of compounds **1–2**
Fig.C2	CD spectra of compounds **3–6**
Fig. S1.1	^{1}H NMR spectrum (500 MHz) of compound **1** in CD_3OD
Fig. S1.2	^{13}C NMR spectrum (125 MHz) of compound **1** in CD_3OD
Fig. S1.3	^{1}H-^{1}H COSY spectrum (500 MHz) of compound **1** in CD_3OD
Fig. S1.4	HSQC spectrum (500 MHz) of compound **1** in CD_3OD
Fig. S1.5	HMBC spectrum (500 MHz) of compound **1** in CD_3OD
Fig. S1.6	ROESY spectrum (500 MHz) of compound **1** in CD_3OD
Fig. S1.7	HRESIMS spectrum of compound **1**
Fig. S1.8	CD spectrum of compound **1**
Fig. S1.9	X-ray ORTEP drawing of **1**
Fig. S2.1	^{1}H NMR spectrum (600 MHz) of compound **2** in CD_3OD
Fig. S2.2	^{13}C NMR spectrum (150 MHz) of compound **2** in CD_3OD
Fig. S2.3	^{1}H-^{1}H COSY spectrum (600 MHz) of compound **2** in CD_3OD
Fig. S2.4	HSQC spectrum (600 MHz) of compound **2** in CD_3OD
Fig. S2.5	HMBC spectrum (600 MHz) of compound **2** in CD_3OD
Fig. S2.6	NOESY spectrum (600 MHz) of compound **2** in CD_3OD
Fig. S2.7	UV spectrum of compound **2**
Fig. S2.8	IR spectrum of compound **2**
Fig. S2.9	HRESIMS spectrum of compound **2**
Fig. S2.10	CD spectrum of compound **2**
Fig. S3.1	^{1}H NMR spectrum (600 MHz) of compound **3** in CD_3OD
Fig. S3.2	^{13}C NMR spectrum (150 MHz) of compound **3** in CD_3OD
Fig. S3.3	^{1}H-^{1}H COSY spectrum (600 MHz) of compound **3** in CD_3OD
Fig. S3.4	HSQC spectrum (600 MHz) of compound **3** in CD_3OD
Fig. S3.5	HMBC spectrum (600 MHz) of compound **3** in CD_3OD
Fig. S3.6	ROESY spectrum (600 MHz) of compound **3** in CD_3OD
Fig. S3.7	UV spectrum of compound **3**
Fig. S3.8	IR spectrum of compound **3**
Fig. S3.9	HRESIMS spectrum of compound **3**
Fig. S3.10	CD spectrum of compound **3**
Fig. S3.11	X-ray ORTEP drawing of **3**
Fig. S4.1	^{1}H NMR spectrum (500 MHz) of compound **4** in CD_3OD
Fig. S4.2	^{13}C NMR spectrum (125 MHz) of compound **4** in CD_3OD
Fig. S4.3	^{1}H-^{1}H COSY spectrum (500 MHz) of compound **4** in CD_3OD
Fig. S4.4	HSQC spectrum (500 MHz) of compound **4** in CD_3OD
Fig. S4.5	HMBC spectrum (500 MHz) of compound **4** in CD_3OD
Fig. S4.6	NOESY spectrum (600 MHz) of compound **4** in CD_3OD
Fig. S4.7	UV spectrum of compound **4**
Fig. S4.8	IR spectrum of compound **4**

Fig. S4.9	HRESIMS spectrum of compound **4**
Fig. S4.10	CD spectrum of compound **4**
Fig. S5.1	^{1}H NMR spectrum (500 MHz) of compound **5** in CD_3OD
Fig. S5.2	^{13}C NMR spectrum (125 MHz) of compound **5** in CD_3OD
Fig. S5.3	^{1}H-^{1}H COSY spectrum (500 MHz) of compound **5** in CD_3OD
Fig. S5.4	HSQC spectrum (500 MHz) of compound **5** in CD_3OD
Fig. S5.5	HMBC spectrum (500 MHz) of compound **5** in CD_3OD
Fig. S5.6	ROESY spectrum (500 MHz) of compound **5** in CD_3OD
Fig. S5.7	UV spectrum of compound **5**
Fig. S5.8	IR spectrum of compound **5**
Fig. S5.9	HRESIMS spectrum of compound **5**
Fig. S5.10	CD spectrum of compound **5**
Fig. S6.1	^{1}H NMR spectrum (500 MHz) of compound **6** in CD_3OD
Fig. S6.2	^{13}C NMR spectrum (125 MHz) of compound **6** in CD_3OD
Fig. S6.3	^{1}H-^{1}H COSY spectrum (500 MHz) of compound **6** in CD_3OD
Fig. S6.4	HSQC spectrum (500 MHz) of compound **6** in CD_3OD
Fig. S6.5	HMBC spectrum (500 MHz) of compound **6** in CD_3OD
Fig. S6.6	ROESY spectrum (500 MHz) of compound **6** in CD_3OD
Fig. S6.7	UV spectrum of compound **6**
Fig. S6.8	IR spectrum of compound **6**
Fig. S6.9	HRESIMS spectrum of compound **6**
Fig. S6.10	CD spectrum of compound **6**
Fig. S7.1	^{1}H NMR spectrum (500 MHz) of compound **7** in CD_3OD
Fig. S7.2	^{13}C NMR spectrum (125 MHz) of compound **7** in CD_3OD
Fig. S7.3	^{1}H-^{1}H COSY spectrum (500 MHz) of compound **7** in CD_3OD
Fig. S7.4	HSQC spectrum (500 MHz) of compound **7** in CD_3OD
Fig. S7.5	HMBC spectrum (500 MHz) of compound **7** in CD_3OD
Fig. S7.6	ROESY spectrum (600 MHz) of compound **7** in CD_3OD
Fig. S7.7	UV spectrum of compound **7**
Fig. S7.8	IR spectrum of compound **7**
Fig. S7.9	HRESIMS spectrum of compound **7**
Fig. S7.10	CD spectrum of compound **7**

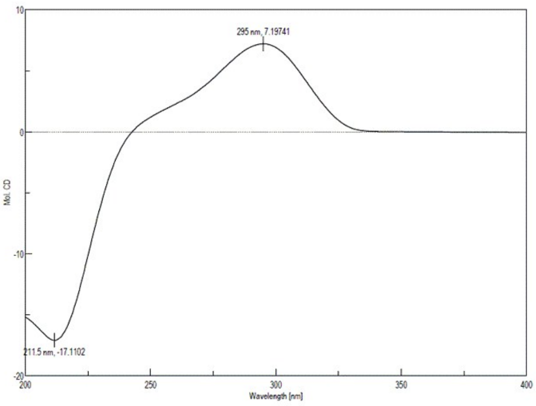

1

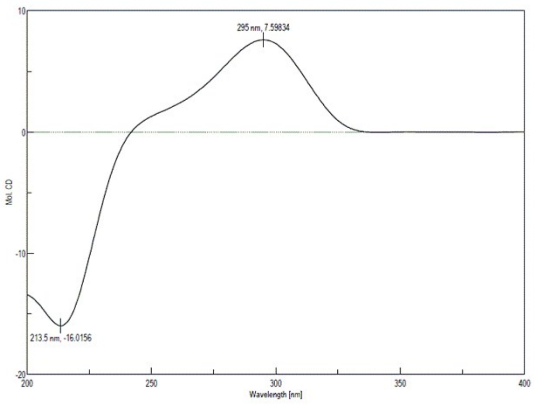

2

Fig.C1 CD spectra of compounds **1**–**2**

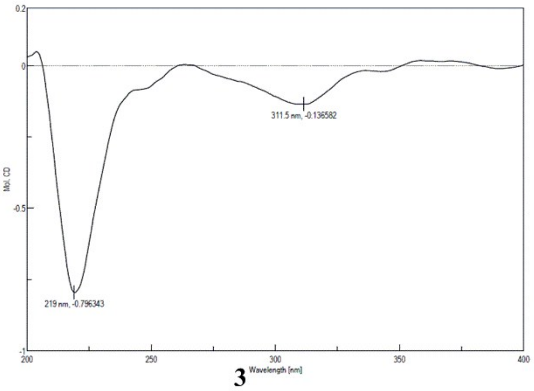

3

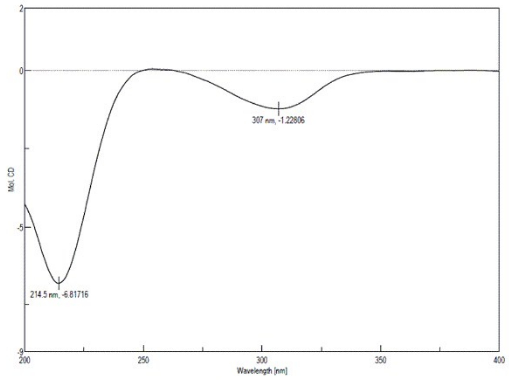

4

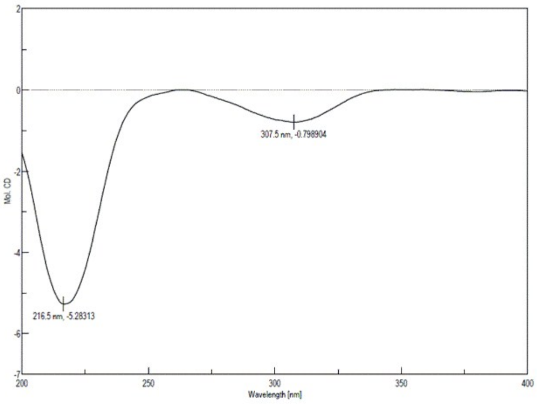

5

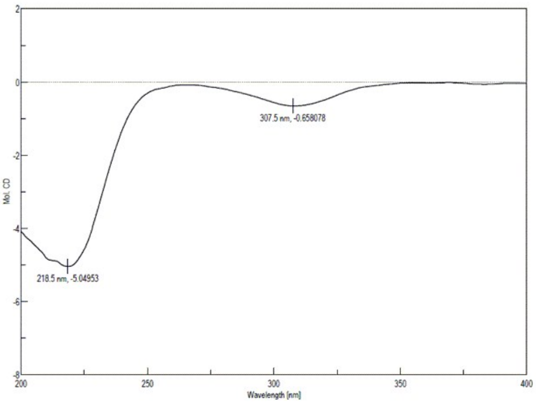

6

Fig.C2 CD spectra of compounds **3**–**6**

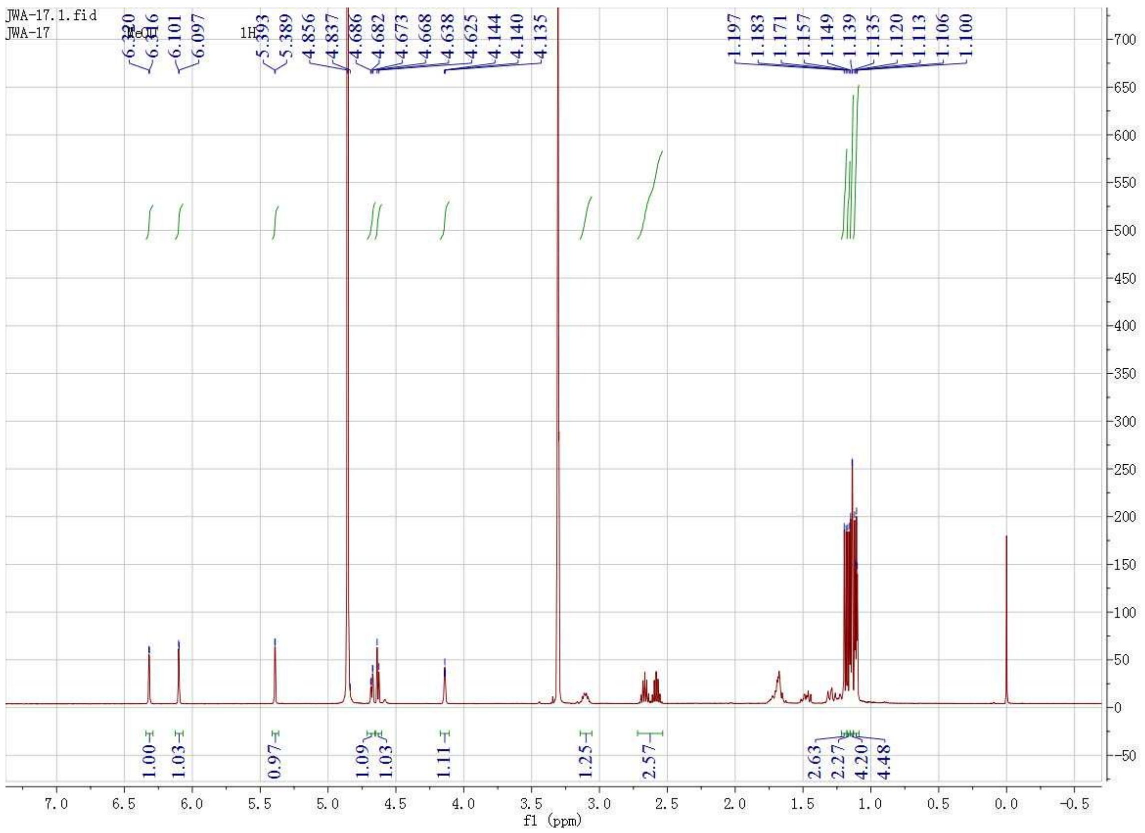

Fig. S1.1 ^{1}H NMR spectrum (500 MHz) of compound **1** in CD_3OD

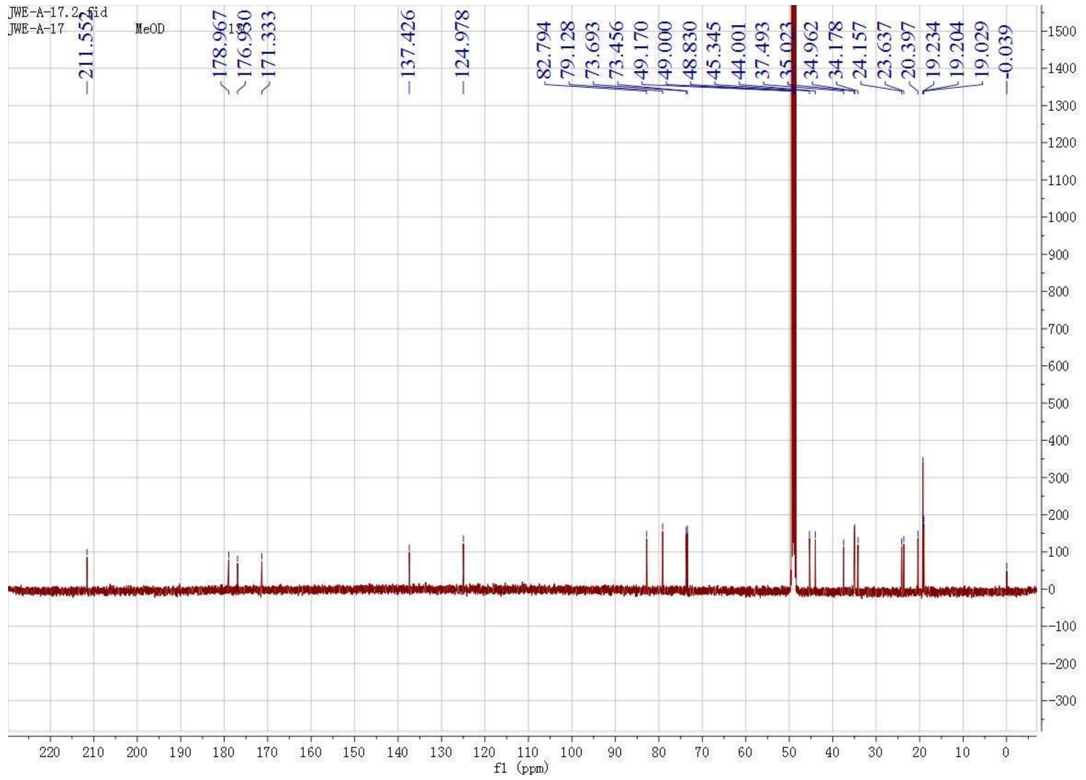

Fig. S1.2 ^{13}C NMR spectrum (125 MHz) of compound **1** in CD_3OD

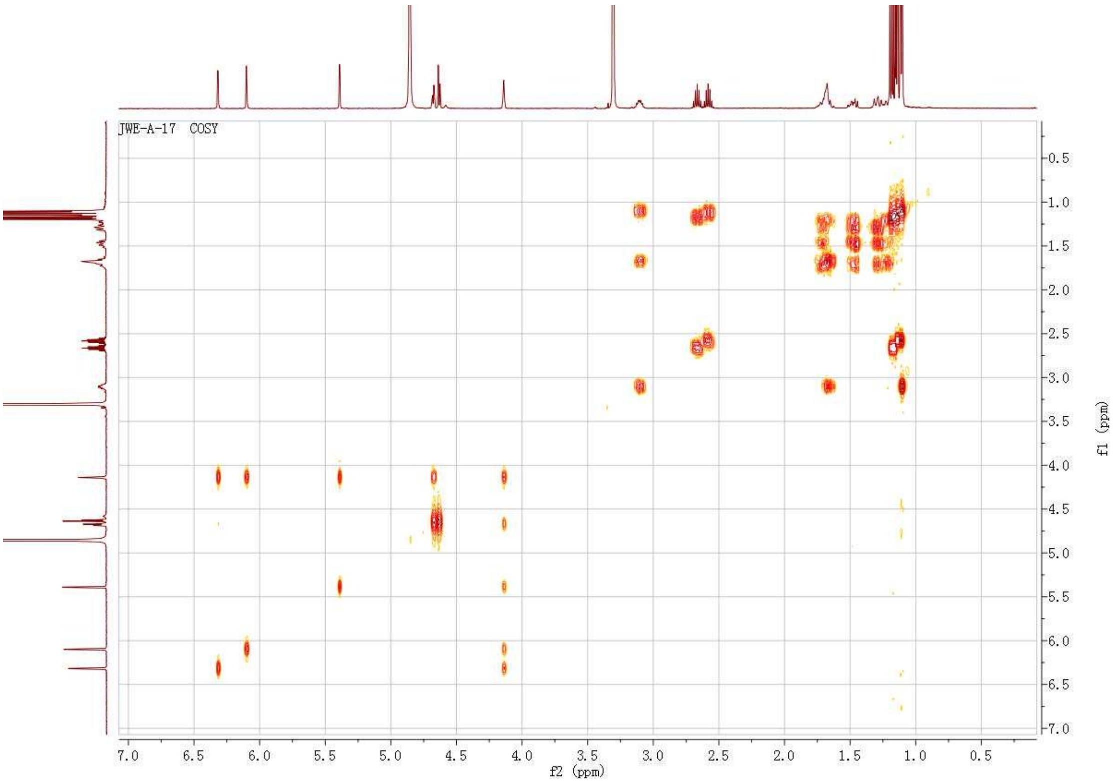

Fig. S1.3 ^{1}H-^{1}H COSY spectrum (500 MHz) of compound **1** in CD_3OD

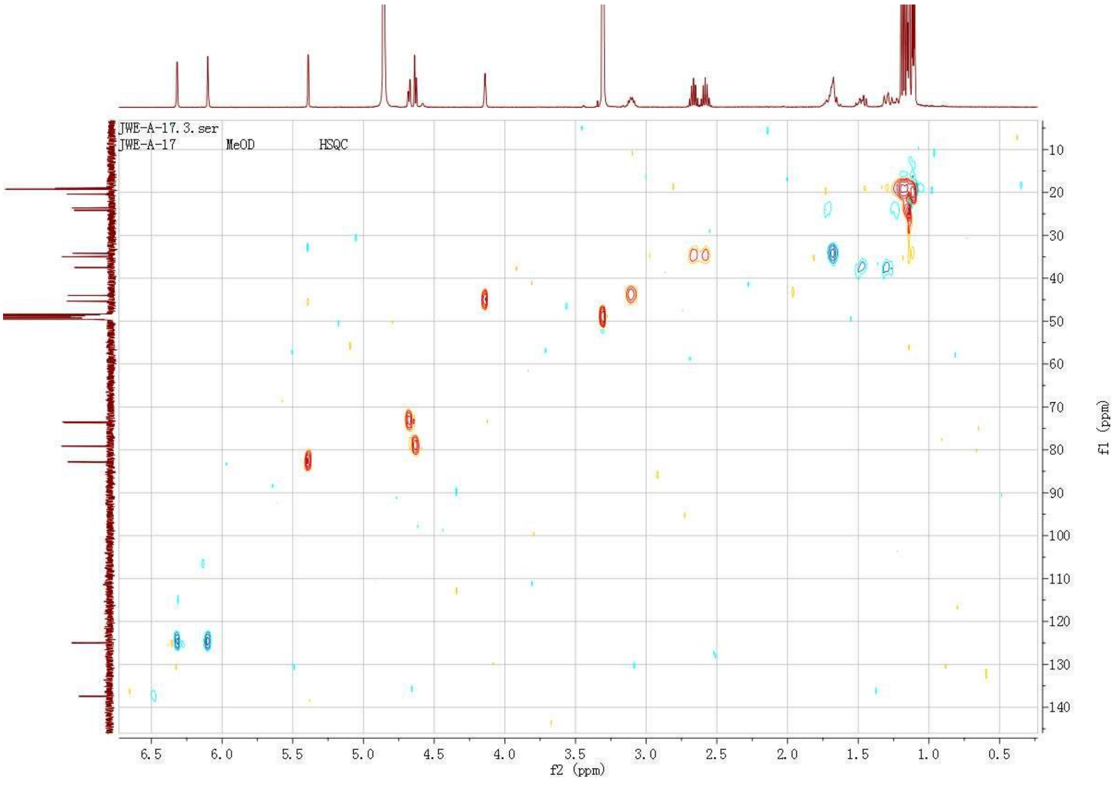

Fig. S1.4 HSQC spectrum (500 MHz) of compound **1** in CD_3OD

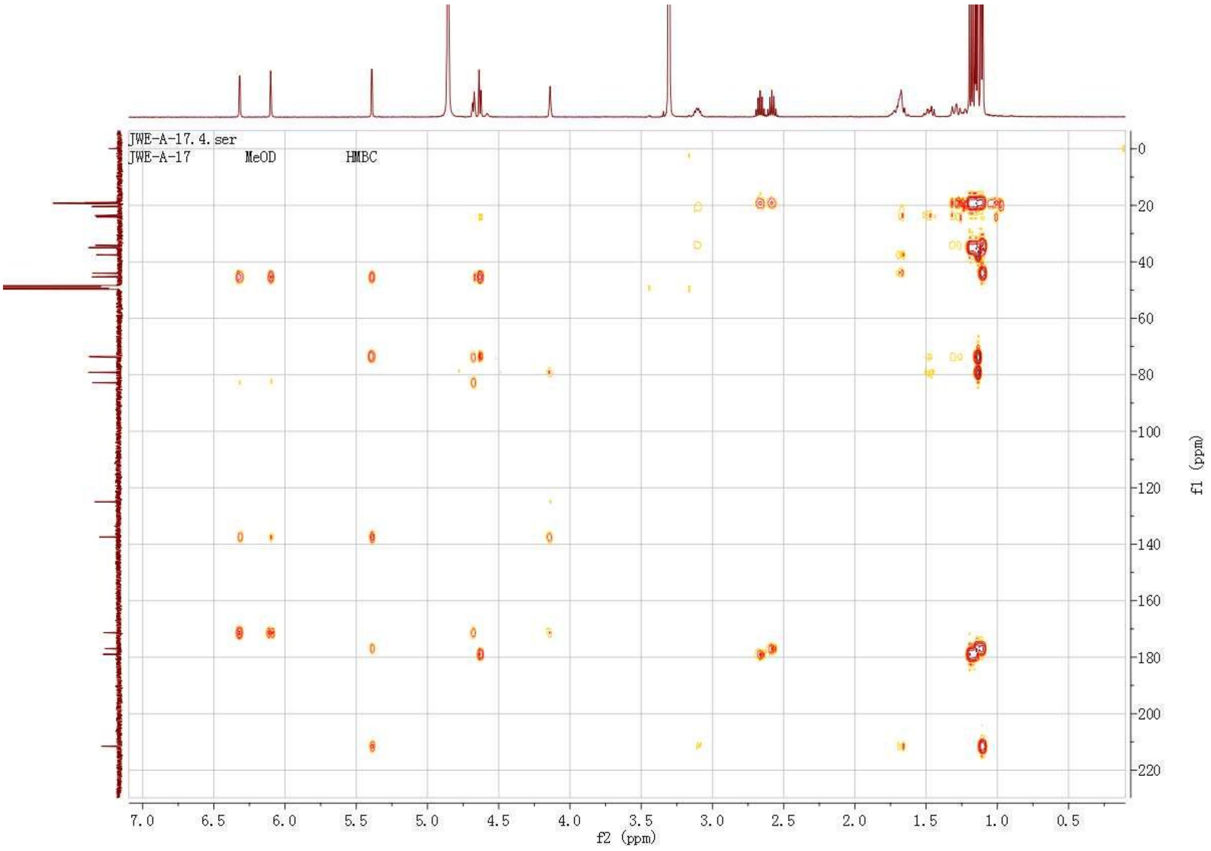

Fig. S1.5 HMBC spectrum (500 MHz) of compound **1** in CD_3OD

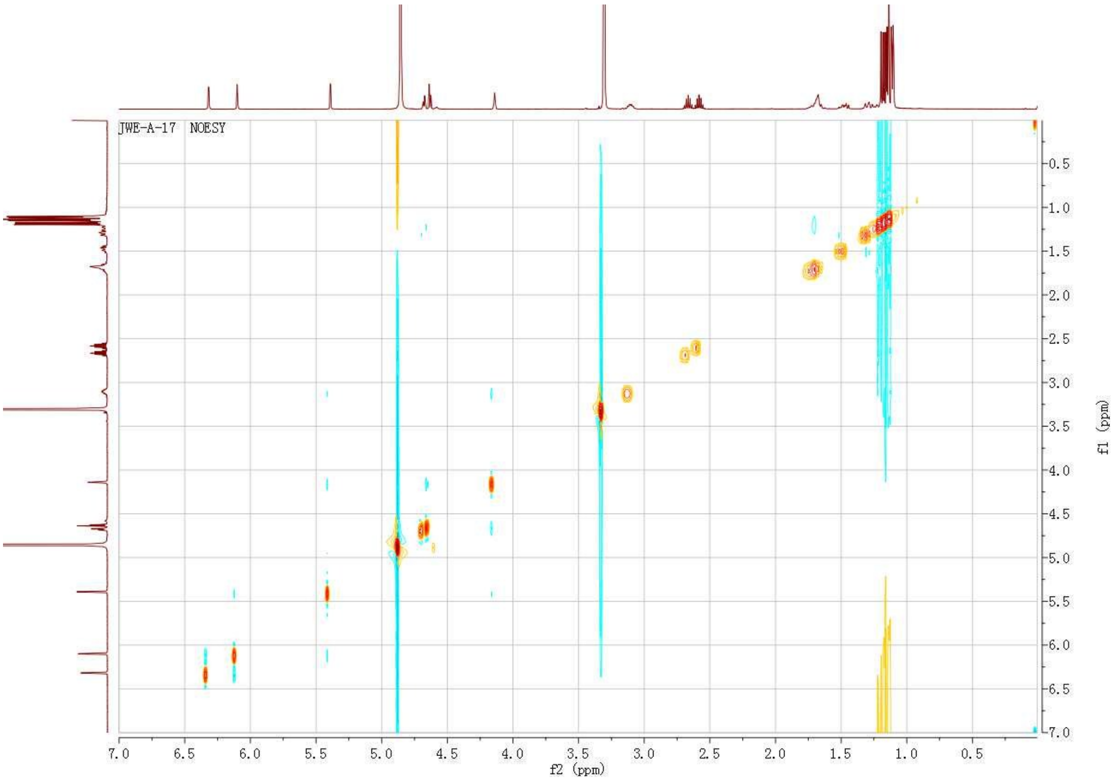

Fig. S1.6 ROESY spectrum (500 MHz) of compound **1** in CD_3OD

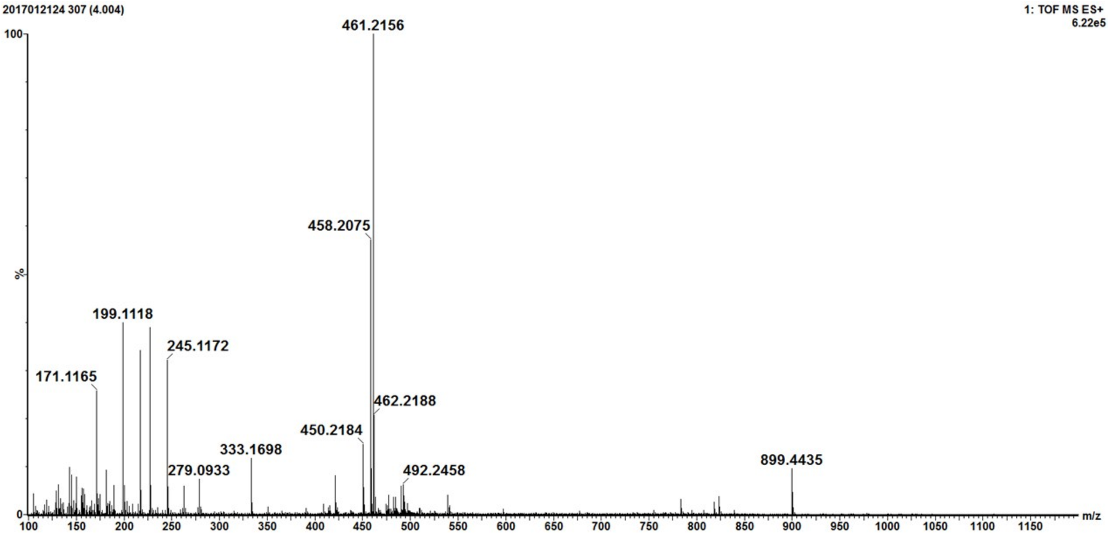

Fig. S1.7 HRESIMS spectrum of compound **1**

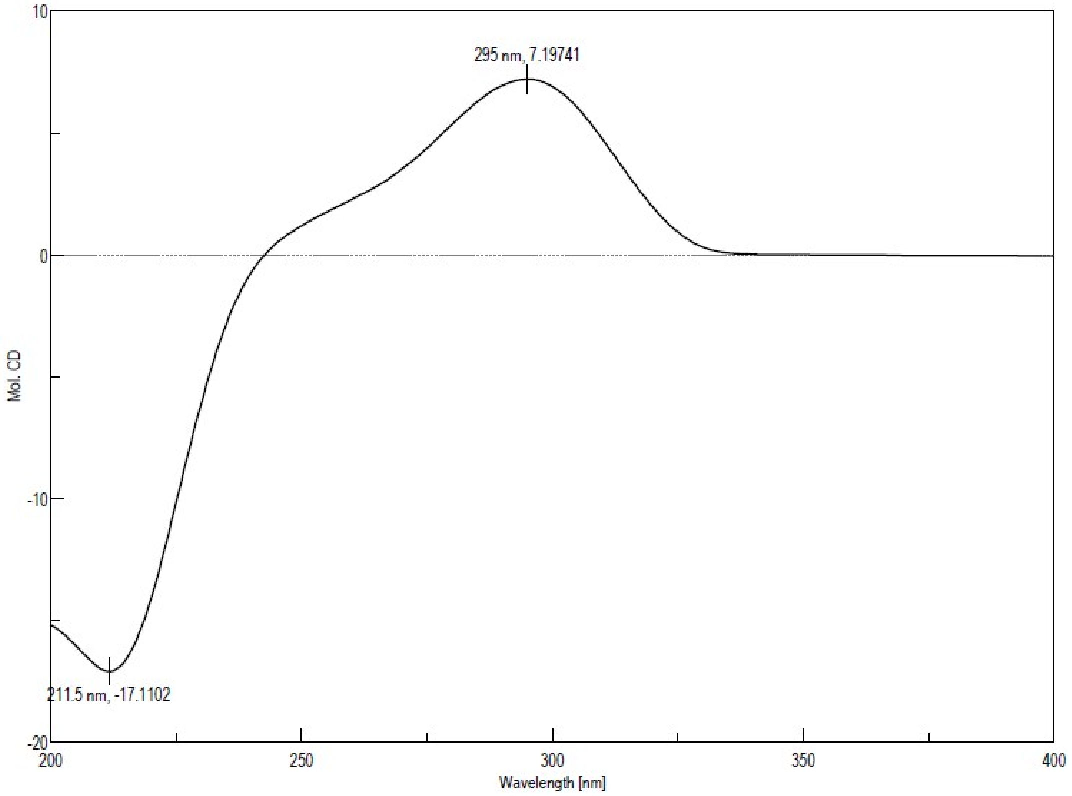

Fig. S1.8 CD spectrum of compound **1**

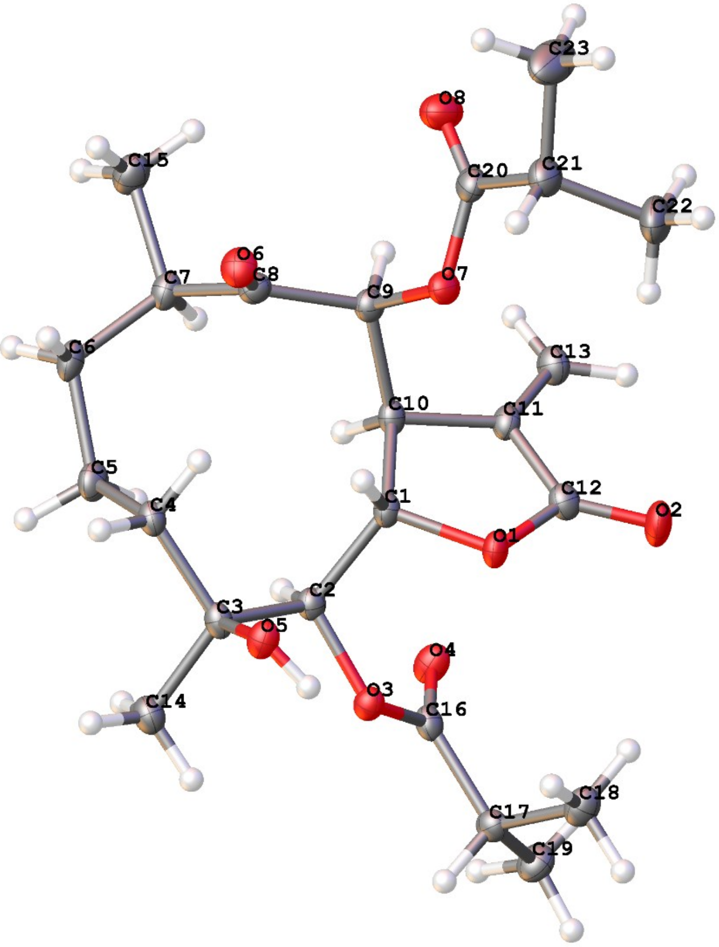

Fig. S1.9 X-ray ORTEP drawing of **1**

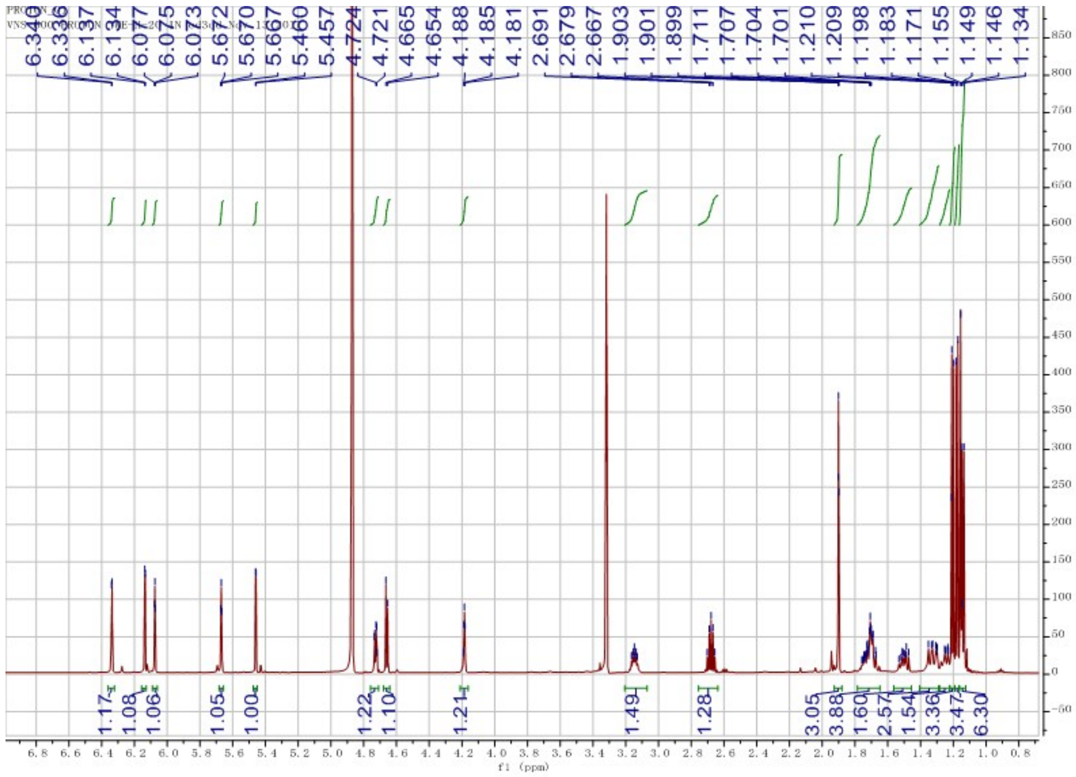

Fig. S2.1 ^{1}H NMR spectrum (600 MHz) of compound **2** in CD_3OD

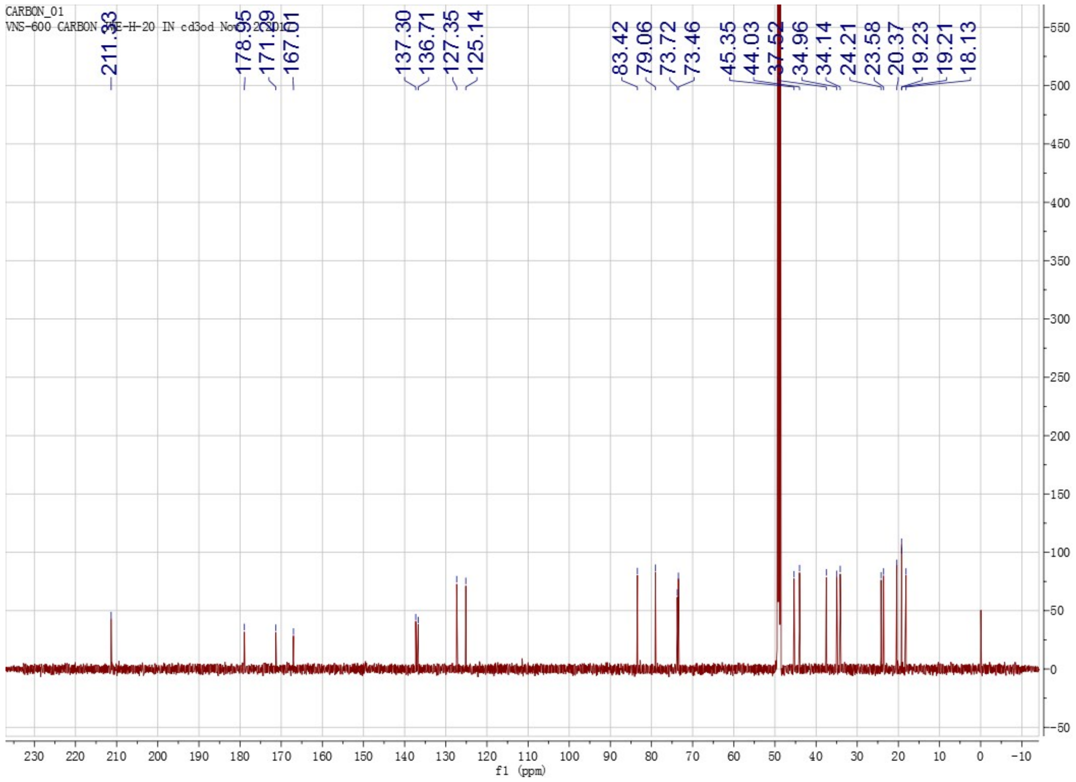

Fig. S2.2 ^{13}C NMR spectrum (150 MHz) of compound **2** in CD_3OD

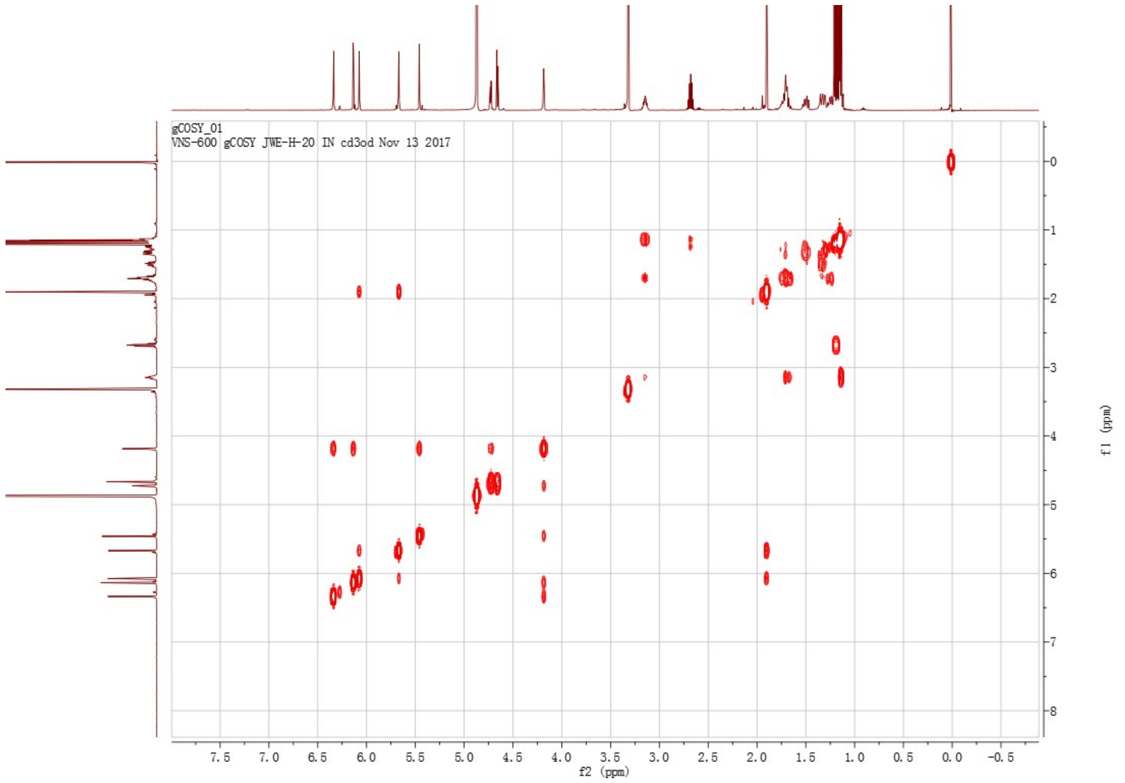

Fig. S2.3 ^{1}H-^{1}H COSY spectrum (600 MHz) of compound **2** in CD_3OD

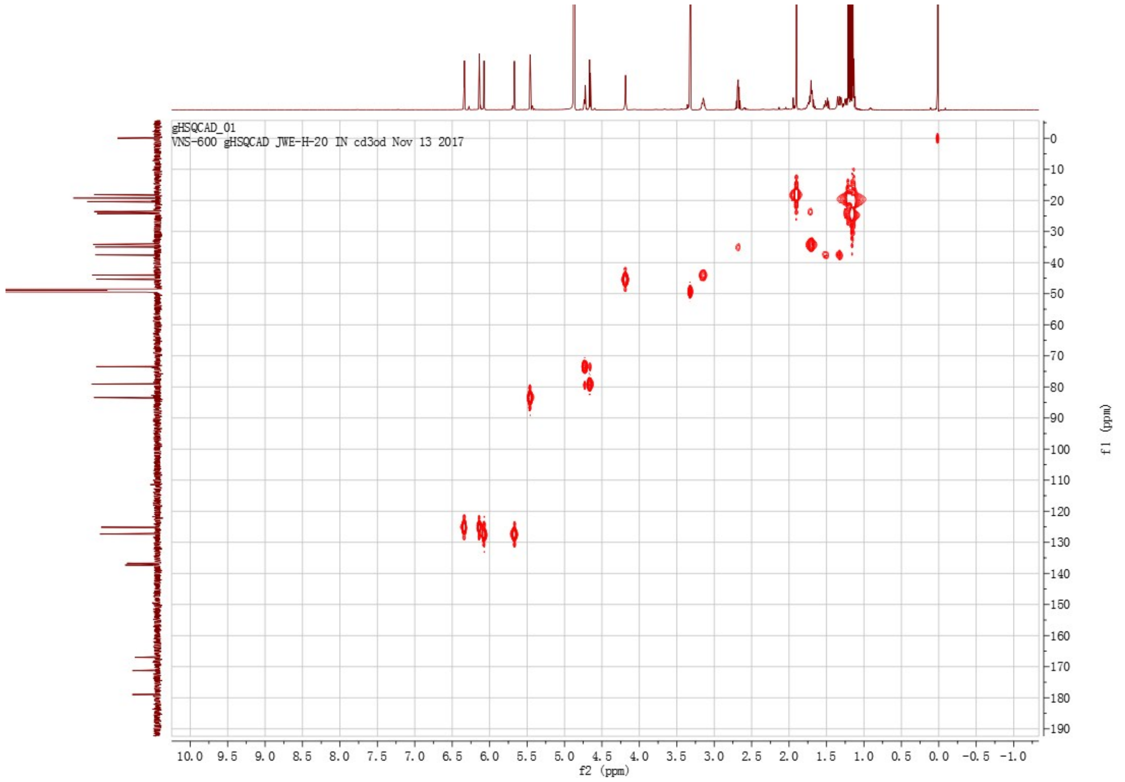

Fig. S2.4 HSQC spectrum (600 MHz) of compound **2** in CD_3OD

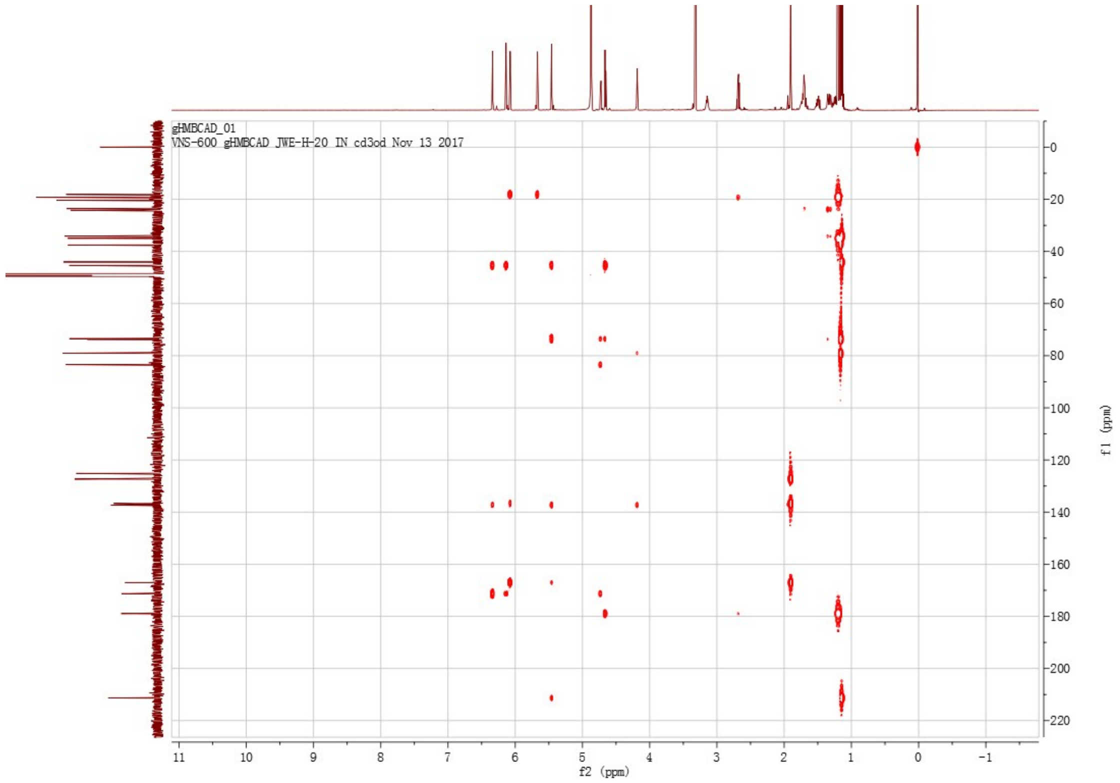

Fig. S2.5 HMBC spectrum (600 MHz) of compound **2** in CD_3OD

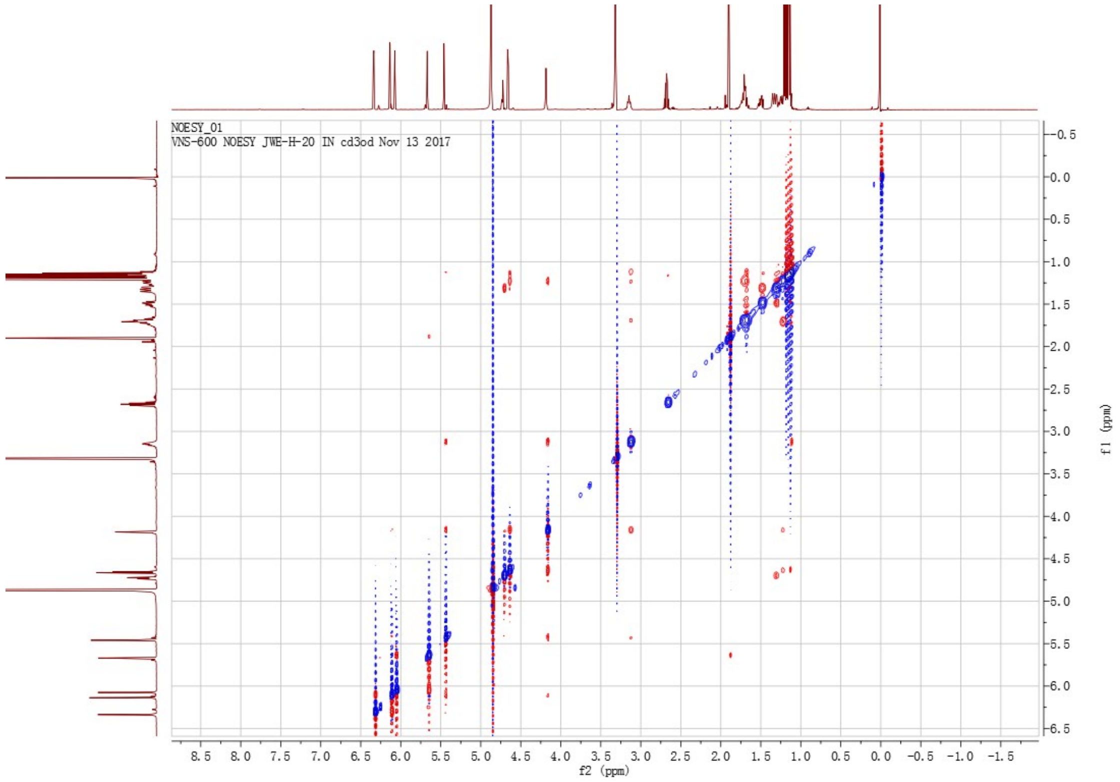

Fig. S2.6 NOESY spectrum (600 MHz) of compound **2** in CD_3OD

Auto Print Report 1 2006-11-30 00:49:12

Data Set: Storage 004614 - RawData - D:\2018\JCB-H-20.spc

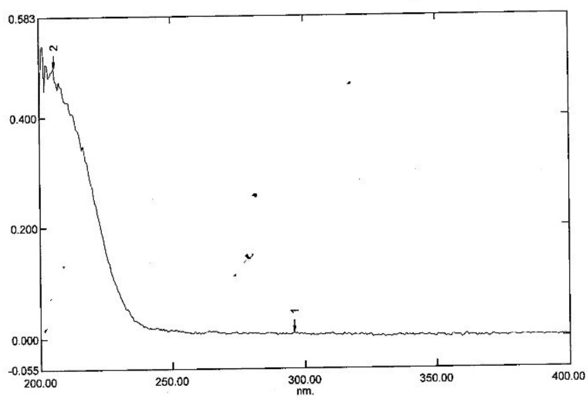

No.	P/V	Wavelength	Abs.	Description
1	●	296.00	0.009	
2	●	205.50	0.490	

样品溶于1.20ml MeOH

旋光：-0.002°

UV: 吸取0.5ml→3.0ml

Measurement Properties
Wavelength Range (nm.): 200.00 to 400.00
Scan Speed: Medium
Sampling Interval: 0.5
Auto Sampling Interval: Disabled
Scan Mode: Single

Sample Preparation Properties
Weight:
Volume:

Page 1 / 1

Fig. S2.7 UV spectrum of compound **2**

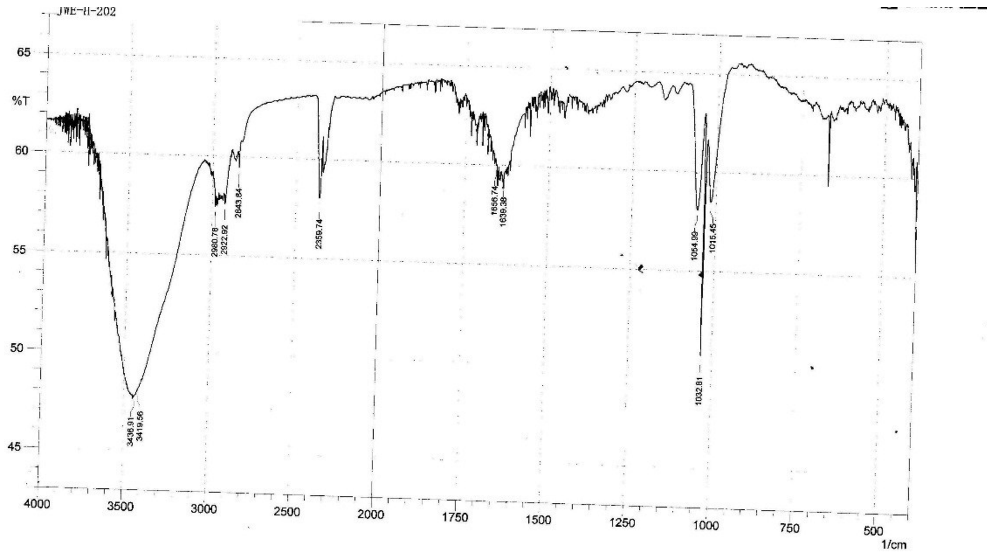

Date/Time; 2018-4-27 9:02:37

Fig. S2.8 IR spectrum of compound **2**

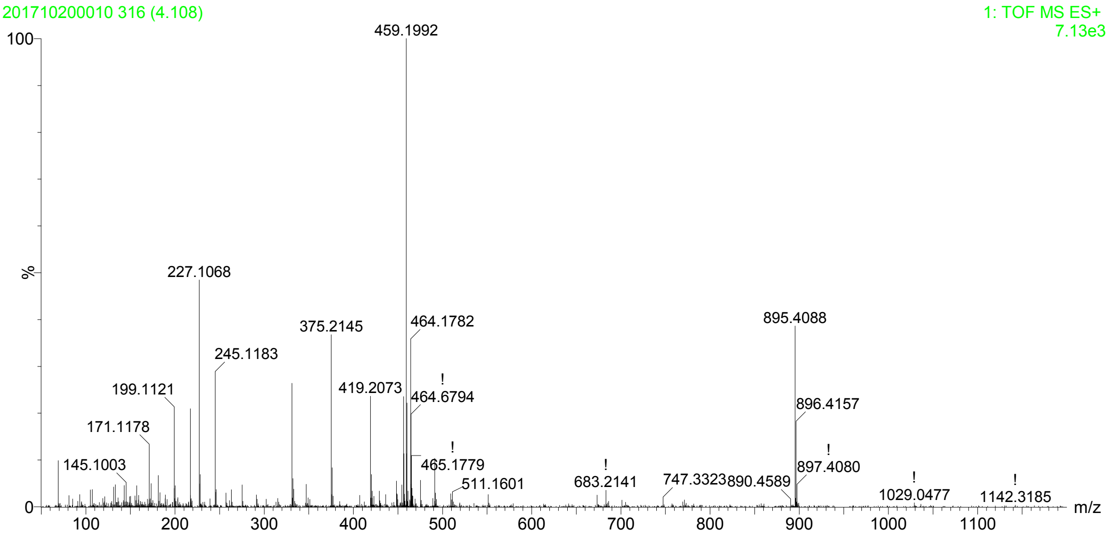

Fig. S2.9 HRESIMS spectrum of compound **2**

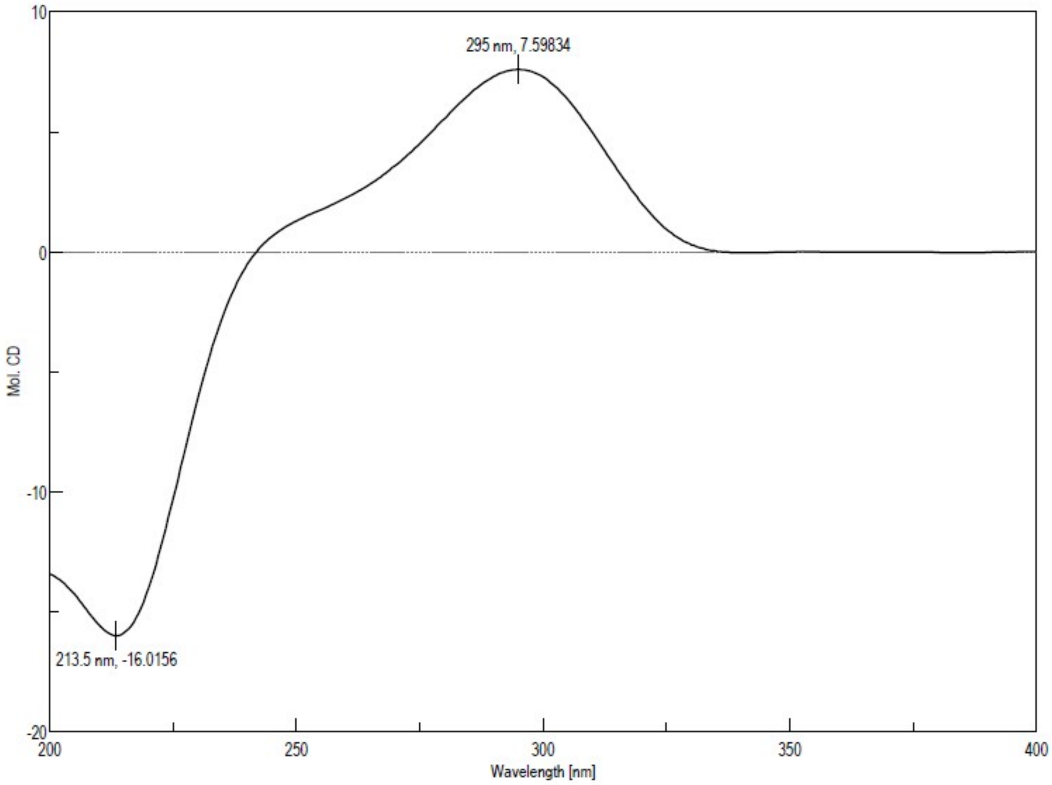

Fig. S2.10 CD spectrum of compound **2**

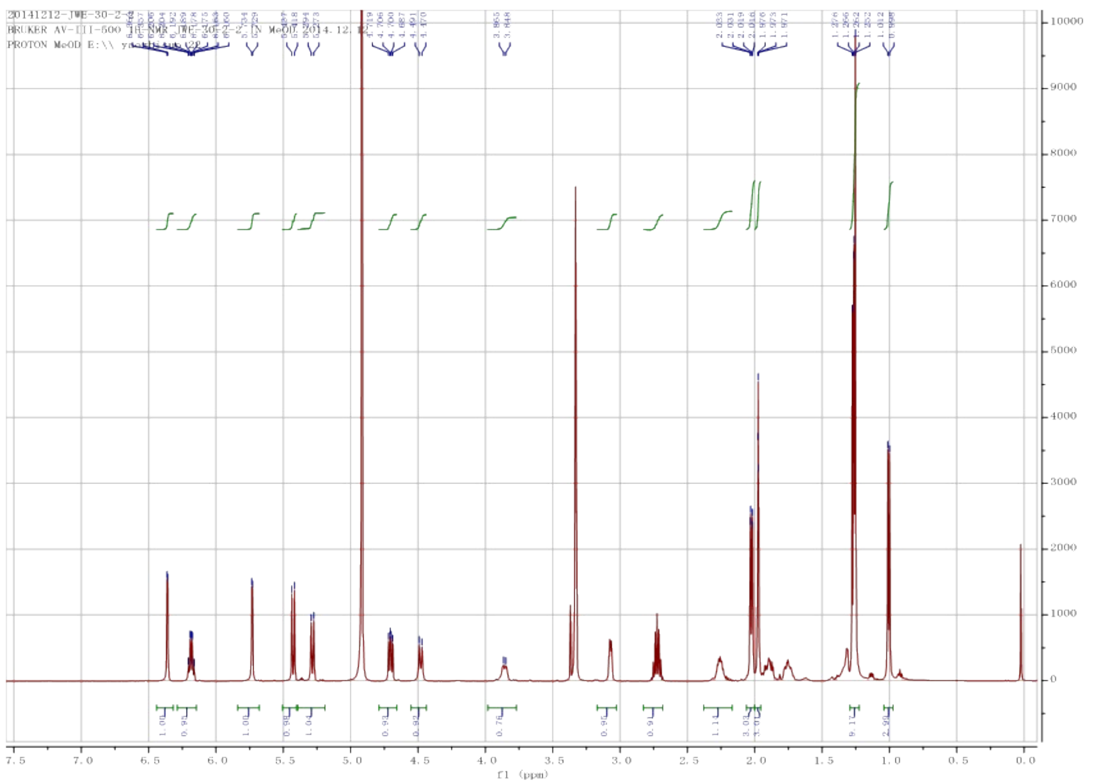

Fig. S3.1 ^{1}H NMR spectrum (600 MHz) of compound **3** in CD_3OD

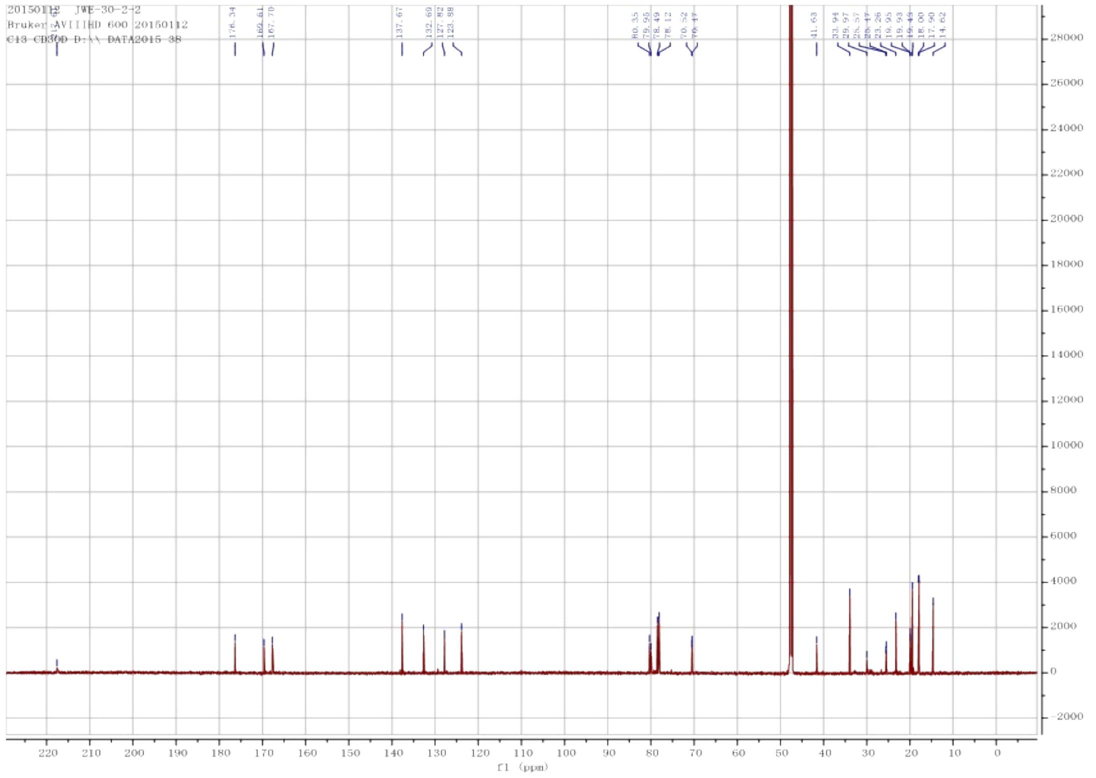

Fig. S3.2 ^{13}C NMR spectrum (150 MHz) of compound **3** in CD_3OD

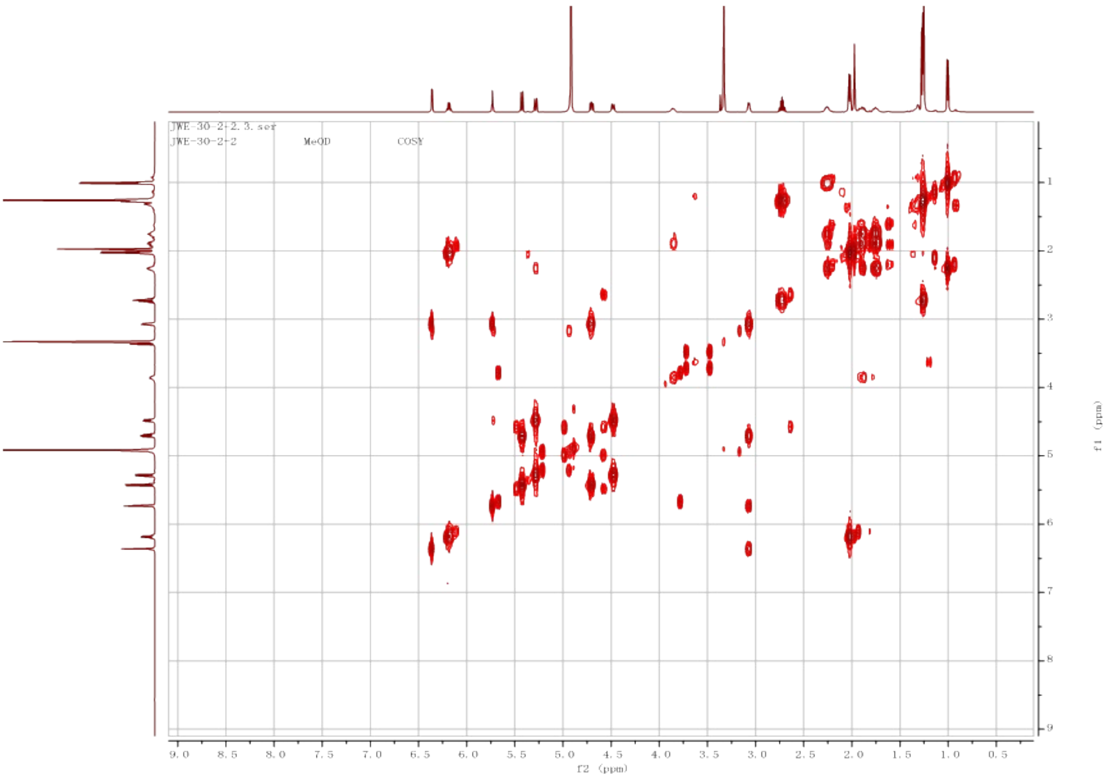

Fig. S3.3 ^{1}H-^{1}H COSY spectrum (600 MHz) of compound **3** in CD_3OD

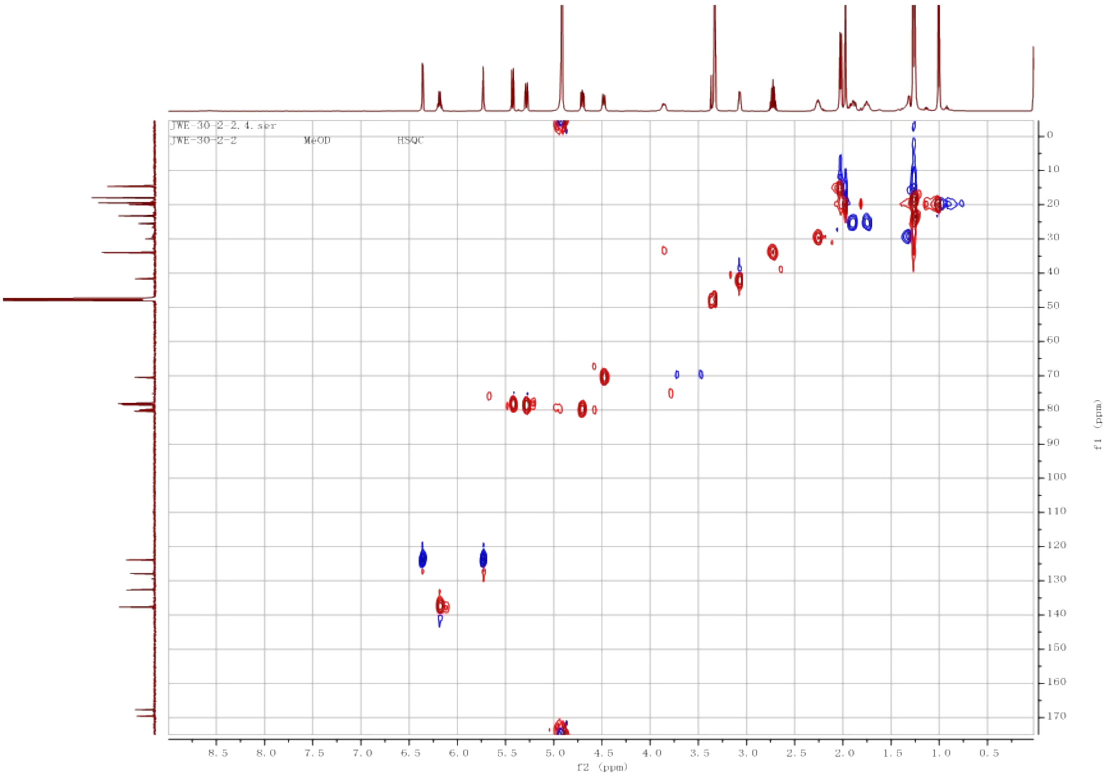

Fig. S3.4 HSQC spectrum (600 MHz) of compound **3** in CD_3OD

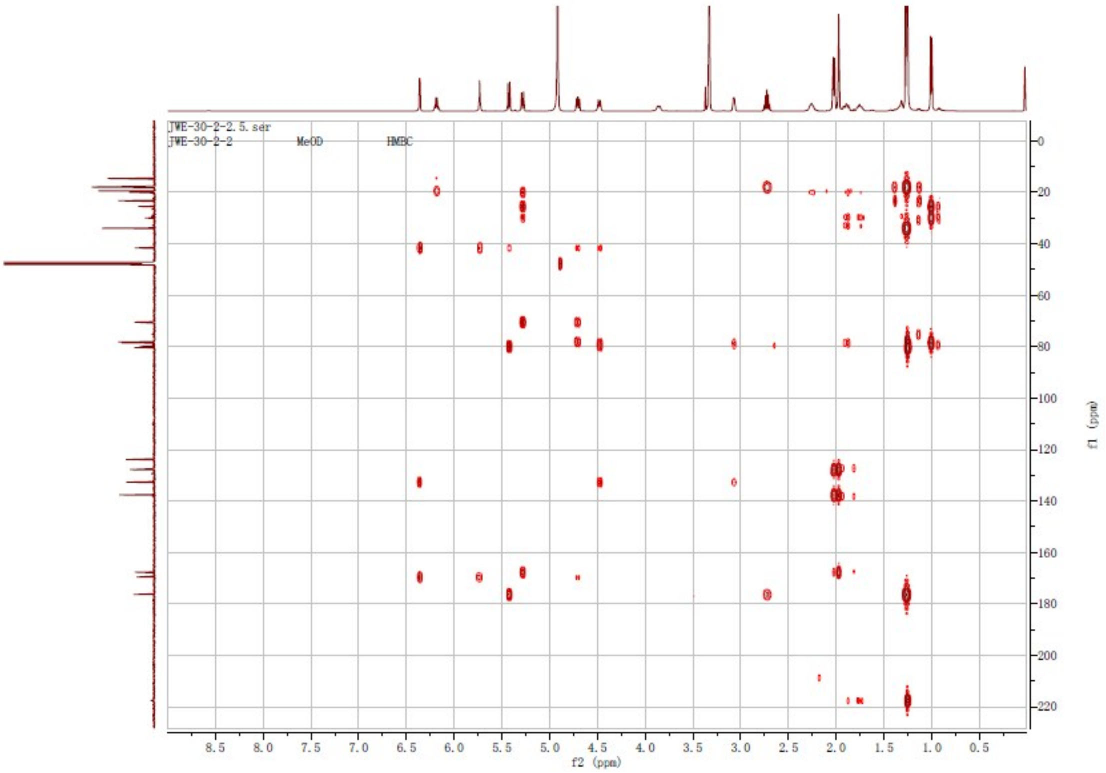

Fig. S3.5 HMBC spectrum (600 MHz) of compound **3** in CD_3OD

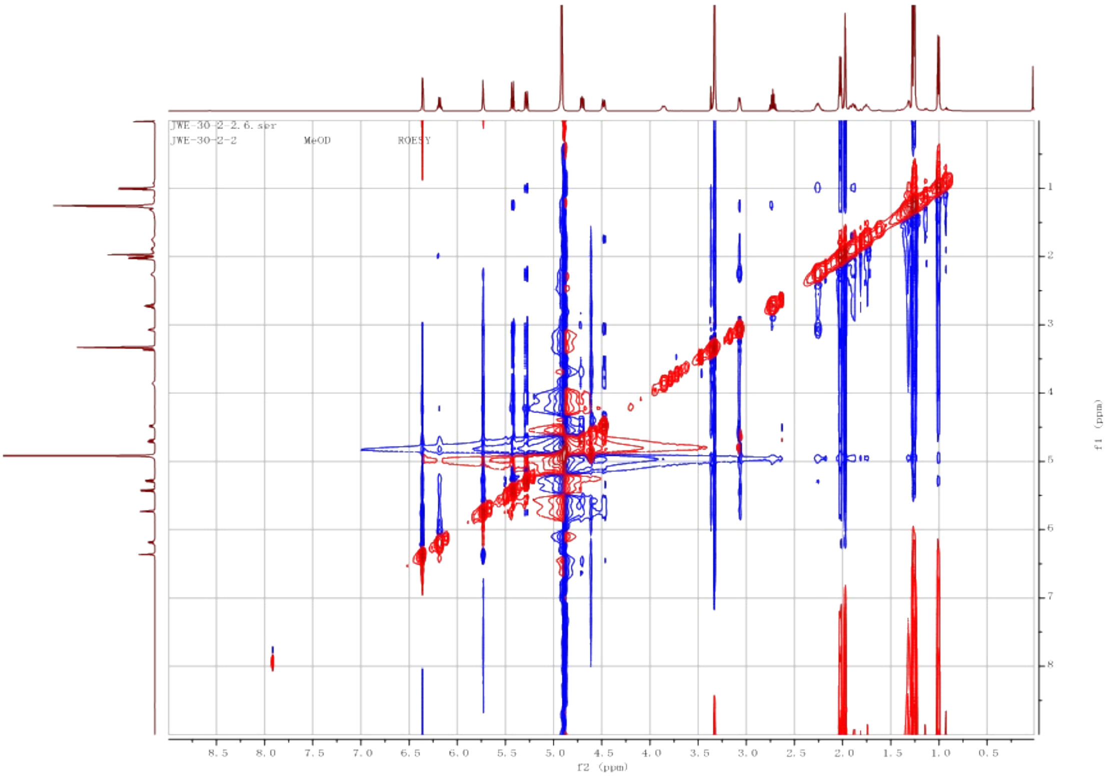

Fig. S3.6 ROESY spectrum (600 MHz) of compound **3** in CD_3OD

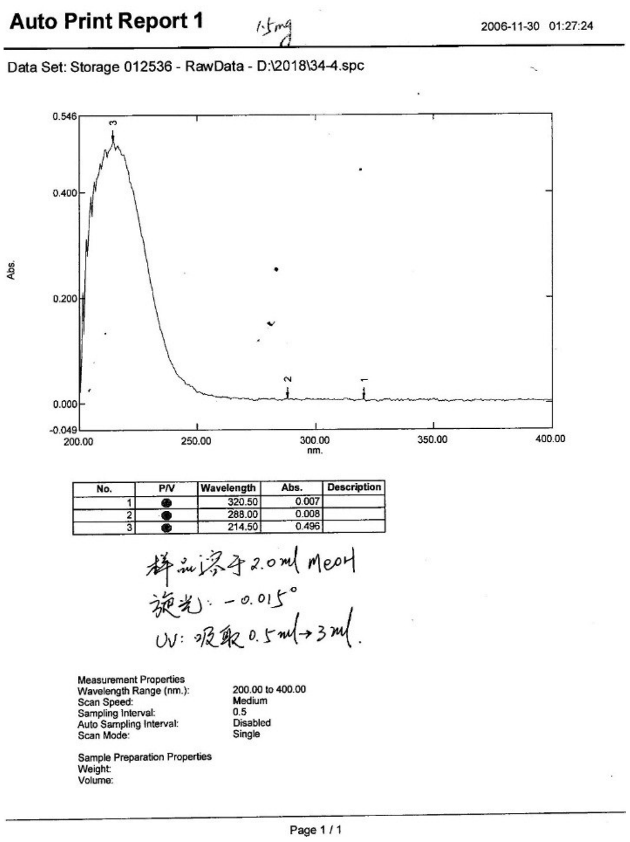

Fig. S3.7 UV spectrum of compound **3**

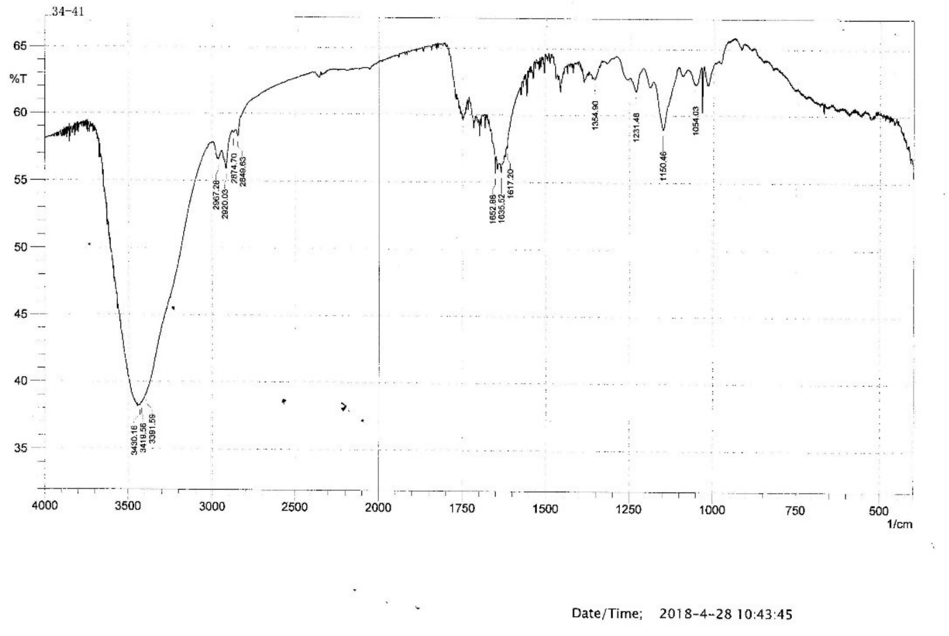

Fig. S3.8 IR spectrum of compound **3**

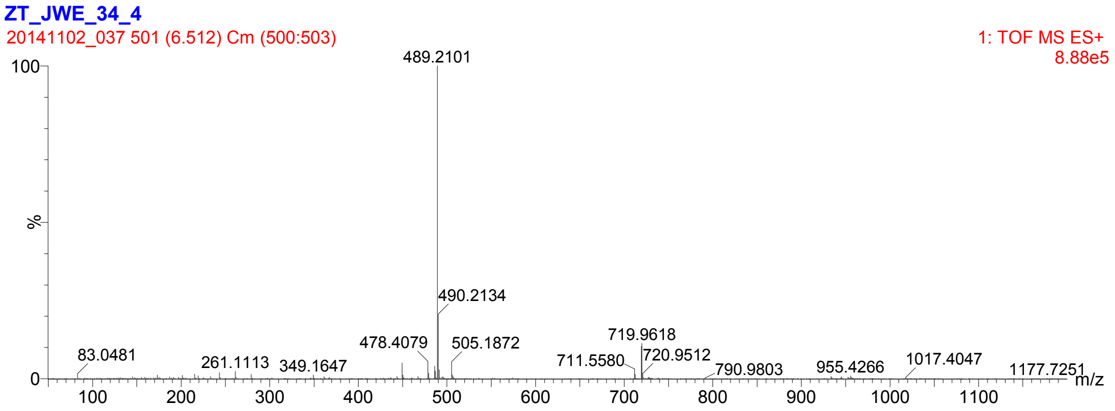

Fig. S3.9 HRESIMS spectrum of compound **3**

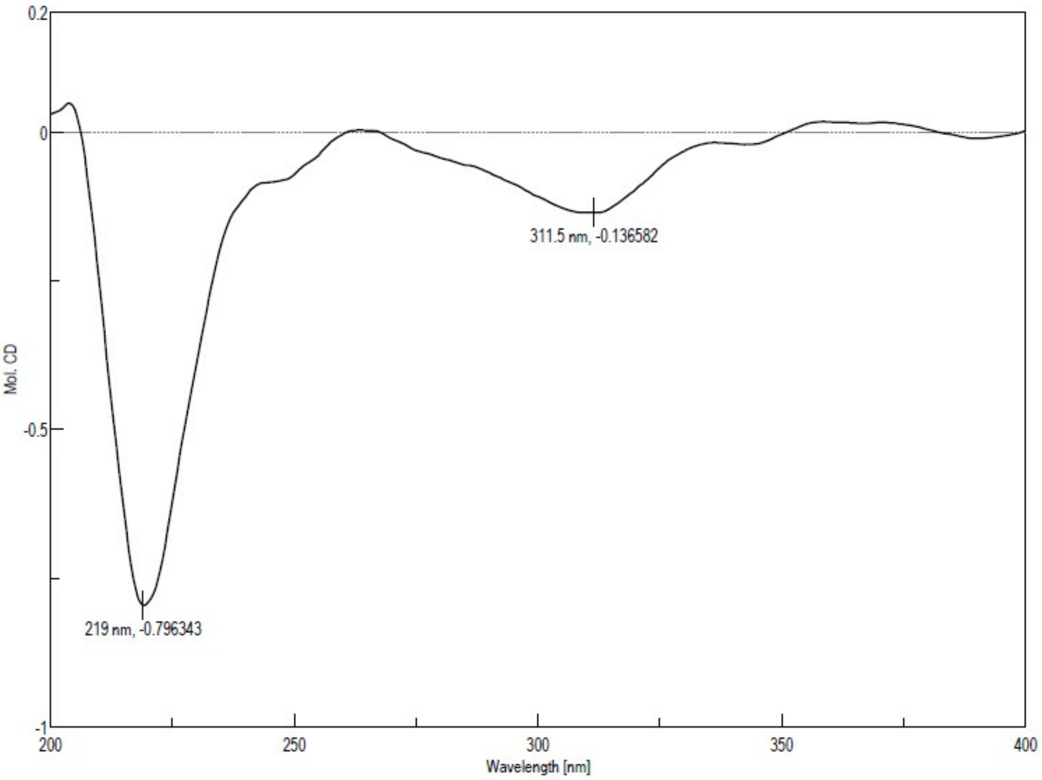

Fig. S3.10 CD spectrum of compound **3**

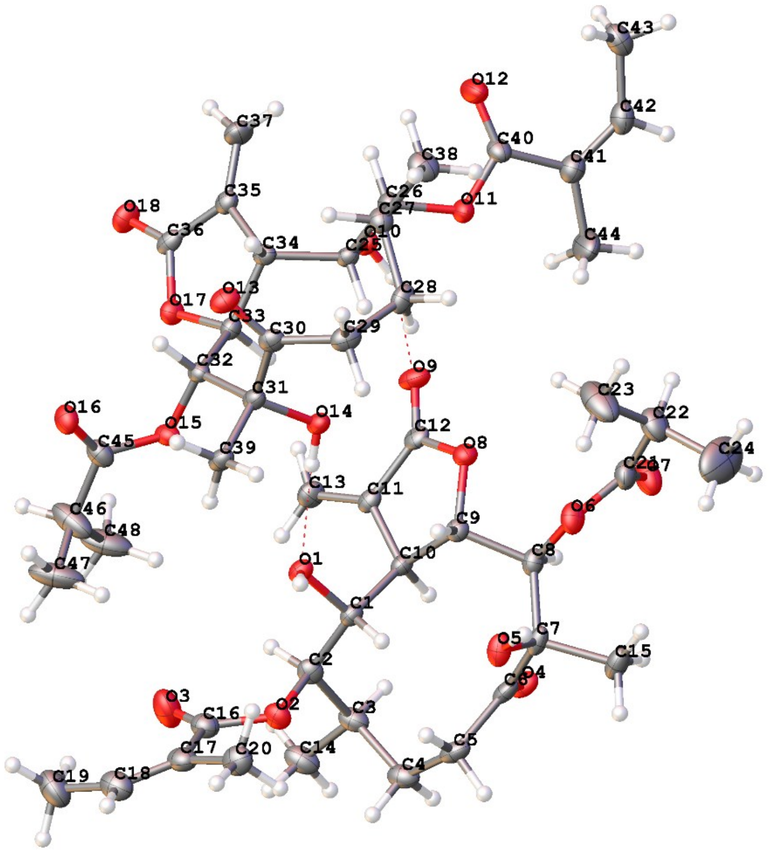

Fig. S3.11 X-ray ORTEP drawing of **3**

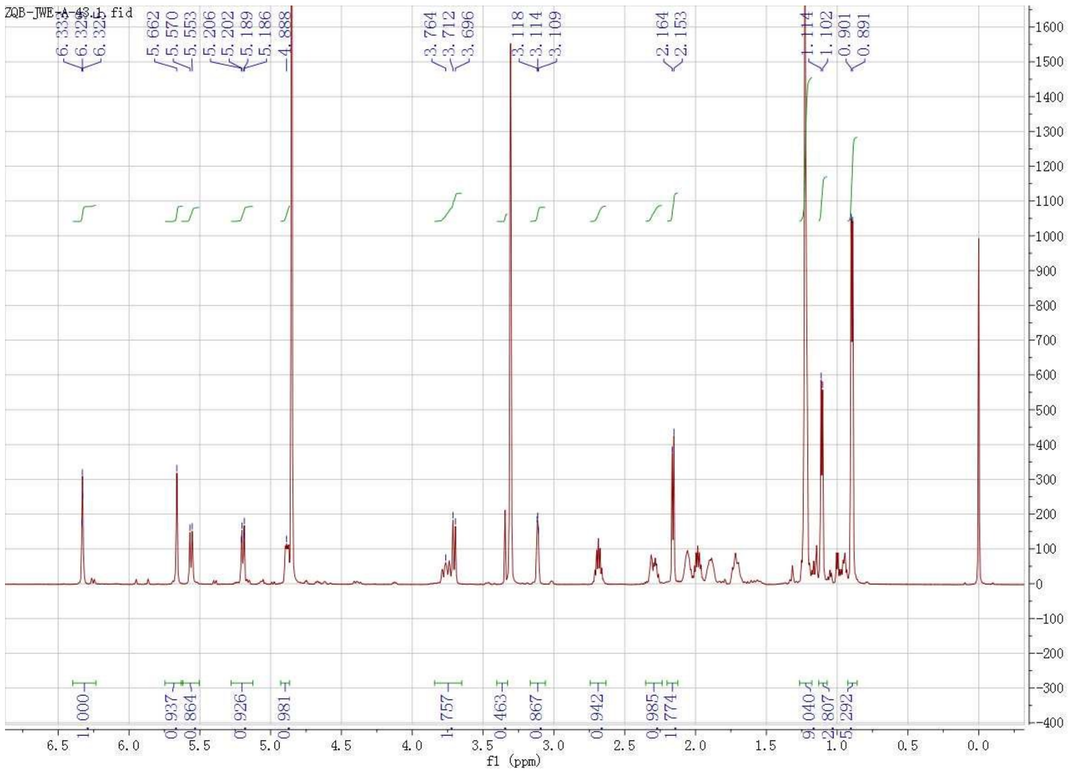

Fig. S4.1 ^{1}H NMR spectrum (500 MHz) of compound **4** in CD_3OD

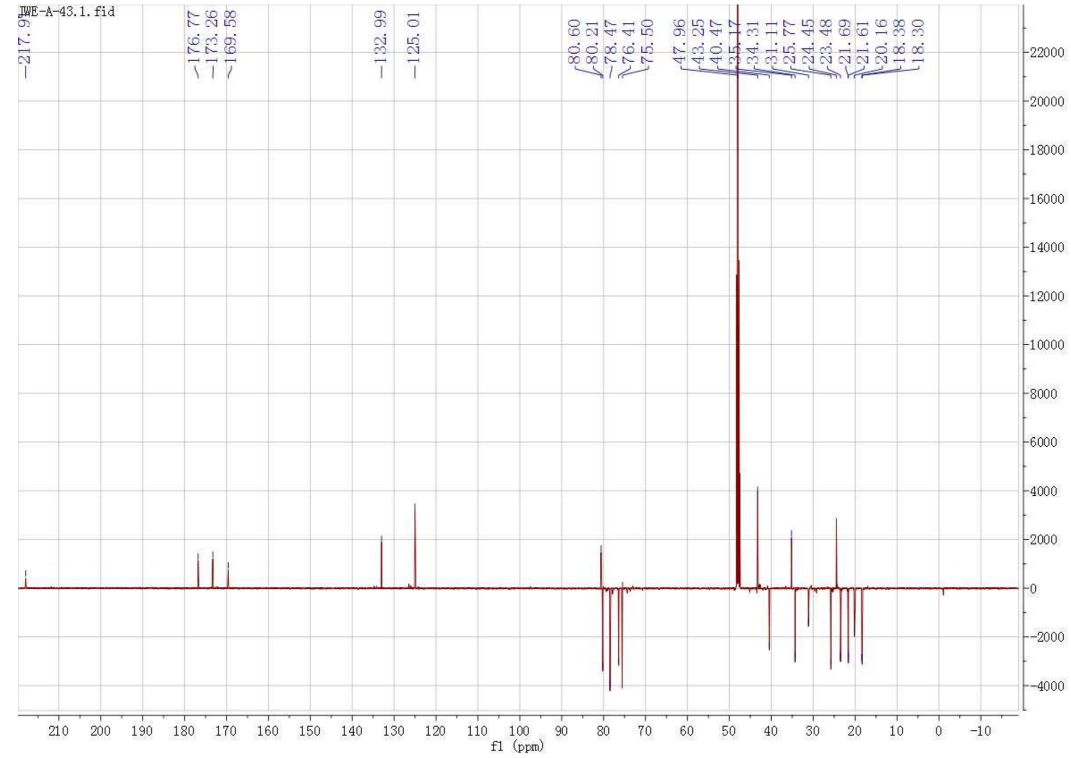

Fig. S4.2 ^{13}C NMR spectrum (125 MHz) of compound **4** in CD_3OD

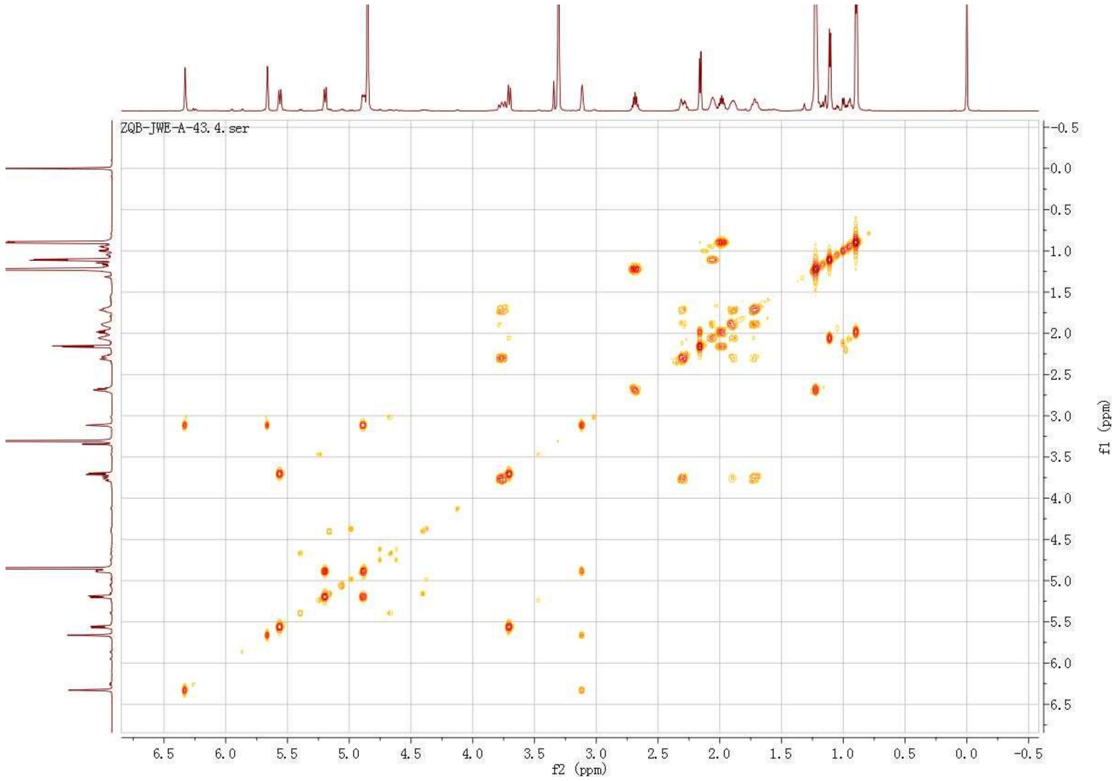

Fig. S4.3 ^{1}H-^{1}H COSY spectrum (500 MHz) of compound **4** in CD_3OD

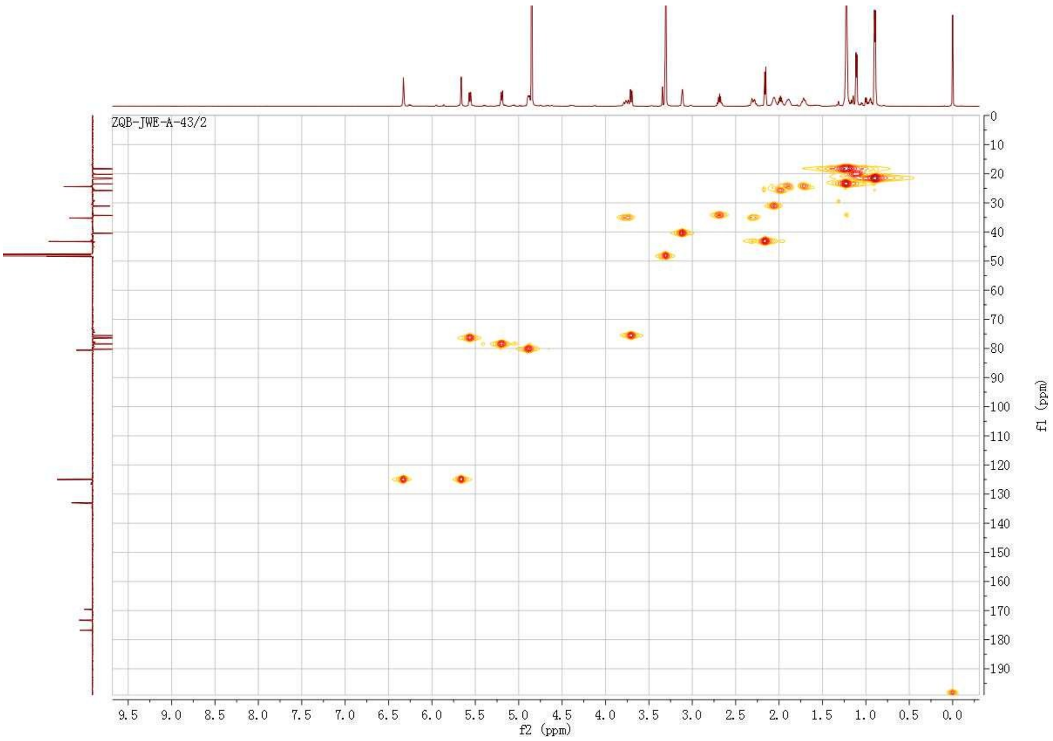

Fig. S4.4 HSQC spectrum (500 MHz) of compound **4** in CD_3OD

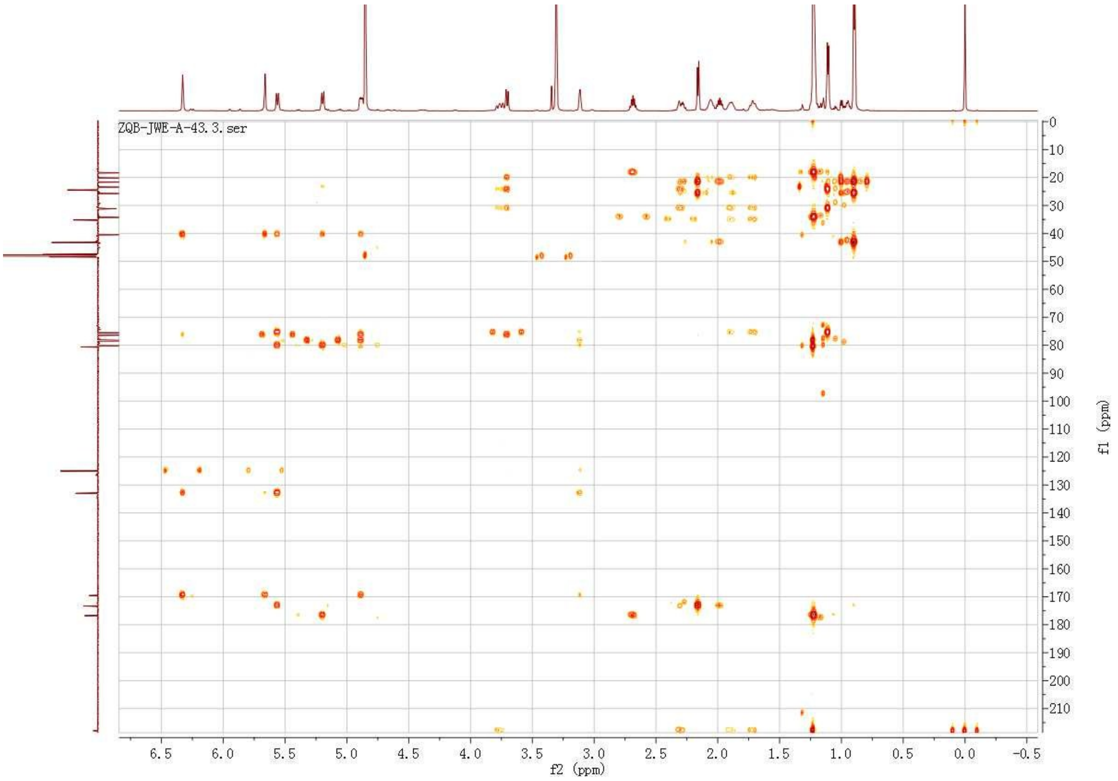

Fig. S4.5 HMBC spectrum (500 MHz) of compound **4** in CD_3OD

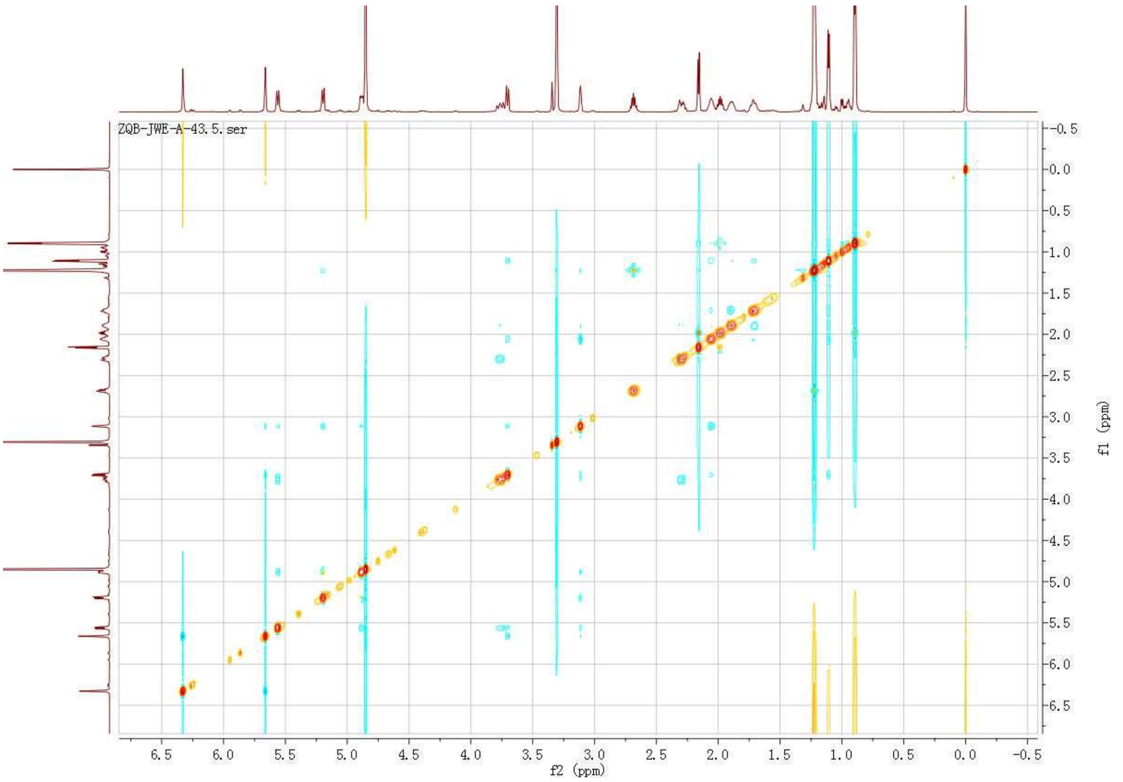

Fig. S4.6 ROESY spectrum (500 MHz) of compound **4** in CD_3OD

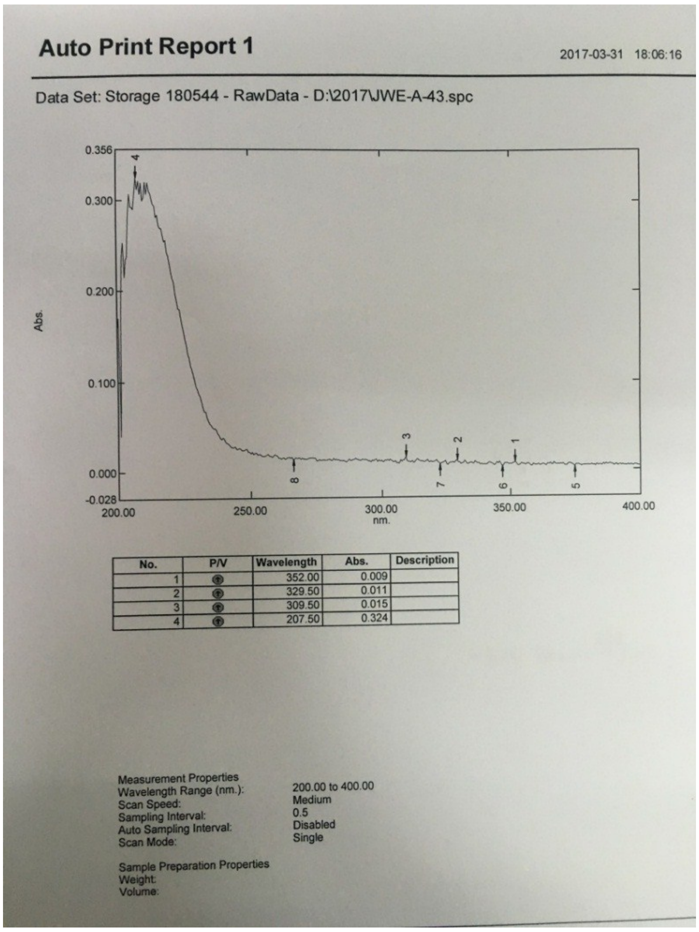

No.	P/V	Wavelength	Abs.	Description
1		352.00	0.009	
2		329.50	0.011	
3		309.50	0.015	
4		207.50	0.324	

Fig. S4.7 UV spectrum of compound **4**

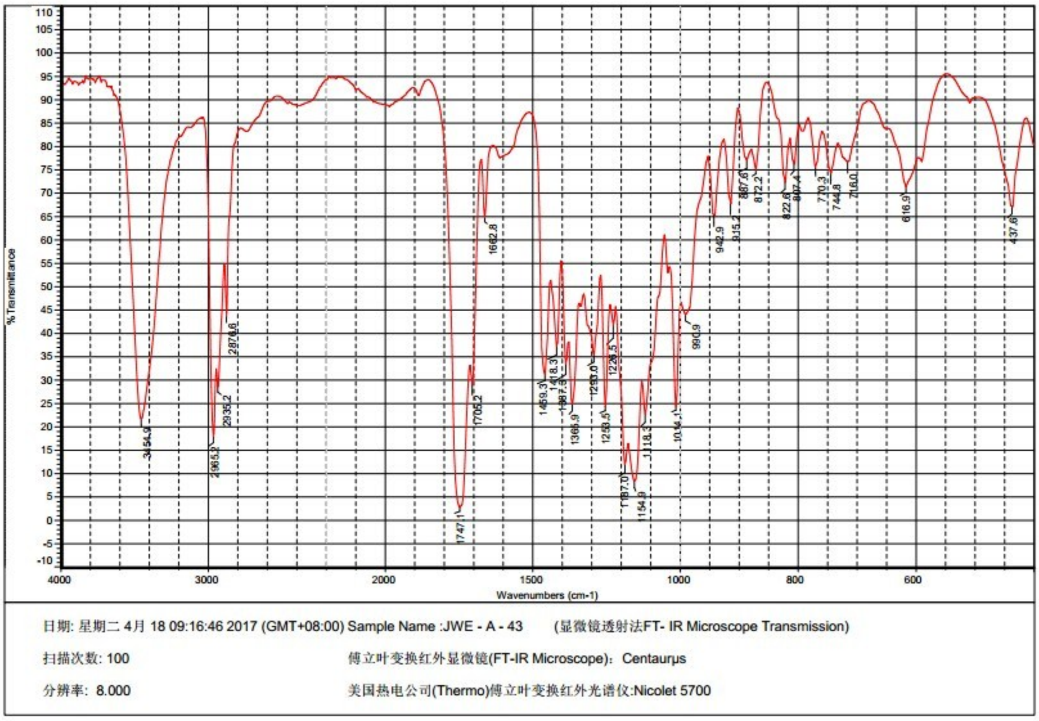

Fig. S4.8 IR spectrum of compound **4**

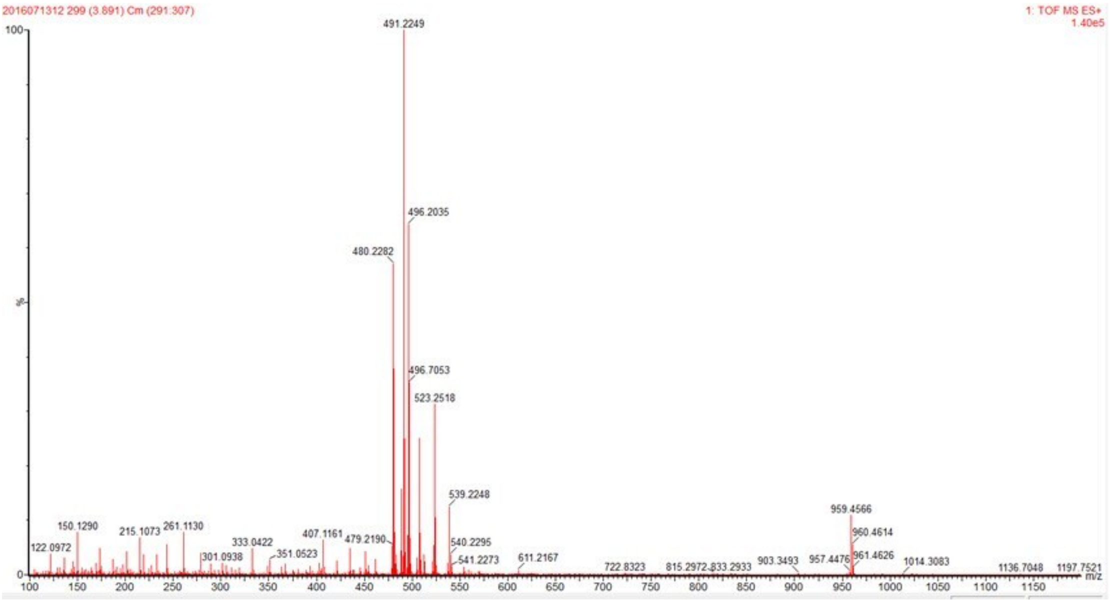

Fig. S4.9 HRESIMS spectrum of compound **4**

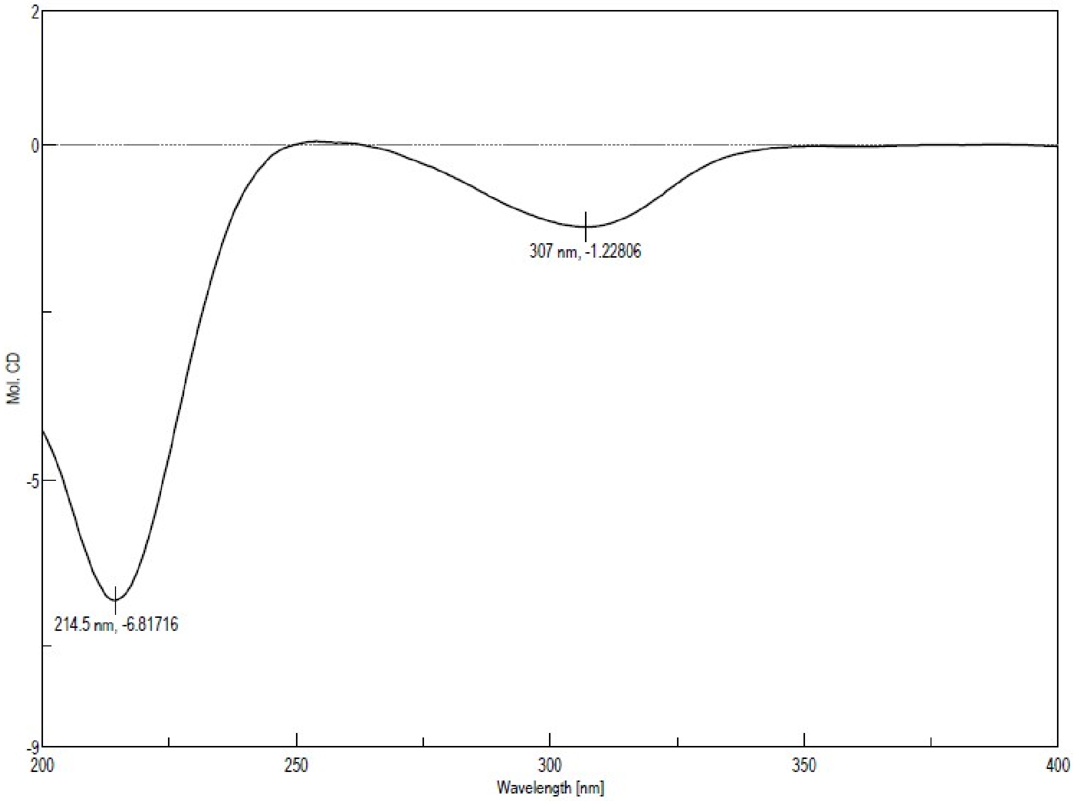

Fig. S4.10 CD spectrum of compound **4**

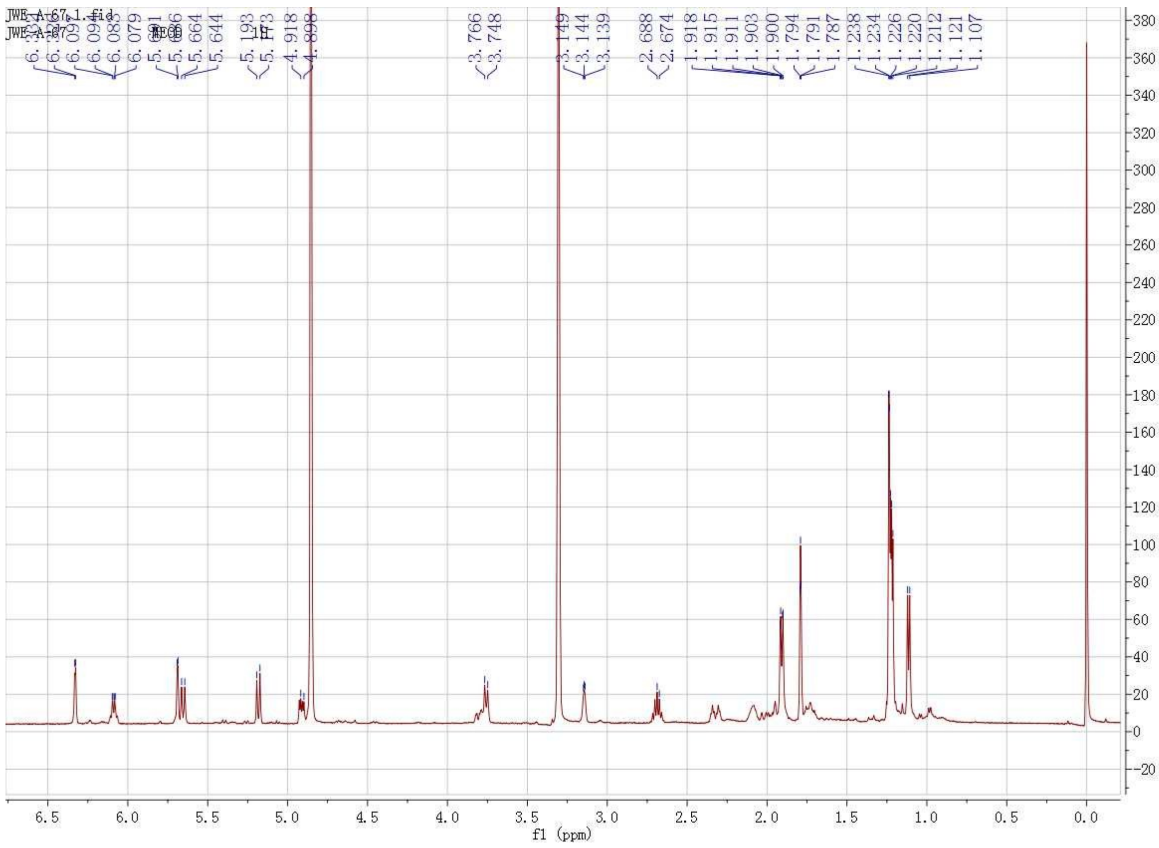

Fig. S5.1 ^{1}H NMR spectrum (500 MHz) of compound **5** in CD_3OD

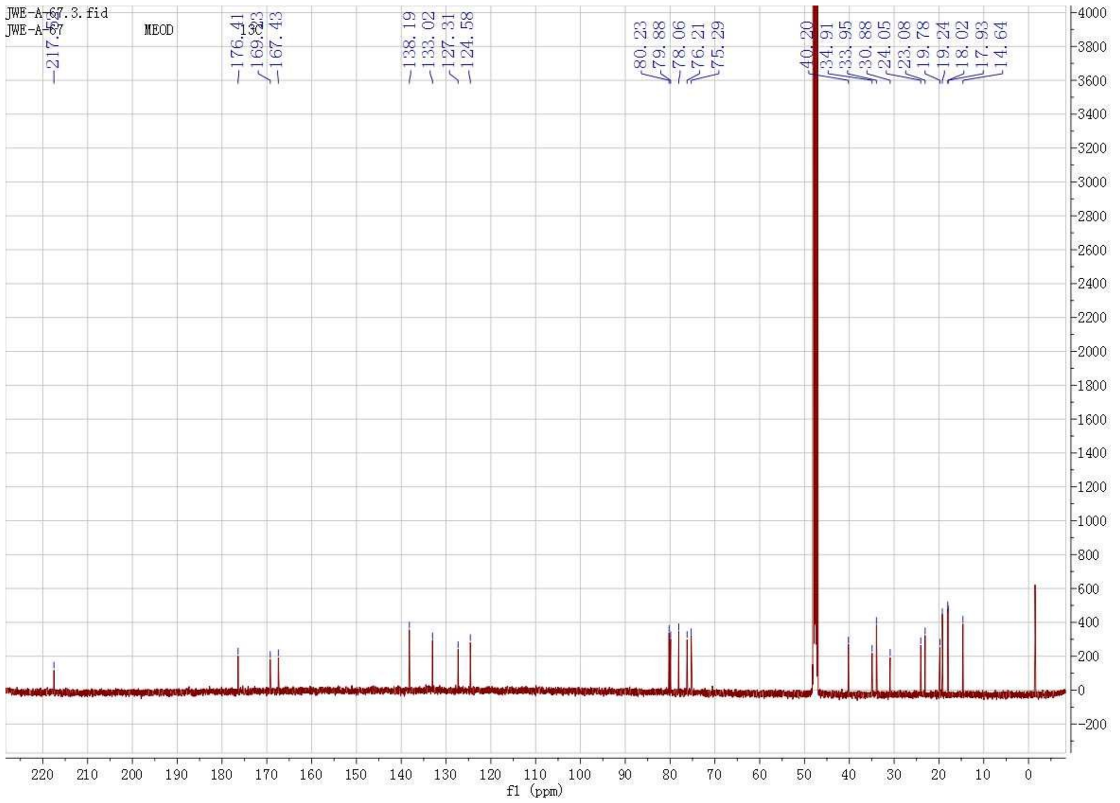

Fig. S5.2 ^{13}C NMR spectrum (500 MHz) of compound **5** in CD_3OD

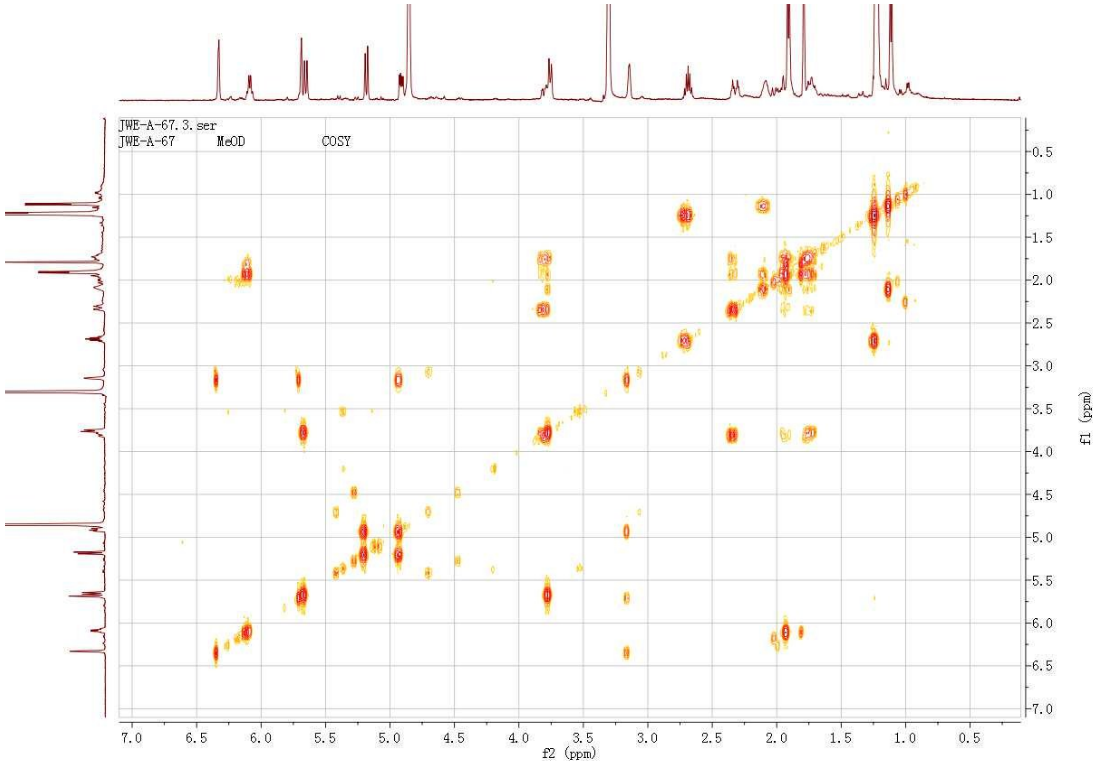

Fig. S5.3 ^{1}H-^{1}H COSY spectrum (500 MHz) of compound **5** in CD_3OD

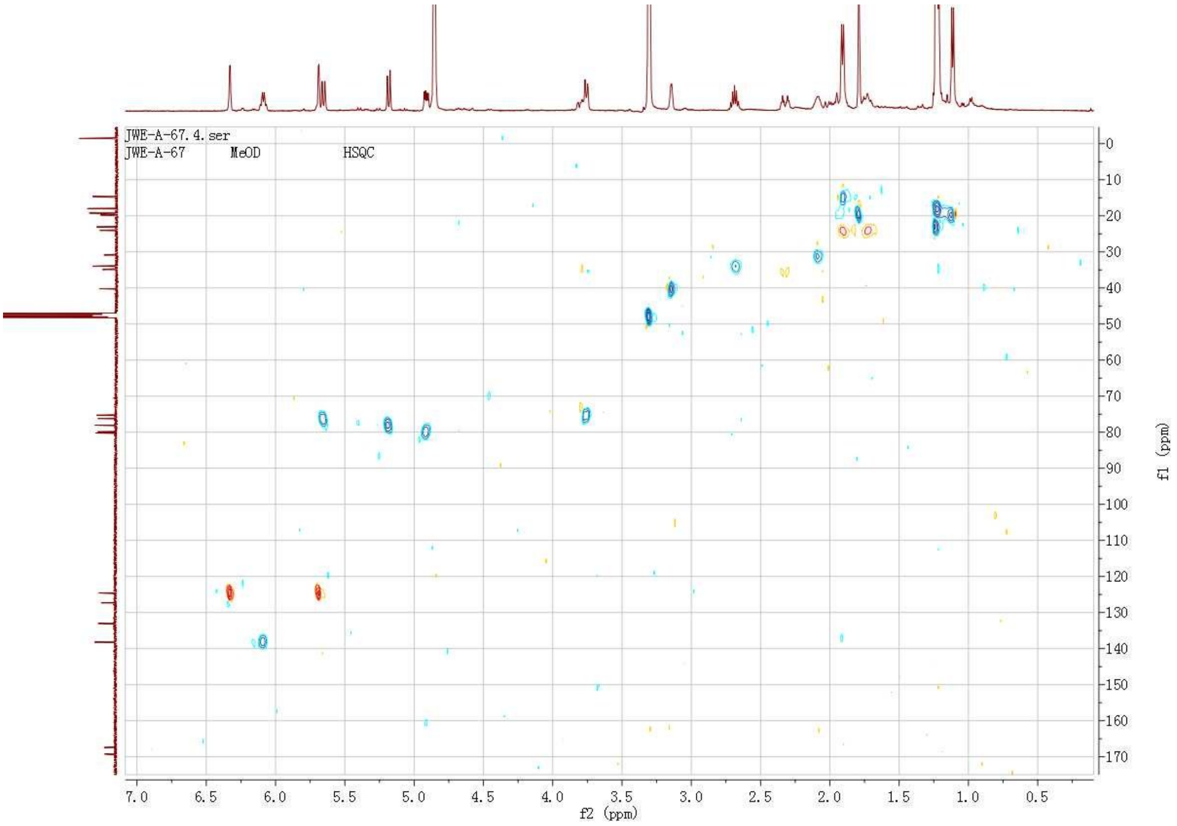

Fig. S5.4 HSQC spectrum (500 MHz) of compound **5** in CD_3OD

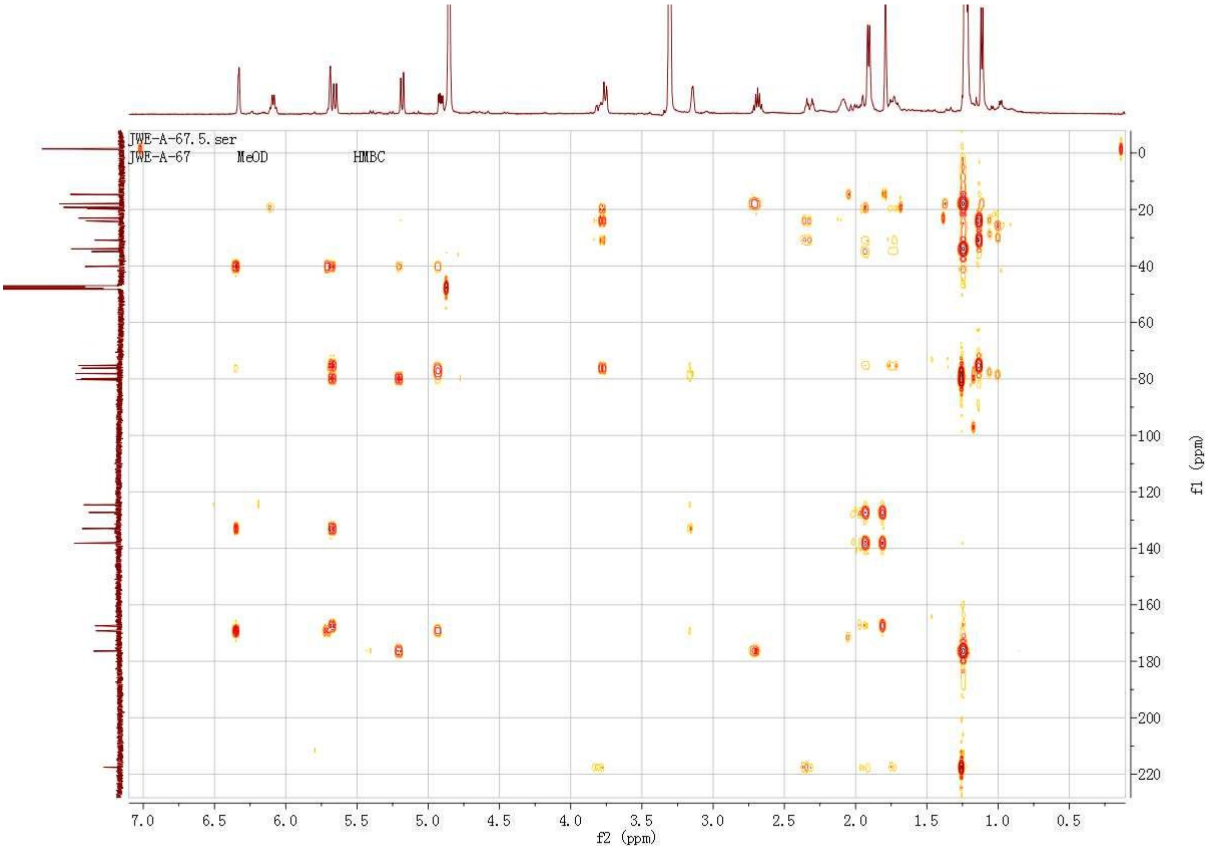

Fig. S5.5 HMBC spectrum (500 MHz) of compound **5** in CD_3OD

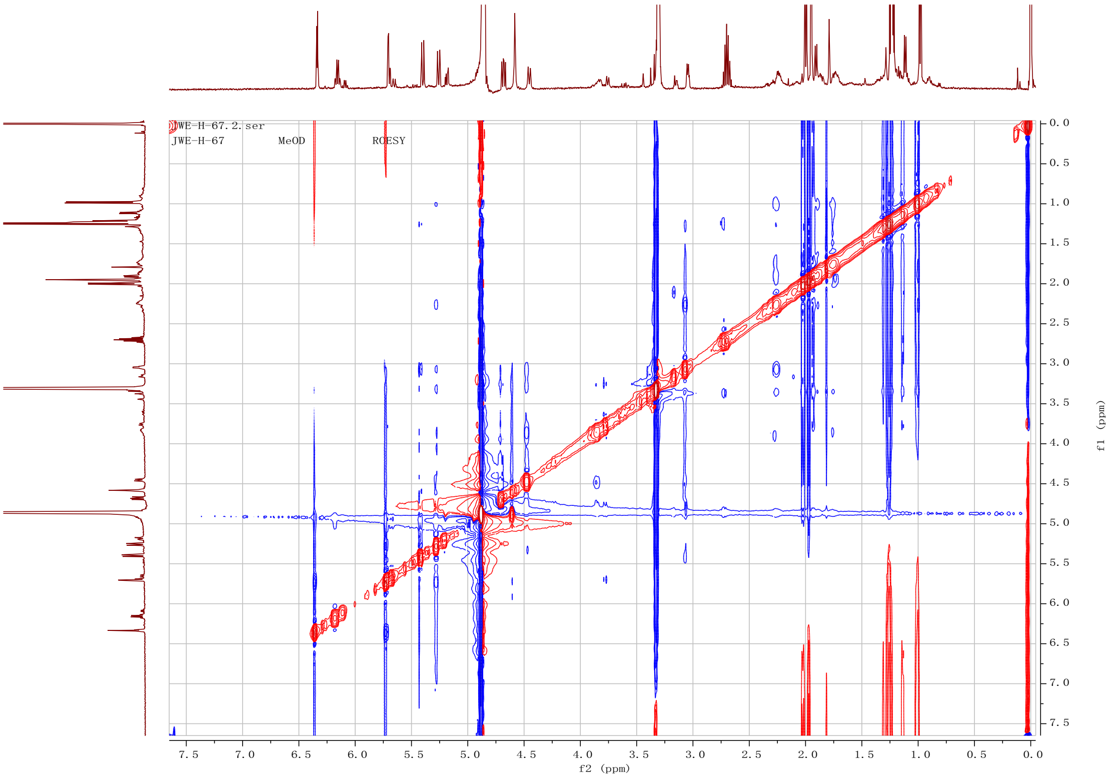

Fig. S5.6 ROESY spectrum (500 MHz) of compound **5** in CD_3OD

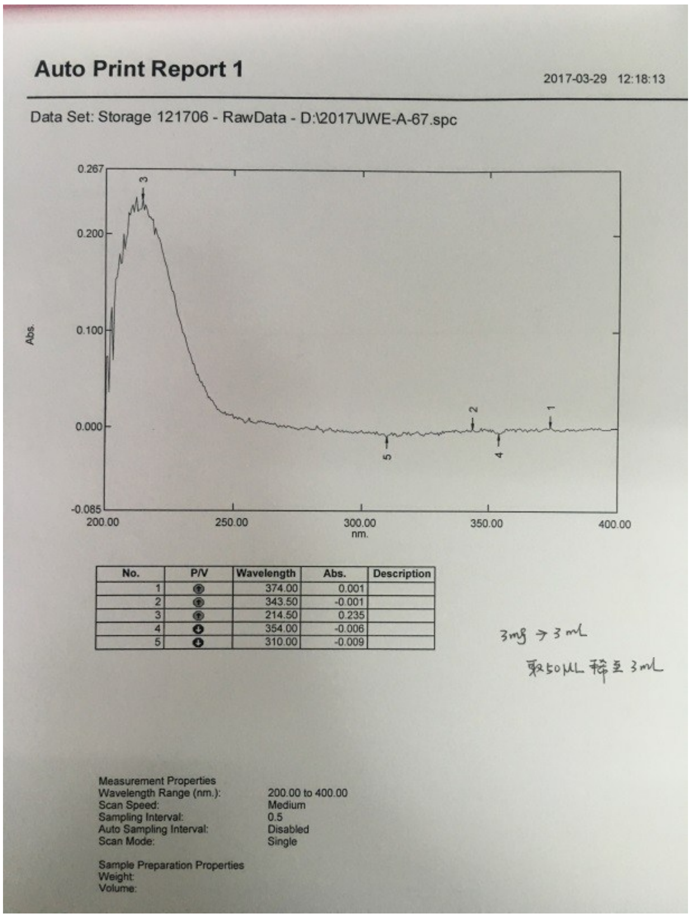

No.	P/V	Wavelength	Abs.	Description
1	⊕	374.00	0.001	
2	⊕	343.50	-0.001	
3	⊕	214.50	0.235	
4	⊖	354.00	-0.006	
5	⊖	310.00	-0.009	

Fig. S5.7 UV spectrum of compound **5**

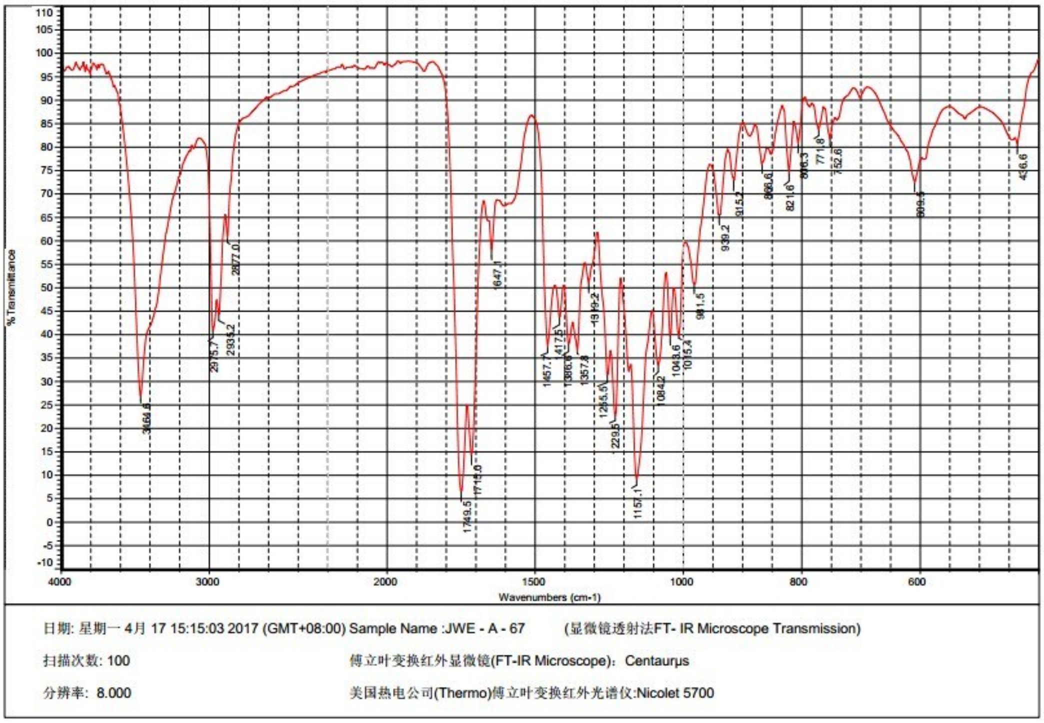

Fig. S5.8 IR spectrum of compound **5**

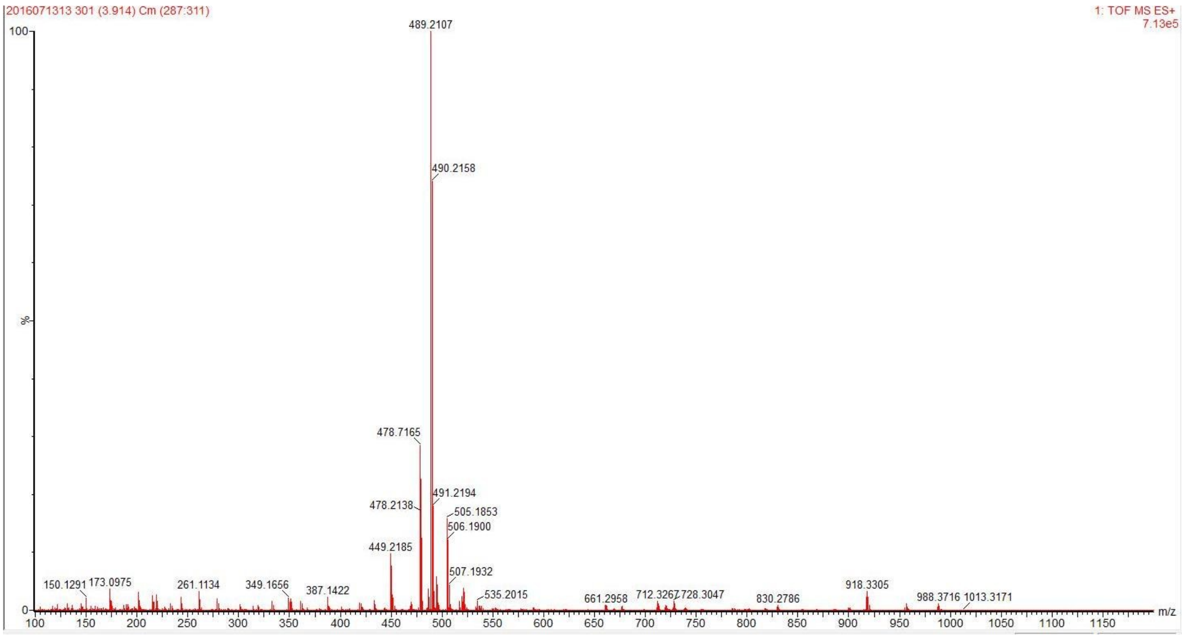

Fig. S5.9 HRESIMS spectrum of compound **5**

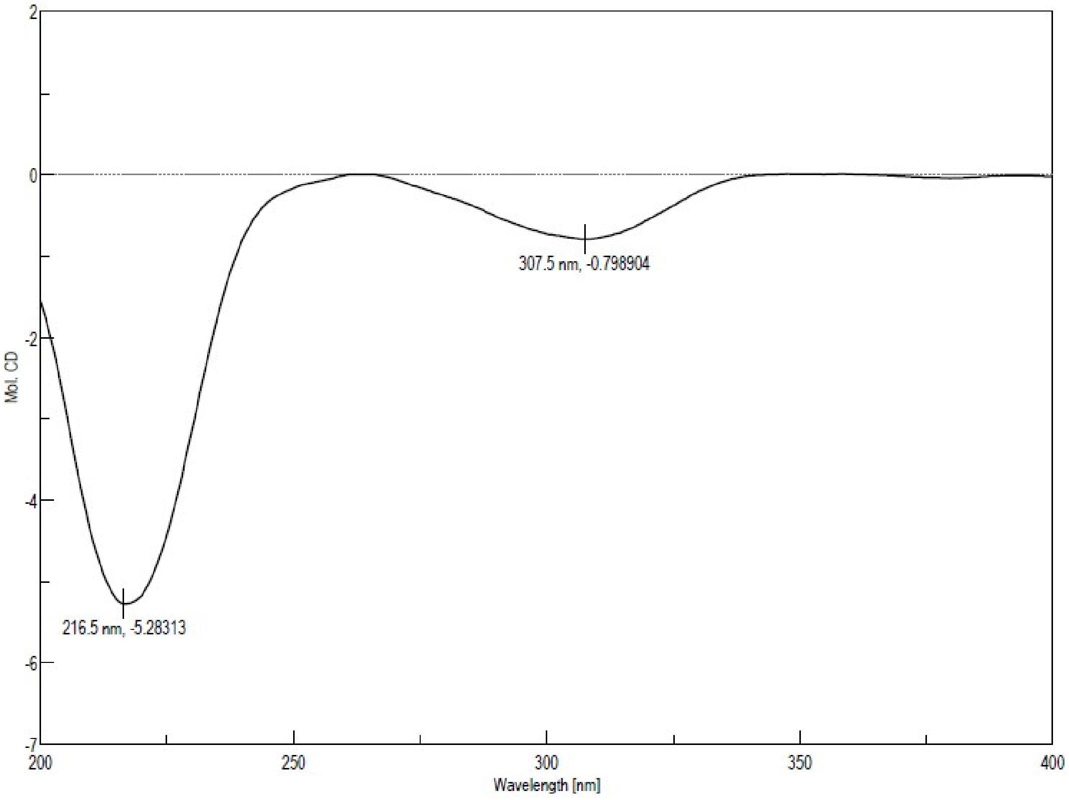

Fig. S5.10 CD spectrum of compound **5**

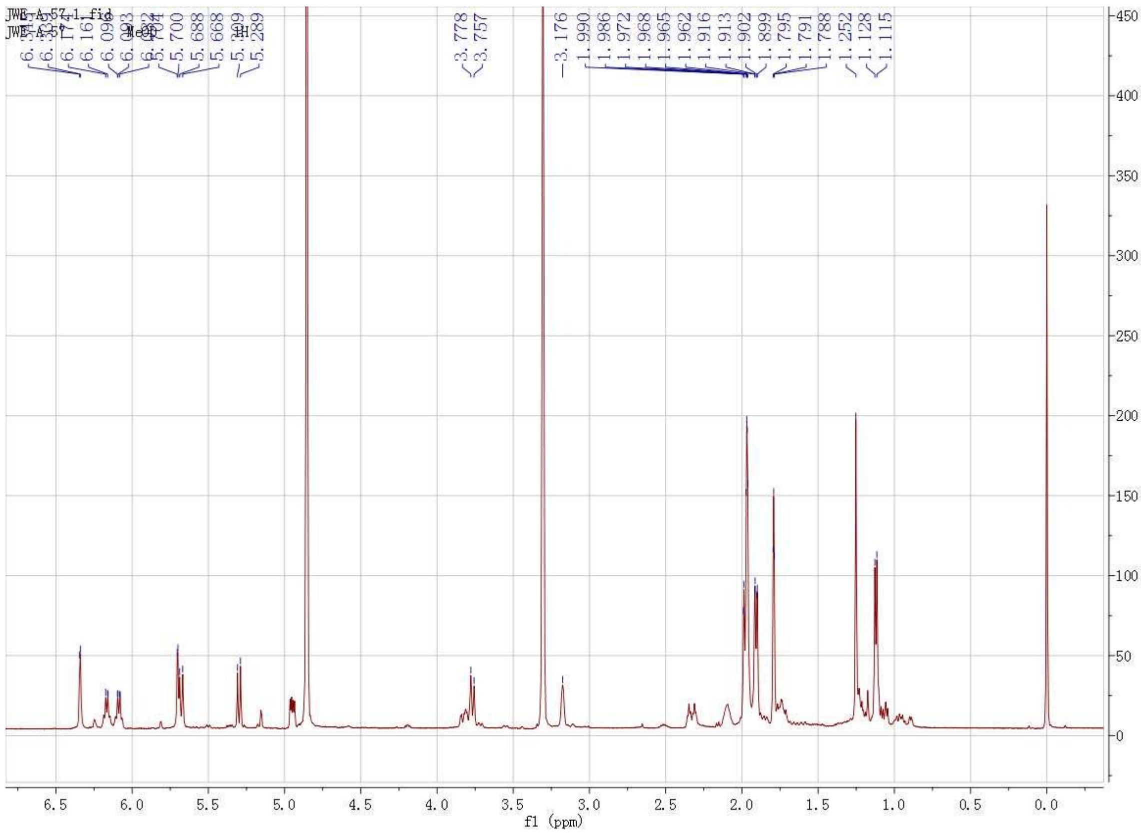

Fig. S6.1 ^{1}H NMR spectrum (500 MHz) of compound **6** in CD_3OD

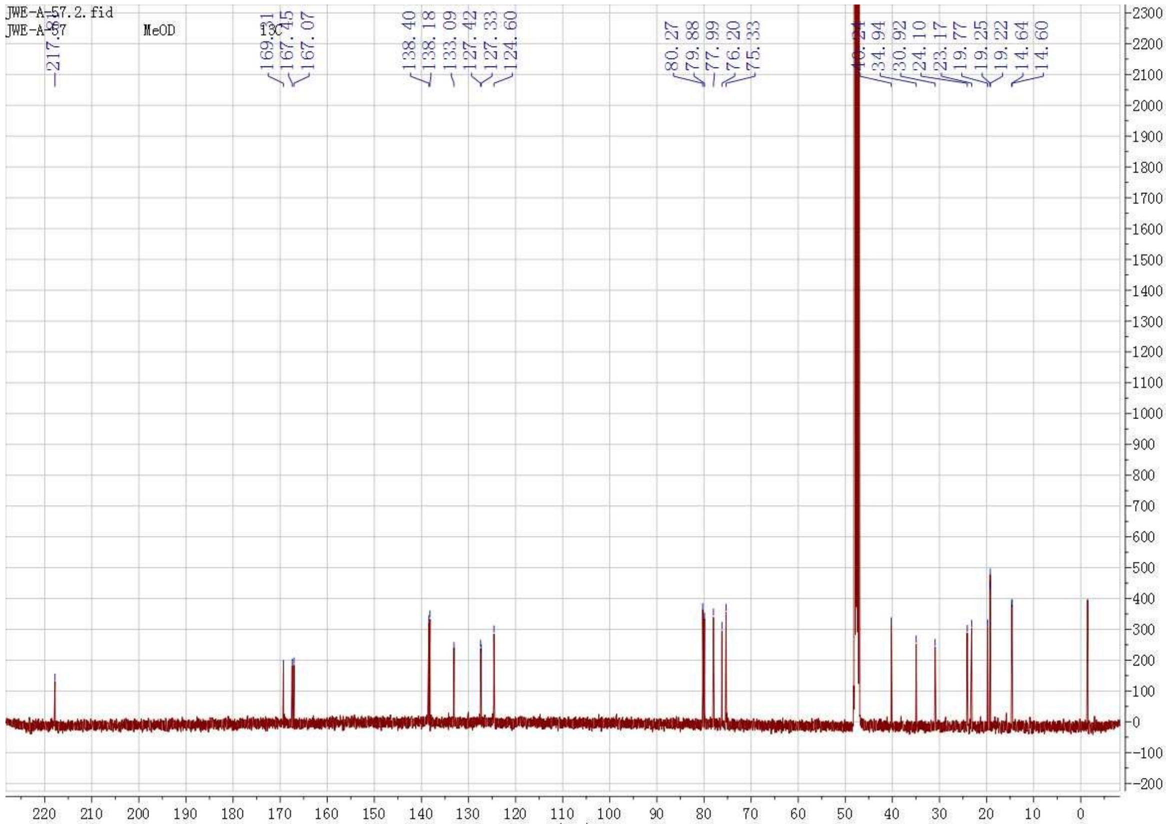

Fig. S6.2 ^{13}C NMR spectrum (125 MHz) of compound **6** in CD_3OD

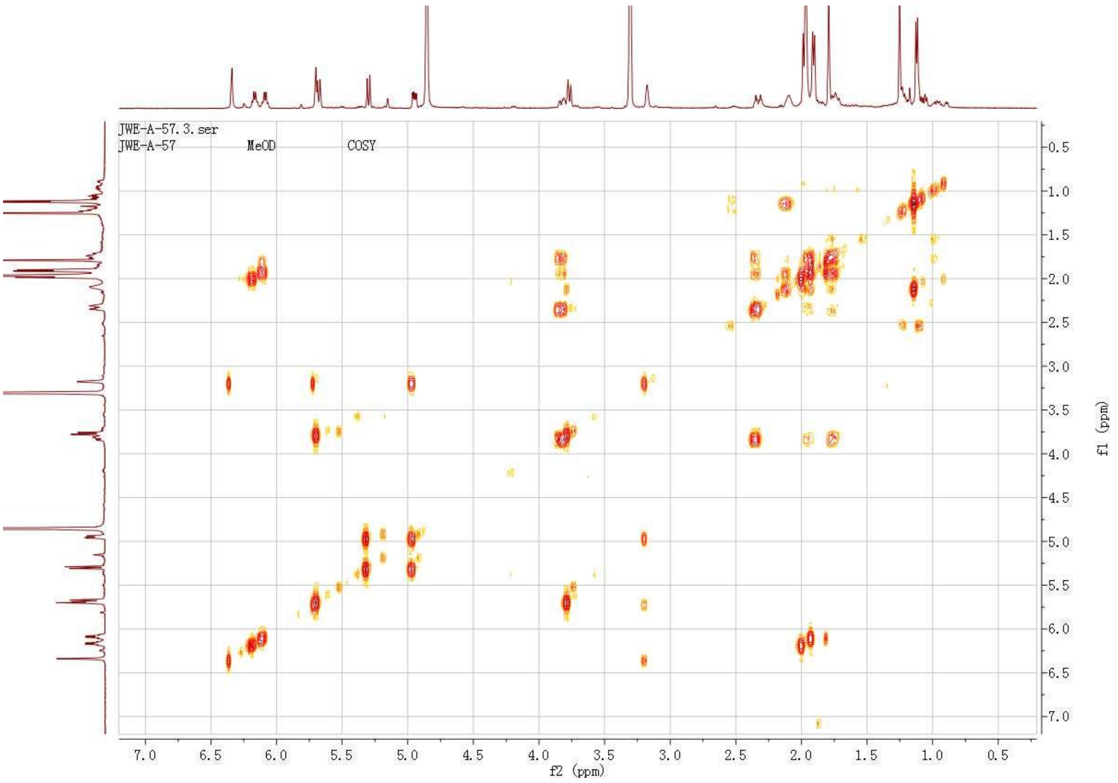

Fig. S6.3 ^{1}H-^{1}H COSY spectrum (500 MHz) of compound **6** in CD_3OD

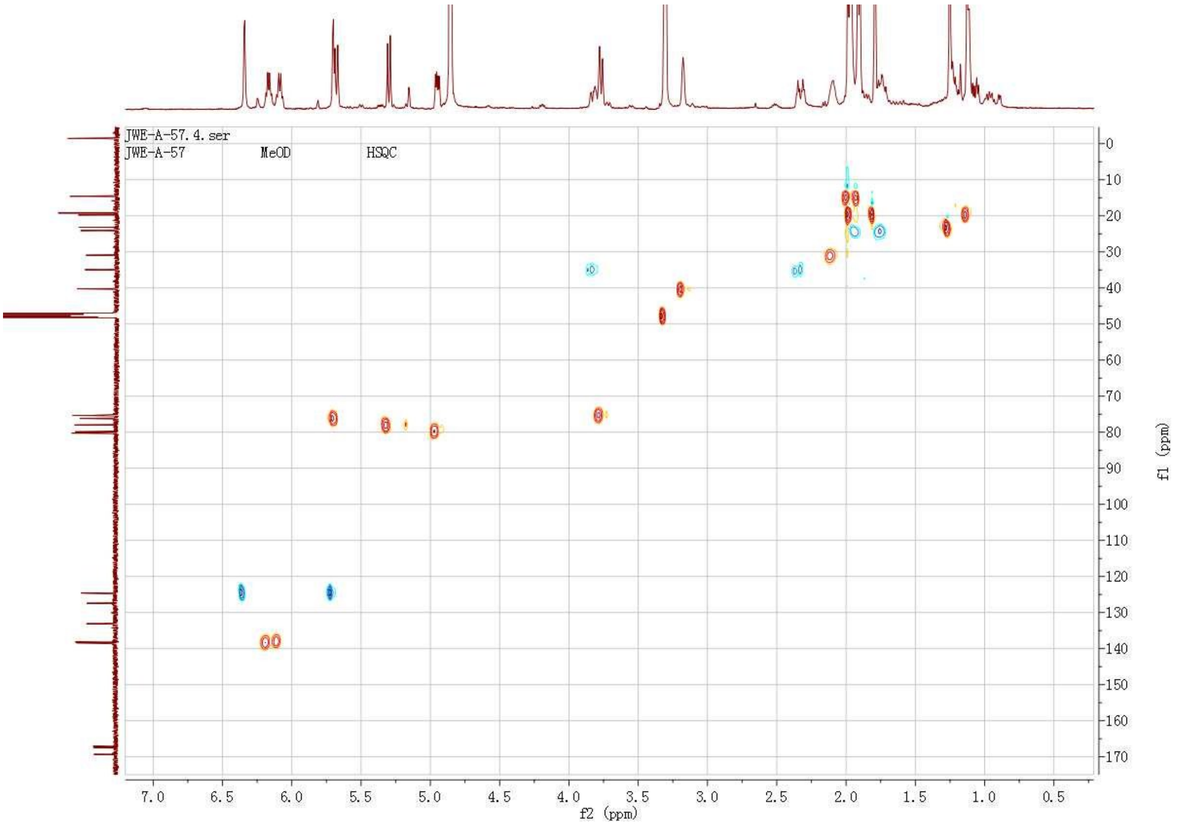

Fig. S6.4 HSQC spectrum (500 MHz) of compound **6** in CD_3OD

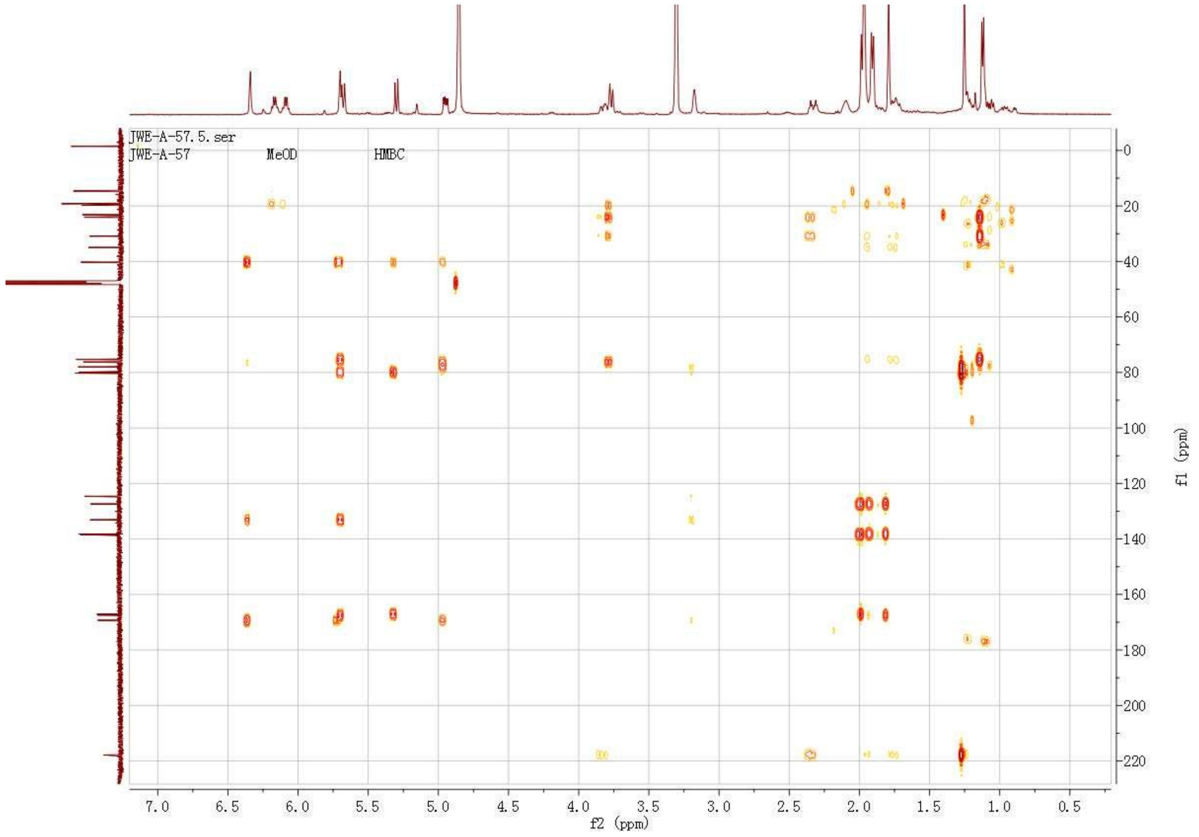

Fig. S6.5 HMBC spectrum (500 MHz) of compound **6** in CD_3OD

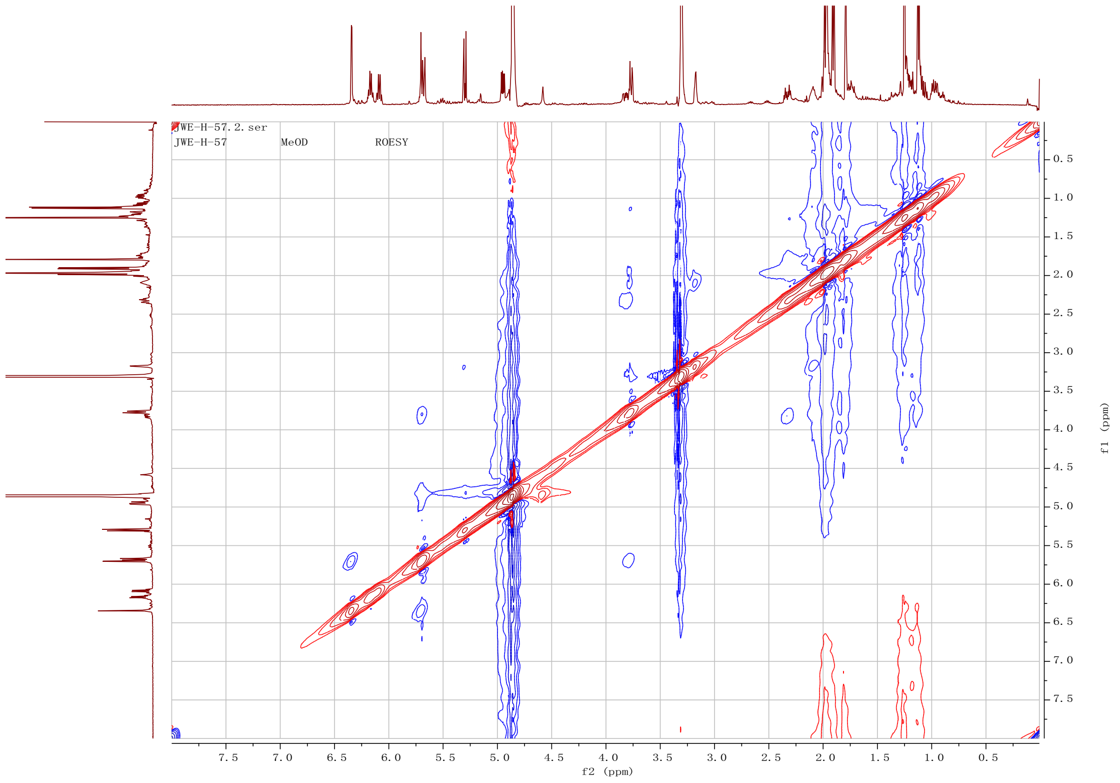

Fig. S6.6 ROESY spectrum (500 MHz) of compound **6** in CD_3OD

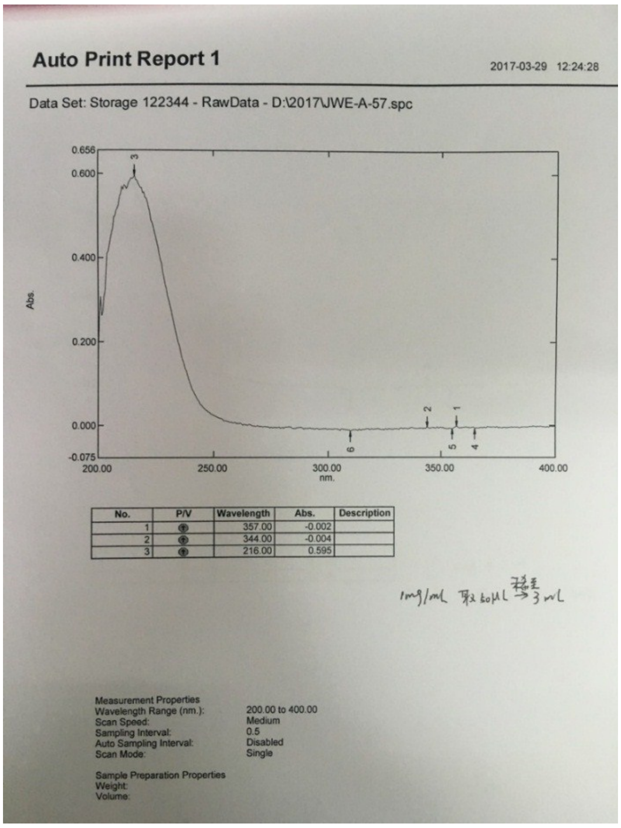

Fig. S6.7 UV spectrum of compound **6**

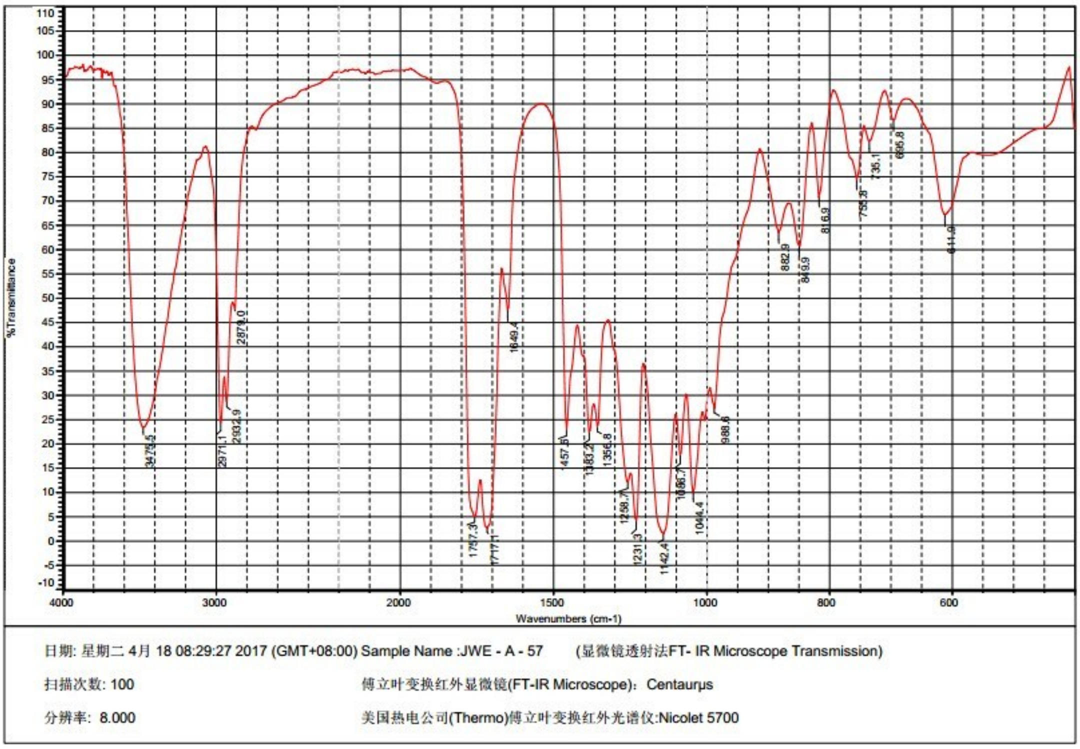

Fig. S6.8 IR spectrum of compound **6**

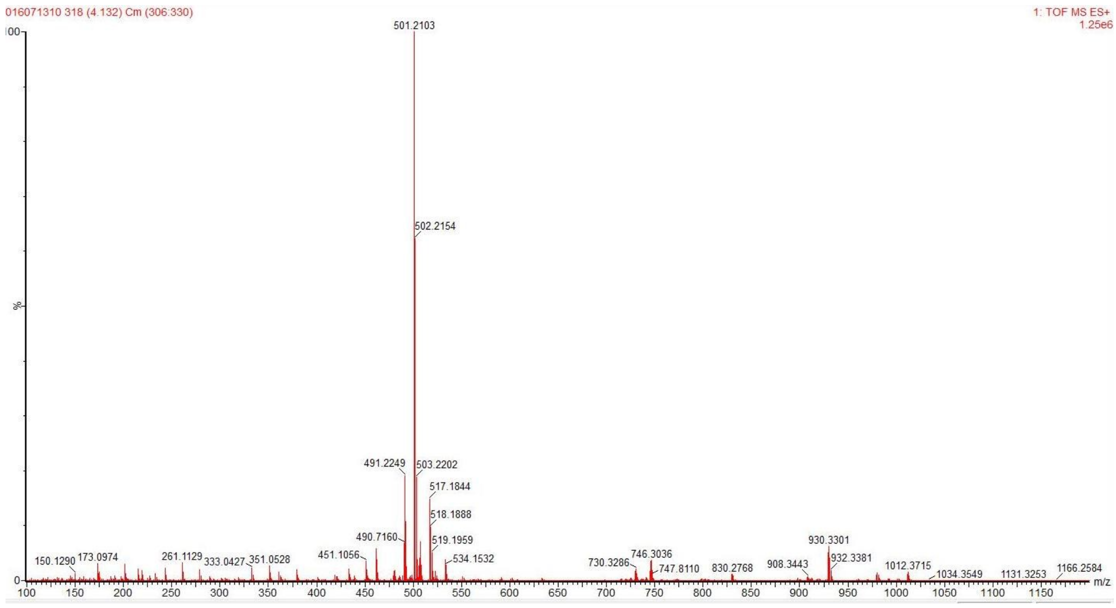

Fig. S6.9 HRESIMS spectrum of compound **6**

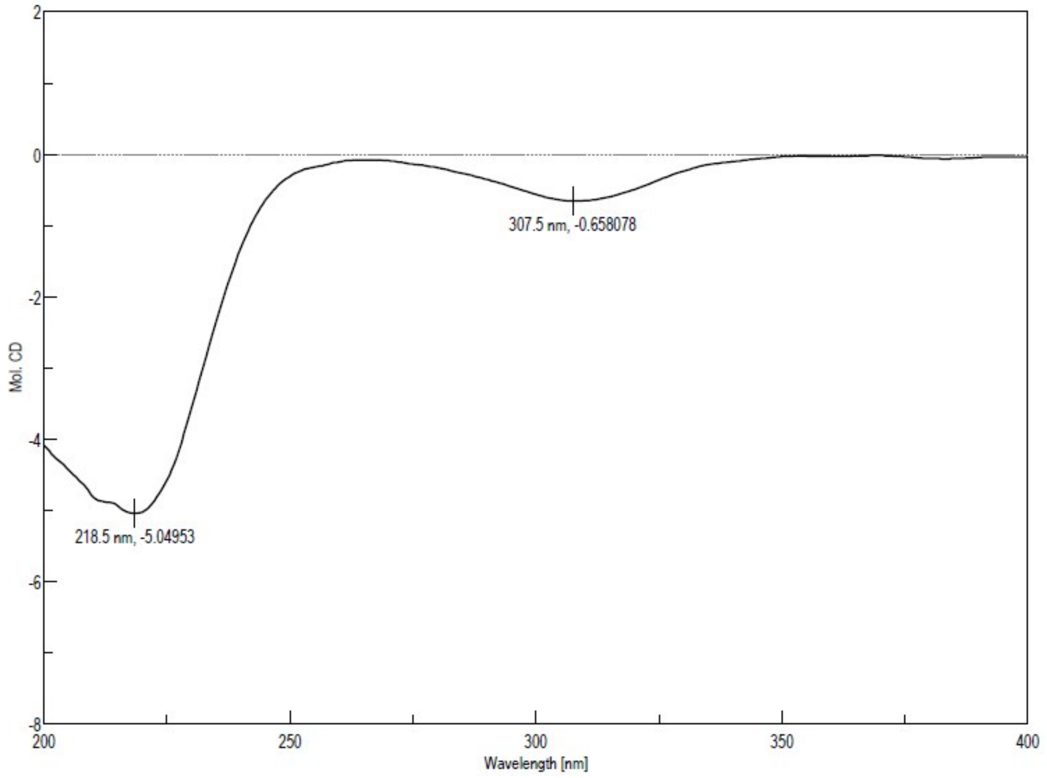

Fig. S6.10 CD spectrum of compound **6**

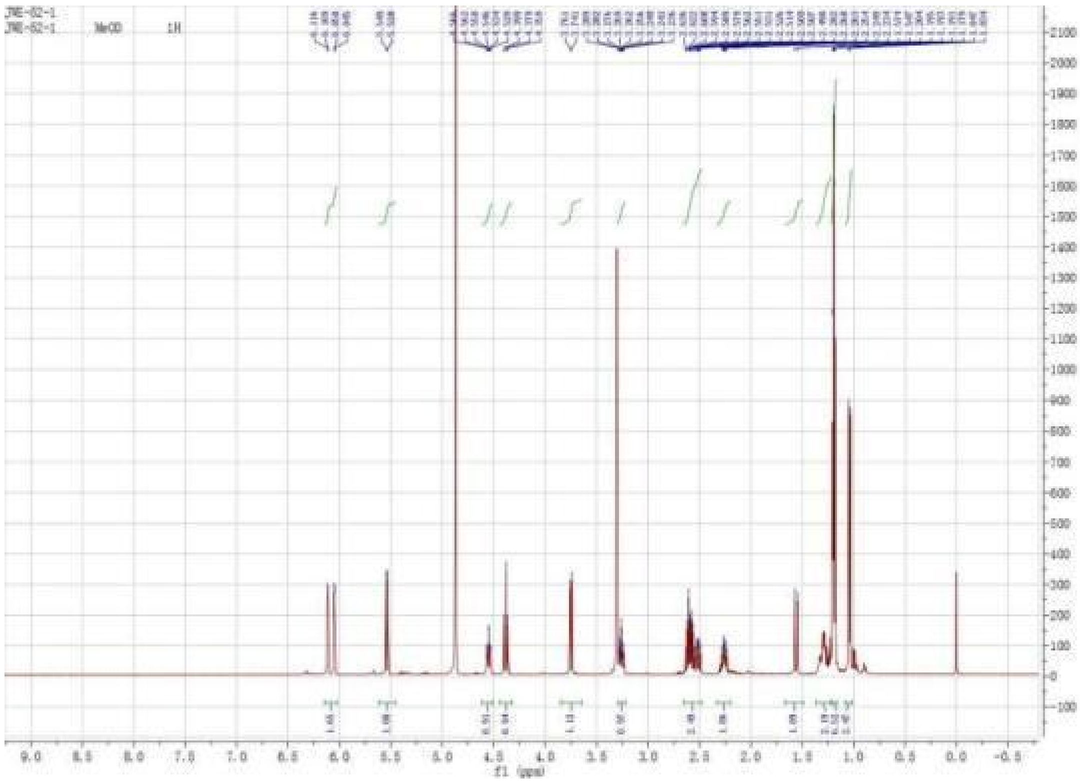

Fig. S7.1 ^{1}H NMR spectrum (500 MHz) of compound **7** in CD_3OD

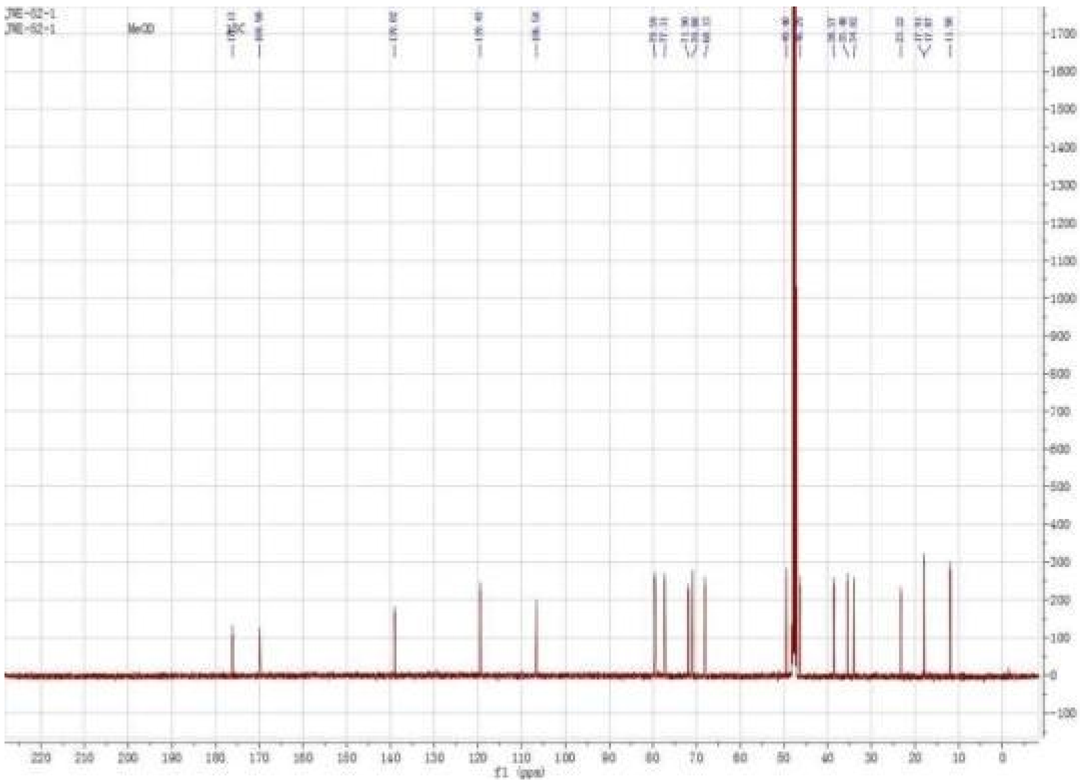

Fig. S7.2 ^{13}C NMR spectrum (125 MHz) of compound **7** in CD_3OD

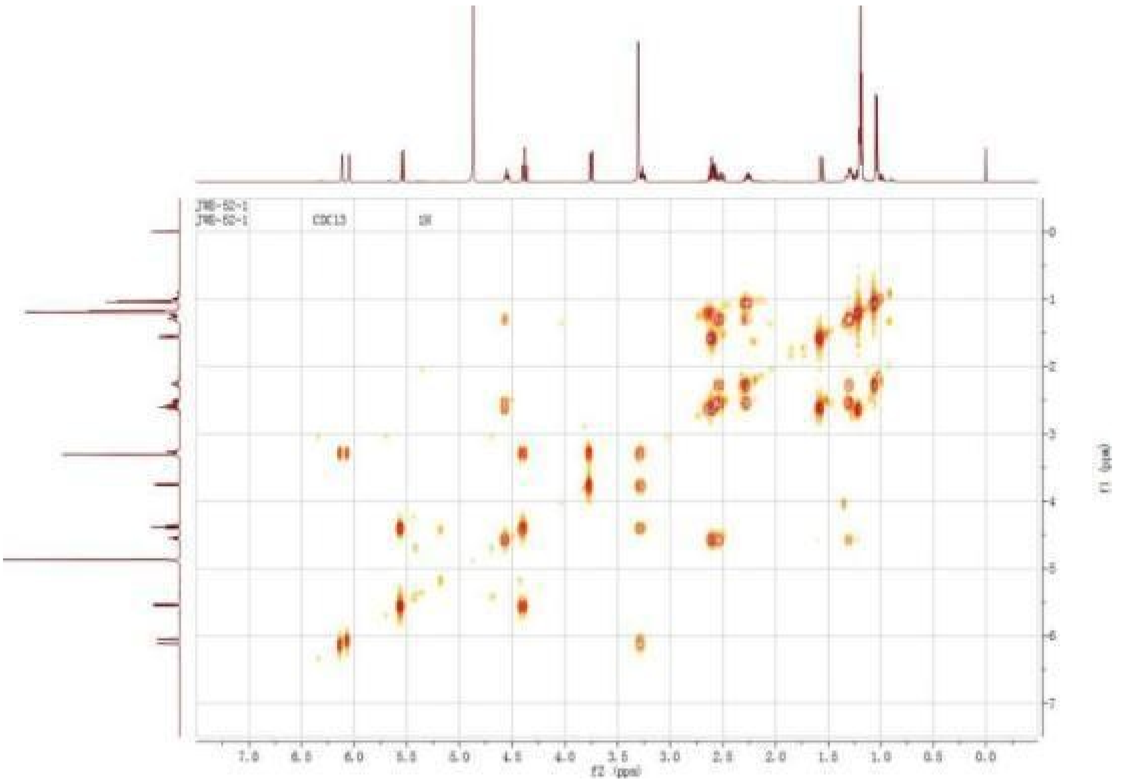

Fig. S7.3 ^{1}H-^{1}H COSY spectrum (500 MHz) of compound **7** in CD_3OD

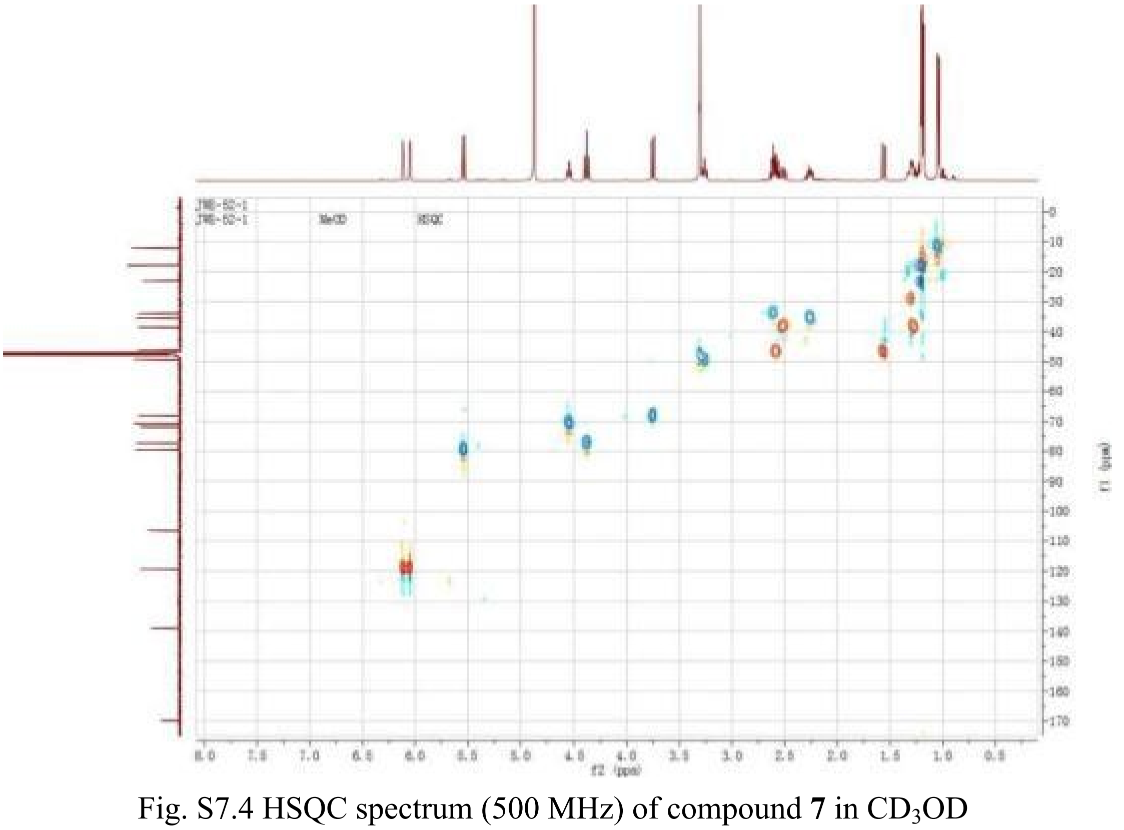

Fig. S7.4 HSQC spectrum (500 MHz) of compound **7** in CD_3OD

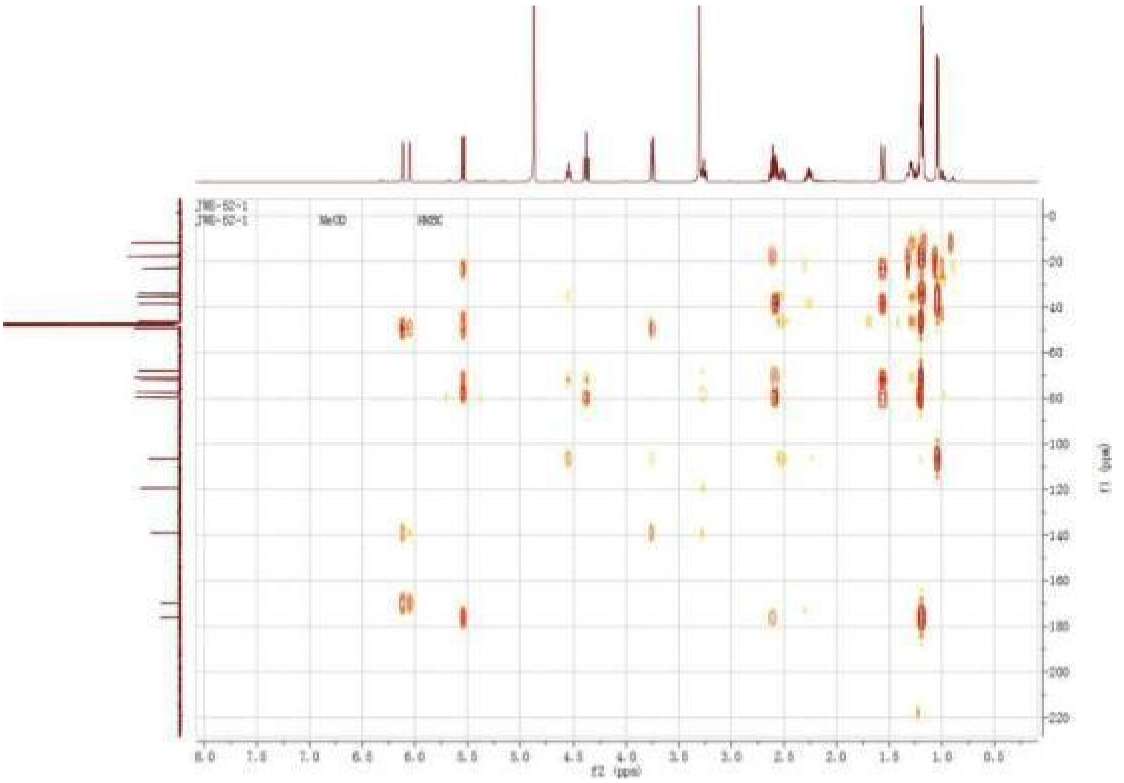

Fig. S7.5 HMBC spectrum (500 MHz) of compound **7** in CD_3OD

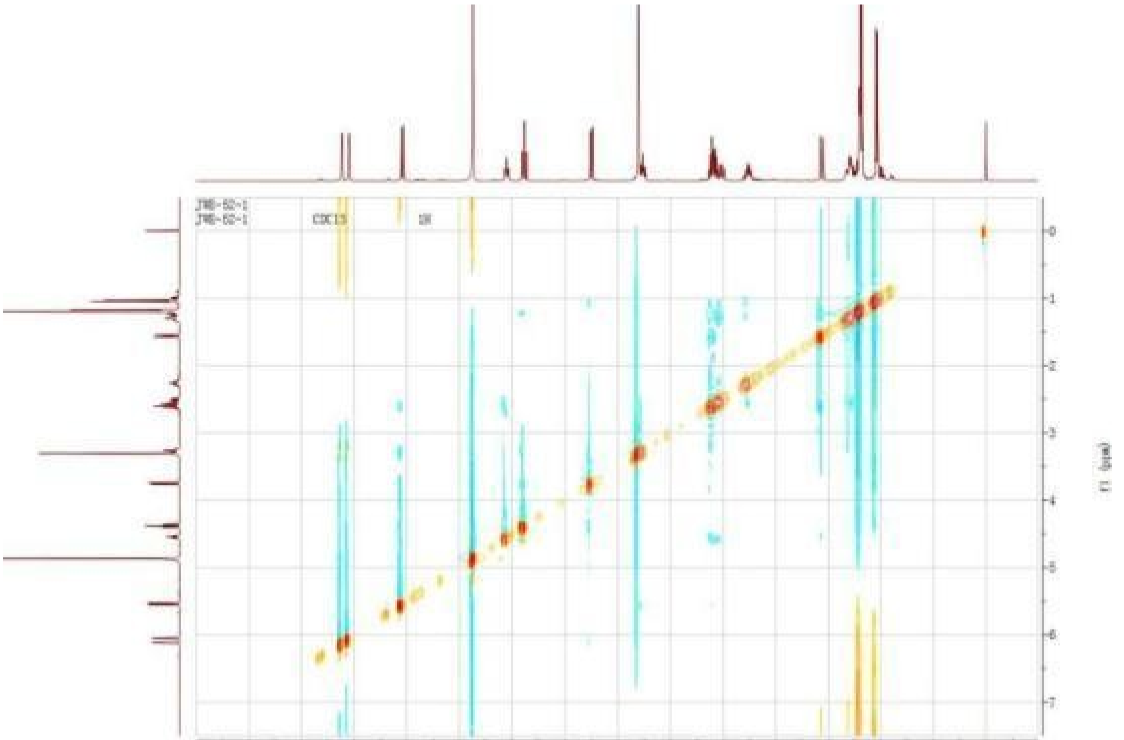

Fig. S7.6 ROESY spectrum (500 MHz) of compound **7** in CD_3OD

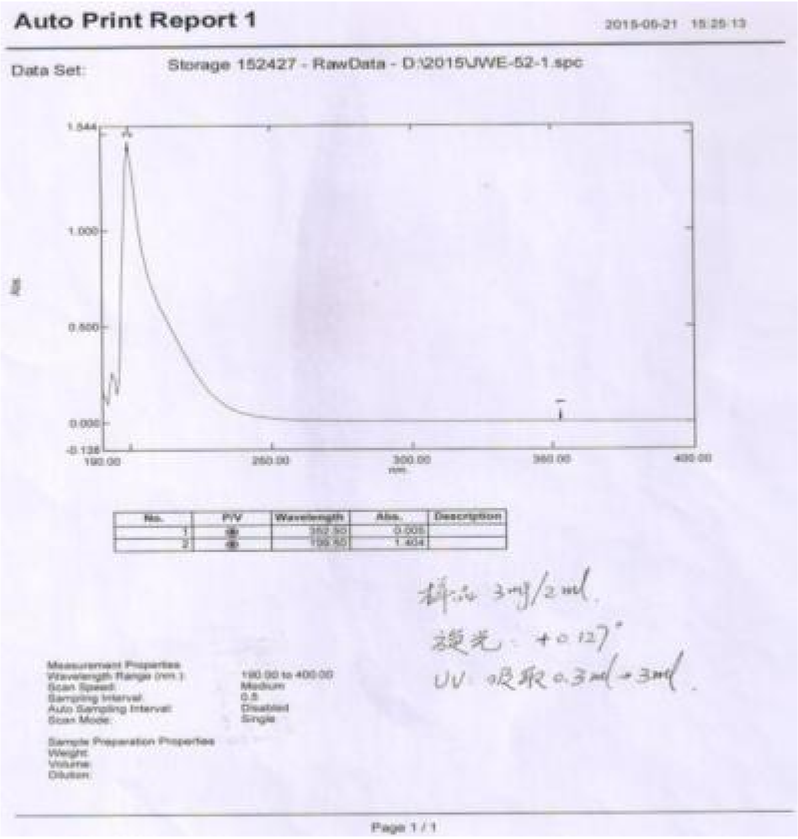

Fig. S7.7 UV spectrum of compound **7**

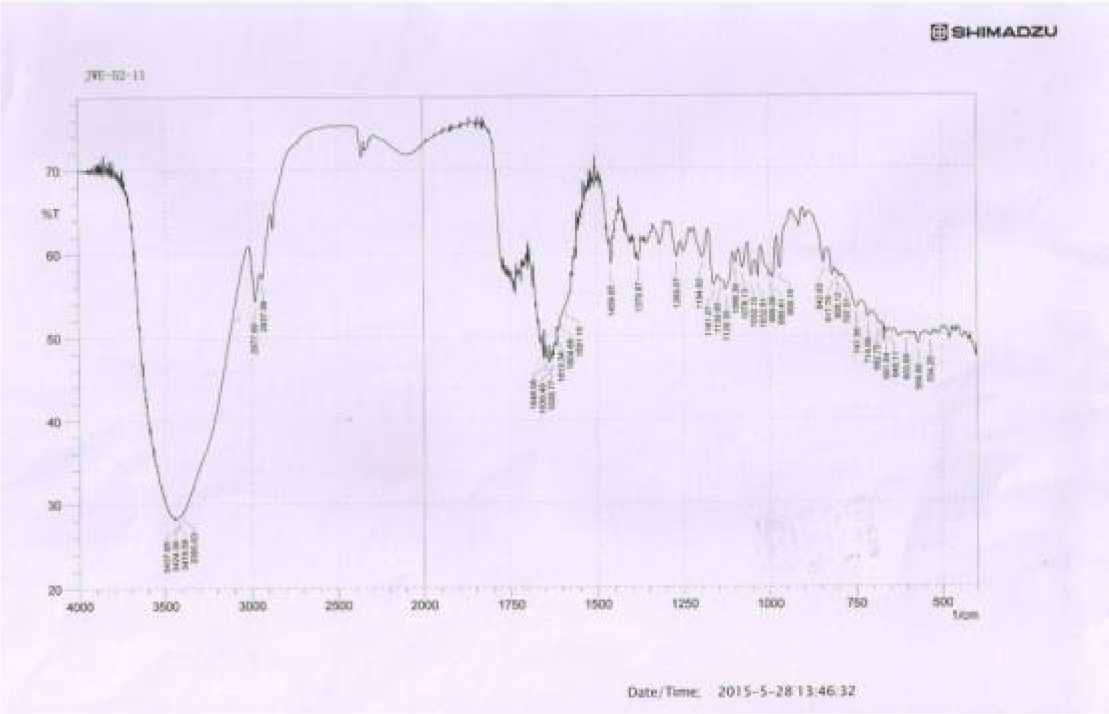

Fig. S7.8 IR spectrum of compound **7**

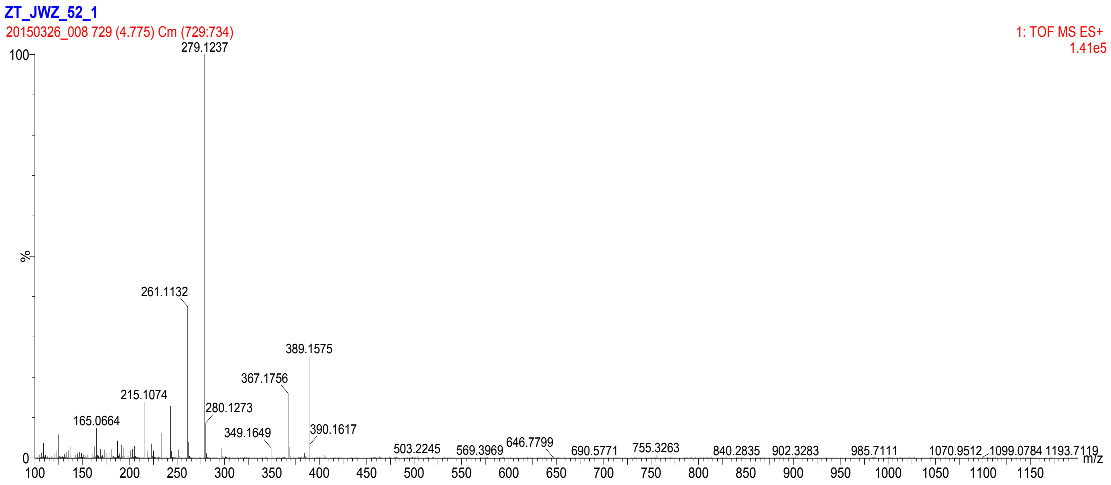

Fig. S7.9 HRESIMS spectrum of compound **7**

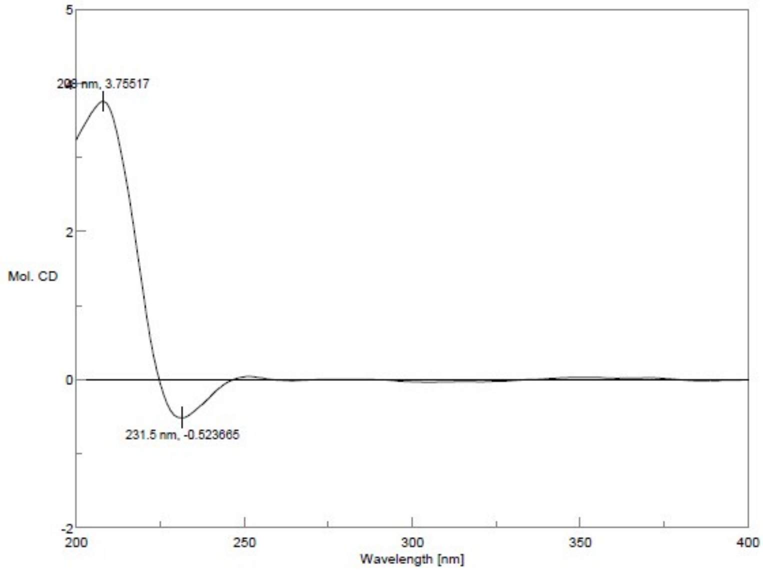

Fig. S7.10 CD spectrum of compound **7**
